# Heterologous Production of the Marine Myxobacterial Antibiotic Haliangicin and Its Unnatural Analogues Generated by Engineering of the Biochemical Pathway

**DOI:** 10.1038/srep22091

**Published:** 2016-02-26

**Authors:** Yuwei Sun, Zhiyang Feng, Tomohiko Tomura, Akira Suzuki, Seishi Miyano, Takashi Tsuge, Hitoshi Mori, Joo-Won Suh, Takashi Iizuka, Ryosuke Fudou, Makoto Ojika

**Affiliations:** 1 Graduate School of Bioagricultural Sciences, Nagoya University, Furo-cho, Chikusa-ku, Nagoya 464-8601, Japan; 2 Center for Nutraceutical and Pharmaceutical Materials, Department of Bioscience and Bioinformatics, Myongji University, Yongin, Gyeonggido 449-728, Korea; 3 Institute for Innovation, Ajinomoto Co., Inc., Kawasaki, Kanagawa 210-8681, Japan; 4 R&D Planning Department, Ajinomoto Co., Inc., Chuo-ku, Tokyo 104-8315, Japan

## Abstract

Despite their fastidious nature, marine myxobacteria have considerable genetic potential to produce novel secondary metabolites. The marine myxobacterium *Haliangium ochraceum* SMP-2 produces the antifungal polyketide haliangicin (1), but its productivity is unsatisfactory. The biosynthetic gene cluster *hli* (47.8 kbp) associated with 1 was identified and heterologously expressed in *Myxococcus xanthus* to permit the production of 1 with high efficiency (tenfold greater amount and threefold faster in growth speed compared with the original producer), as well as the generation of bioactive unnatural analogues of 1 through gene manipulation. A unique acyl-CoA dehydrogenase was found to catalyse an unusual γ,δ-dehydrogenation of the diketide starter unit, leading to the formation of the terminal alkene moiety of 1. Biological evaluation of the analogues obtained through this study revealed that their bioactivities (anti-oomycete and cytotoxic activities) can be modified by manipulating the vinyl epoxide at the terminus opposite the β-methoxyacrylate pharmacophore.

Natural products derived from microorganisms have a long history of use as antibiotics for clinical use. As an alternative to conventional antibiotic producers such as *Streptomyces*, myxobacteria have attracted considerable attention, especially since the remarkable discovery of epothilones as microtubule-stabilizing agent^1^,^2^. Myxobacteria are unique Gram-negative proteobacteria that are characterized by gliding and by the formation of multicellular fruiting bodies^3^. They have larger genomes than other taxa of bacteria, and they can produce structurally diverse bioactive secondary metabolites having novel modes of action, making them a promising alternative source of new drugs^4^,^5^. Marine myxobacteria are particularly rare and difficult to handle^6^,^7^,^8^,^9^; consequently, their products have only recently been examined. Several groups of antibiotics from the marine environment have been identified, including haliangicins^10^,^11^,^12^, miuraenamides^13^,^14^, salimabromides, salimyxins, and enhygrolides.^15^,^16^ Haliangicin (**1**), a polyene-type polyketide with a unique terminal epoxy alkene group and β-methoxyacrylate pharmacophore (Fig. 1), was isolated as the first marine myxobacterial secondary metabolite from the halophilic myxobacterium *Haliangium ochraceum* SMP-2. It has been reported to be a potent fungicide that interferes with the electron flow within the cytochrome *b-c1* segment in fungal mitochondrial respiratory chains.^11^

Most myxobacterial metabolites are polyketides, nonribosomal peptides, or their hybrids; these are known to be biosynthesized by megasynthase polyketide synthases (PKSs) and nonribosomal peptide synthases (NRPSs). Although it has been reported that the sequences of the PKS genes in marine myxobacterial isolates are highly novel,^17^ biochemical studies on their metabolites have been hampered by unfavourable factors, such as difficulties in isolation, slow growth rates, cell aggregation, and poor metabolite productivity. In the case of haliangicin (**1**), the producer requires culture periods in excess of two weeks to reach maximal production of **1**, and typically produces only about 1 mg L^−1^. Furthermore, the lack of a well-developed genetic methodology applicable to the marine strains is another major obstacle. Therefore, biosynthetic studies on these fastidious marine myxobacteria are still at an early stage, and they remain quite challenging, although attractive.

Here, we describe the cloning and identification of the haliangicin biosynthetic machinery and its use as the first representative of a marine myxobacterial biosynthetic assembly line. The establishment of a heterologous expression system allowed us to optimize the production titre, to manufacture unnatural analogues, and to analyse the function of the biosynthetic enzymes, which would be difficult to achieve with the native producer of **1**.

## Results

### Identification and heterologous expression of the haliangicin biosynthetic gene cluster (*hli*)

**Figure 2:**
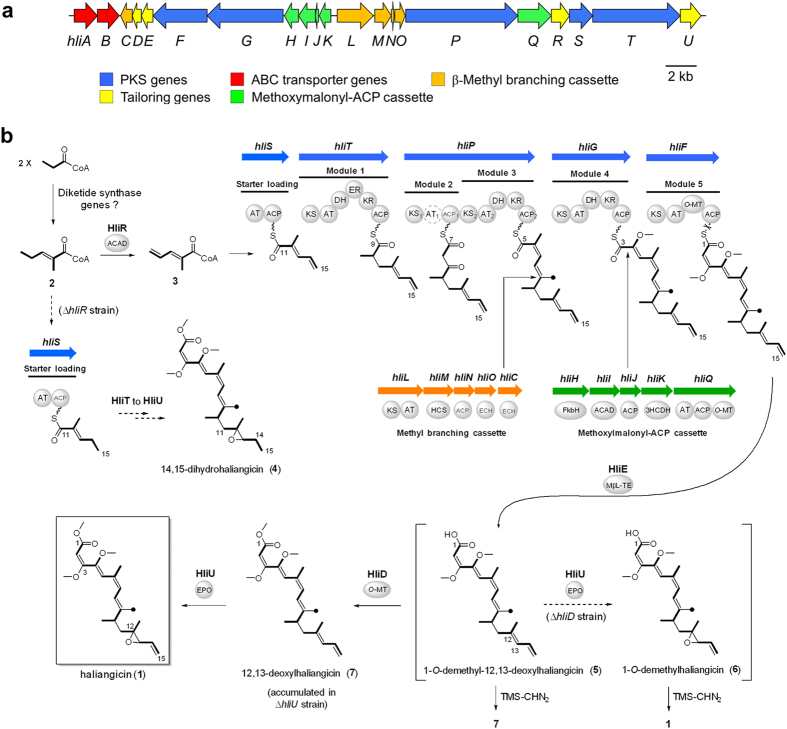
Haliangicin biosynthetic machinery. (**a**) Genetic organization of the haliangicin biosynthetic gene cluster (*hli*). (**b**) A proposed biosynthetic pathway for haliangicin (**1**). AT_1_ in module 2 is inactive, but no complementary *trans*-AT is found in *hli*. The dashed arrows indicate the unnatural pathways in the gene-disrupted strains of the heterologous host *M. xanthus* c10-11C/c7-6E. *Abbreviations*: ACAD, acyl-CoA dehydrogenase; ACP, acyl carrier protein; AT, acyl transferase; DH, dehydratase; ECH, enoyl-CoA hydratase; EPO, epoxidase; ER, enoyl reductase; FkbH, FkbH-like protein; HCS, HMG-CoA synthase; KR, ketoreductase; KS, ketosynthase; MβL-TE, metallo β-lactamase-type thioesterase; O-MT, O-methyltransferase; 3HCDH, 3-hydroxyacyl-CoA dehydrogenase.

The polyene structure of haliangicin (**1**) suggests that its biosynthetic machinery is a pure PKS system that is accompanied by various enzymes involved in tailoring modifications. To clone the biosynthetic gene cluster responsible for formation of haliangicin, we first constructed a cosmid library of the *H. ochraceum* genome by using the *E. coli/Streptomyces* shuttle vector pDW103^18^. The resulting library, consisting of approximately 1700 cosmid clones, was then screened by colony hybridization using probes homologous to the ketosynthase (KS) genes of terrestrial myxobacteria (Supplementary Fig. S1). The end sequences of several KS-positive cosmids indicated that they might be responsible for the biosynthesis of **1** (Supplementary Fig. S2); these included HMG-CoA synthase for installing a β-branch methyl (9-Me) group and a 3-hydroxyacyl-CoA dehydrogenase for biosynthesizing methoxymalonyl-ACP, a precursor of the 1,2-dioxy unit at the C-3–C-4 position. Of the cosmids that were obtained, two partially overlapping cosmids, c7-6E and c10-11C, were sequenced, revealing a continuous 63-kbp segment that putatively harbours the entire haliangicin biosynthetic gene cluster (*hli*). Sequence annotation suggested that the *hli* gene cluster (GC content 67.2%) spans 47.8 kbp and contains 21 open reading frames (Fig. 2a, Table 1 and Supplementary Tables S1–S3). We assigned these genes to *hliA-U*.

Five Type-I PKS genes (*hliF*, *G*, *P*, *S* and *T*) were predicted to be responsible for the construction of the haliangicin backbone. Other genes could be assigned to a β-methyl branching cassette (*hliL*, *M*, *N*, *O*, and *C*), a methoxymalonyl-ACP cassette (*hliQ*, *H*, *I*, *J*, and *K*), and modification genes encoding an O-methyltransferase (HliD), a metallo-β-lactamase-type thioesterase (HliE), an acyl-CoA dehydrogenase (HliR), and an epoxidase (HliU). As discussed below, there were particular features in the gene cluster, for example, a rather disorganized gene arrangement (scattered PKS genes and segmented gene cassettes) and the presence of insufficient modules to construct the haliangicin backbone.

**Figure 3:**
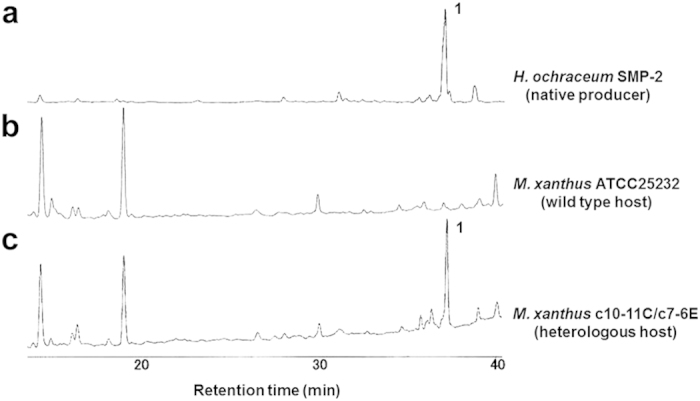
Production of haliangicin (1) by the heterologous host. Reversed-phase HPLC of extracts of the native producer (**a**), the wild-type host *M. xanthus* (**b**), and the heterologous host harbouring the *hli* gene cluster (**c**).The chromatograms were recorded at 290 nm.

Demonstration that this gene cluster is responsible for the biosynthesis of haliangicin was hampered by the slow growth and a severe tendency for cell aggregation of the native producer. We therefore set out to express the biosynthetic machinery for **1** heterologously in a more readily cultivated and more tractable host. Our initial attempt to express the *hli* genes in a *Streptomyces* sp. resulted in transformants that showed no titre of haliangicin (**1**). Finally, we found that *Myxococcus xanthus*, a fast-growing terrestrial myxobacterium,^19^,^20^,^21^ is a suitable host. The aforementioned cosmids c7-6E and c10-11C, corresponding to the upstream and downstream parts of *hli*, respectively, were each modified through λ-Red recombineering^22^ (Supplementary Fig. S3 and S4) and integrated into the chromosome of *M. xanthus* ATCC25232 in a stepwise manner to reconstitute the haliangicin biosynthetic pathway (Supplementary Fig. S5–S7). The constructed strain, which we named *M. xanthus* c10-11C/c7-6E, was found to produce haliangicin (**1**) successfully, as revealed by HPLC (Fig. 3), verifying that the *hli* gene cluster is responsible for haliangicin biosynthesis. It is intriguing and worth mentioned that the heterologous host did not produce significant quantities of the 12,13-*cis*-epoxy isomer (*cis*-haliangicin), which usually accompanies **1** in the original producer (Fig. S8).

Next, we took advantage of the heterologous producer of **1**, *M. xanthus* c10-11C/c7-6E, to settle several issues that were difficult to resolve with the native haliangicin producer.

### Optimization of the heterologous production of haliangicin based on the identified biosynthetic precursors

**Figure 4:**
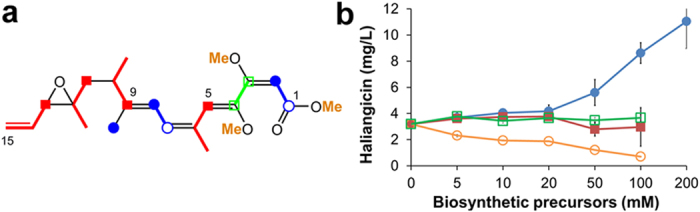
Biosynthetic precursors and their effects on the production of haliangicin (1). (**a**) Building blocks deduced from feeding experiments (red: [1-^13^C]propionate; blue: [1,2-^13^C_2_]acetate; green: [U-^13^C_3_]glycerol; orange: _L_-[methyl-^13^C]methionine). (**b**) Optimization of the production of **1** by feeding the appropriate biosynthetic precursors indicated by the same colours as in Fig. 4a. The fermentation was performed for 5 days.

The biosynthetic units for haliangicin (**1**) were first examined. The producer was fed various^13^C-labelled precursors, and the labelled haliangicins produced were analysed by NMR spectroscopy (Supplementary Fig. S9). This revealed that two acetate units, one acetate-derived methyl branch (9-Me), four propionate units, and three *S*-adenosylmethionine-derived *O*-methyl carbon atoms had been incorporated into **1** (Fig. 4a). The remaining two-carbon unit (C3–C4) bearing vicinal oxygen atoms was found to originate from glycerol, as supported by the incorporation of two carbon atoms from [U-^13^C_3_]glycerol (Supplementary Fig. S9e). Such a unit is known as a glycolate extender in some metabolites.^23^,^24^,^25^

Secondly, we evaluated the effects of these biosynthetic precursors on the productivity of haliangicin (**1**) (Fig. 4b). The haliangicin titre increased significantly when sodium acetate was added at a concentration higher than 50 mM and finally reached 11.0 ± 2.1 mg L^–1^ at 200 mM of sodium acetate after 5 days. The efficiency of haliangicin production was eleven times higher in quantity and three times shorter in culture period than that achieved with the native producer *H. ochraceum*.

### Proposed mechanism for the biosynthesis of haliangicin

The use of the heterologous host for **1** also made gene manipulation much easier, allowing us not only to acquire a complete picture of the somewhat irregular biosynthetic machinery for **1**, but also to generate several new analogues of haliangicin. On the basis of the deduced architecture of the *hli* gene cluster, we propose the haliangicin biosynthetic route shown in Fig. 2b. From the viewpoint of conventional PKS biosynthesis, the construction of the heptaketide backbone of **1** is likely to require six elongation steps following a C3 starter-loading step, but the *hli* machinery possesses only five elongation modules (modules 1–5 in Fig. 2b). An iterative function of any particular PKS is unlikely, because all the KS domains in *hli* are classified as modular not iterative (Supplementary Fig. S10). One possible solution to this problem of a missing module might be the direct use of a diketide (C_6_) starter, such as 2-methylpent-2-enoyl-CoA (**2**) (Fig. 2b and Supplementary Fig. S11), which is suggested by a putative diketide synthase found in the genomes of *H. ochraceum* and *M. xanthus* (Supplementary Fig. S12).

The diketide precursor **2** might undergo γ,δ-dehydrogenation by HliR, a particular acyl-CoA dehydrogenase, to form 2-methylpent-2,4-dienoyl CoA (**3**), which might serve as a starter for the formation of the unique 14,15-terminal olefin of **1**. A detailed functional analysis of HliR will be described later. The diene starter **3**, prepared by HliR, is next loaded into the typical starter-loading module encoded by *hliS*, and the five PKS-mediated elongation steps take place sequentially to generate the haliangicin backbone (Fig. 2b). Sequence analysis showed that the domain arrangements of the PKSs corresponded well with the chemical structure of haliangicin (**1**). The module 2-mediated elongation step is followed by the formation of β-methyl branch at the 9-position by the methyl-branching cassette^26^,^27^ (HliC and HliL–O) (Supplementary Fig. S13). Module 4 incorporates a glycolate extender, biosynthesized by the methoxymalonyl-ACP cassette^24^,^28^ (HliH–K and HliQ) (Supplementary Fig. S14), to construct the vicinal oxygen functionality at C3–C4. Finally, the nascent carboxylic acid released by HliE, which is homologous to a metallo-β-lactamase-type thioesterase,^29^,^30^ undergoes two post-assembly modification steps: O-methylation at the carboxyl terminus by HliD and epoxidation at the diene terminus by HliU (Fig. 2b).

### Verification of some biosynthetic genes and generation of unnatural analogues of haliangicin

**Figure 5:**
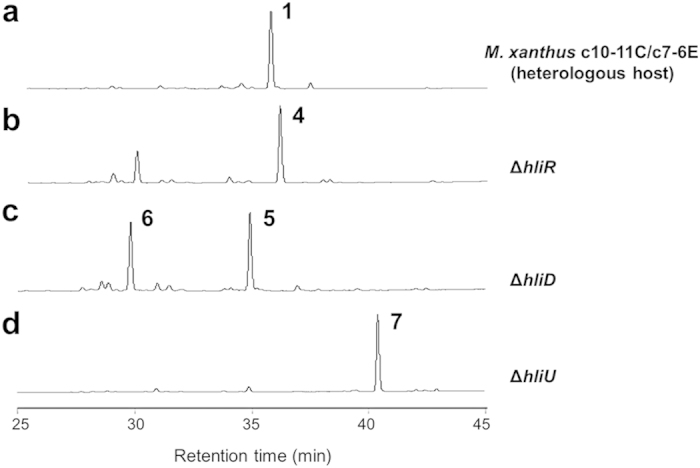
Generation of unnatural haliangicin analogues in the gene-disrupted heterologous hosts. HPLC analyses of extracts of the heterologous producer *M. xanthus* c10-11C/c7-6E (**a**), Δ*hliR* (acyl-CoA dehydrogenase disrupted host, (**b**), Δ*hliD* (O-methyltransferase-disrupted host, (**c**), and Δ*hliU* (epoxidase disrupted host, (**d**). The chromatograms were recorded at 290 nm.

By using the genetically amenable host producer, we manipulated three genes involved in the formation of haliangicin to validate the proposed biosynthetic pathway discussed above and to produce several unnatural analogues of **1** (Fig. 5). First, we targeted the acyl-CoA dehydrogenase gene *hliR,* which plays an important role in the early stages of haliangicin biosynthesis. Disruption of *hliR* successfully generated the first unnatural analogue 14,15-dihydrohaliangicin (**4**), clearly demonstrating that *hliR* is responsible for the formation of the terminal olefin moiety (Supplementary Fig. S15). Subsequently, we inactivated two post-assembly tailoring enzymes: HliD (O-methyltransferase) and HliU (epoxidase). Inactivation of *hliD* led to the production of two free-acid intermediates, 1-*O*-demethyl-12,13-deoxyhaliangicin (**5**) and 1-*O*-demethylhaliangicin (**6**) as confirmed by LC-MS (Supplementary Fig. S16). However, because of their labile nature, these acids could not be purified for NMR analyses; consequently, their structures were chemically confirmed by methylation with trimethylsilyldiazomethane to give 12,13-deoxyhaliangicin (**7**) and haliangicin (**1**), respectively (Supplementary Fig. S17). The accumulation of 1-*O*-demethylhaliangicin (**6**) in the Δ*hliD* strain suggests that the free acid 1-*O*-demethyl-12,13-deoxyhaliangicin (**5**) also undergoes epoxidation by HliU. Finally, as expected, inactivation of *hliU* caused 12,13-deoxyhaliangicin (**7**) to accumulate without the formation of **1** (Supplementary Fig. S18). These results provide convincing confirmatory evidence for our proposed haliangicin biosynthetic machinery.

### Functional analysis of dehydrogenase HliR responsible for terminal alkene formation

**Figure 6:**
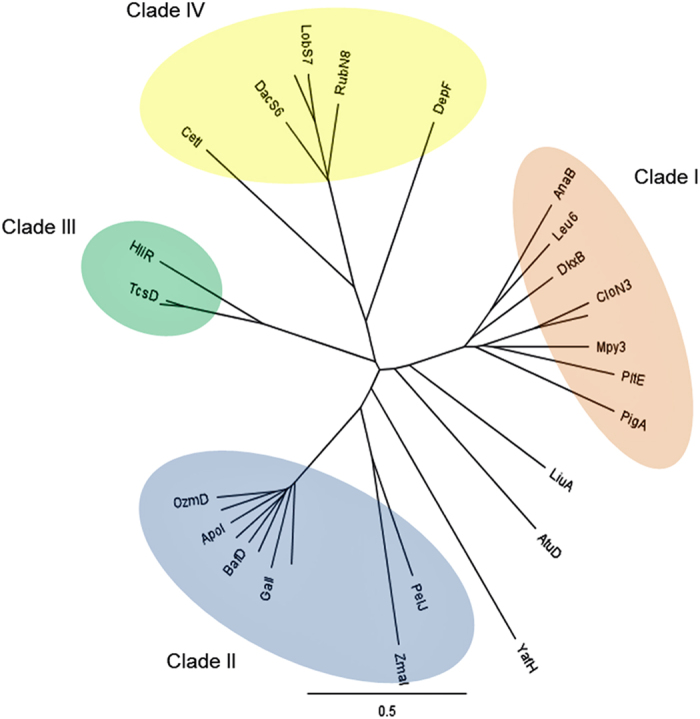
Phylogenetic tree of bacterial acyl-CoA dehydrogenase (ACAD)-related oxidoreductases obtained by using the NJ method. HliR is classified as Clade III with TcsD from the FK506 pathway. Clade I: l-prolyl-*S*-PCP dehydrogenase; Clade II: methoxymalonyl-ACP biosynthesis involved oxygenase; Clade III: propylmalonyl-ACP dehydrogenase; Clade IV: nitrosugar biosynthesis-related nitrososynthase.

**Figure 7:**
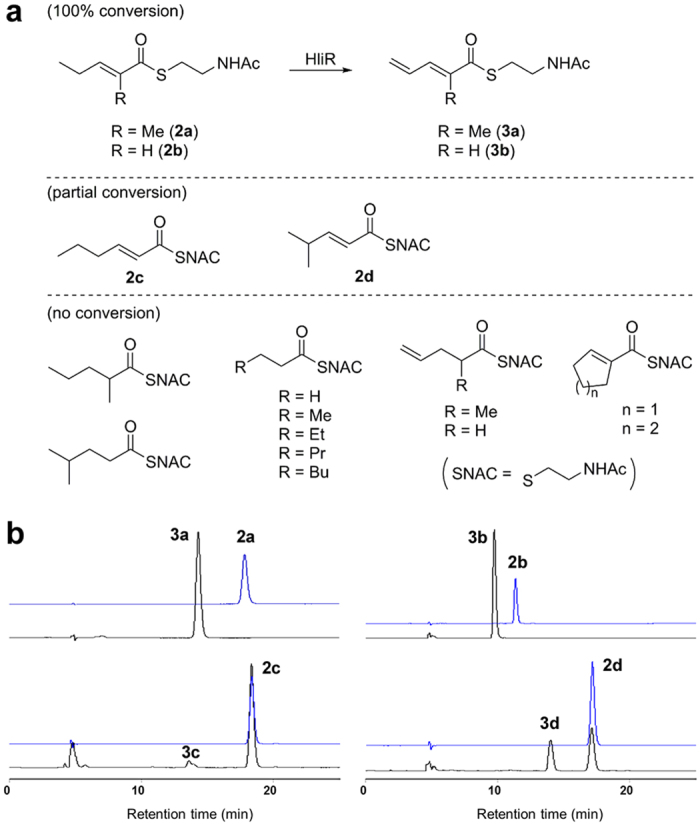
Functional analyses of HliR, an acyl-CoA dehydrogenase. (**a**) Substrate specificity of recombinant acyl-CoA dehydrogenase HliR. (**b**) *In vitro* dehydrogenation of acyl-SNAC compounds by HliR. HPLC analyses of the enzymatic reaction mixtures (black lines) with the substrates **2a**−**d**. The chromatograms were recorded at 254 nm.

Terminal alkene structures are found in a few natural products, for example, curacin A,^31^,^32^ tautomycetin,^33^ and FK506^34^ The unique terminal vinyl epoxide alkene moiety in haliangicin (**1**) prompted us to elucidate the biosynthetic mechanism responsible for its formation. Because *hliR* is homologous to acyl-CoA dehydrogenases, the enzyme encoded by this gene might also be a good candidate for introducing the double bond between C-14 and C-15. Actually, the aforementioned gene disruption of *hliR* prevented the formation of **1** and resulted in the production of analogue **4** with a saturated terminus, thereby providing unequivocal evidence of the direct involvement of *hliR*. A detailed sequence and phylogenetic analysis indicated that HliR is homologous to short-chain acyl-CoA dehydrogenases and is closest to the propylmalonyl-ACP desaturase TcsD, which catalyses the desaturation of a propyl group to an allyl group in the side-chain biosynthesis of FK506^35^,^36^,^37^ (Fig. 6). Because functional analyses for such terminal dehydrogenases have been limited exclusively to FK506 and its related metabolites^35^,^38^,^39^, we next expressed HliR in *Escherichia coli* as a polyhistidine-tagged protein to analyse its biochemical features (Supplementary Fig. S19). With the full-length dihydro analogue **4** in hand, we first examined the possibility of HliR-mediated dehydrogenation being the ultimate step in the biosynthesis of haliangicin. The lack of conversion of **4** into **1** by HliR *in vitro* precluded this hypothesis (Supplementary Fig. S20). Next, we examined the possibility that formation of the terminal alkene might occur in the starter diketide **2**. To prove this, a thio ester mimic of **2**, *S*-(2-methylpent-2-enoyl)-*N*-acetylcysteamine (**2a**), was synthesized as the proposed substrate. The recombinant HliR converted **2a** entirely into the corresponding dehydro product **3a** (Fig. 7a). Preparation of **3a** by feeding **2a** to the HliR-expressing *E. coli* yielded a sufficient amount of **3a** to permit comprehensive spectroscopic analysis (Supplementary Methods). The substrate specificity was then evaluated by using 14 additional short thio ester mimics, revealing that HliR is highly specific to α,β-unsaturated short acyl compounds, but does not consume saturated or γ,δ-unsaturated compounds (Fig. 7b and Supplementary Fig. S20). Among the substrates tested, *S*-(pent-2-enoyl)-*N*-acetylcysteamine (**2b**), like **2a**, was also completely converted into **3b**, whereas *S*-(hex-2-enoyl)-*N*-acetylcysteamine (**2c**) and *S*-(4-methylpent-2-enoyl)-*N*-acetylcysteamine (**2d**) underwent partial dehydrogenation. These results suggested that the presence of substituents at the γ- or δ-position might hinder the dehydrogenation reaction from the enzyme. In addition, unlike TcsD, which demands acyl-carrier protein-tethered substrates, HliR can utilize free small thio esters that mimic acyl-CoA substrates, offering a new strategy for preparing terminal alkenes.

### Biological activities of haliangicin analogues

Haliangicin (**1**) is known to show potent inhibitory activity against the plant pathogenic oomycete *Phytophthora capsici*; in this activity, the terminal β-methoxyacrylate moiety serves as the pharmacophore^12^ Because the unnatural analogues **4** and **7** obtained in this study are modified at the terminus opposite the β-methoxyacrylate, additional biological information might be obtained. In a test using *P. capsici*, analogue **4**, which is saturated at the 14,15-olefin, showed the same activity as **1** and the versatile fungicide metalaxyl, whereas analogue **7**, lacking the 12,13-epoxide moiety, was 30-fold less active than the other analogues (Table 2), suggesting that the epoxide plays a key role in this activity. It is noteworthy that these compounds showed potent cytotoxicity against a tumour cell line and that analogue **7** was more active than the natural compound **1**. These compounds showed no inhibitory effects on other microorganisms tested.

## Discussion

Since the isolation in 2002 of *Haliangium ochraceum* as the first marine myxobacterium, there have been few additional examples of this group. Although such a rare group of microorganisms might appear to be inconsequential, their secondary metabolites, once isolated, were found to be chemically and biologically novel. In addition, a report on Type I PKS genes of marine myxobacterial isolates suggested that the genes in marine myxobacteria were much more unusual than those in terrestrial strains. One of the greatest difficulties in studying marine myxobacteria is their fastidious nature (slow growth, low secondary metabolism, cell aggregation, etc.), which also makes it difficult to apply recently developed techniques for genetic manipulation. Therefore, the present work provides the first insights into biosynthesis in marine myxobacteria. The successful heterologous expression of the biosynthetic machinery for the PKS-type metabolite haliangicin (**1**) provided several benefits that would be difficult to achieve with the native producer, especially the improved productivity of the fungicide and the production of unnatural analogues, one of which was a more potent cytotoxin than the natural **1**. The easier access to the biosynthetic machinery of **1** in a different organism also led to the functional characterization of a unique dehydrogenase that introduces the terminal alkene moiety of **1**, thereby widening our understanding of the biosynthetic mechanism underlying the formation of terminal alkene groups. Such γ,δ-dehydrogenation reactions hold promise for generating novel structures *in vitro* in the future. Genomic information on the native antibiotic producer *H. ochraceum*, which suggests the presence of six other latent biosynthetic gene clusters, prompts us to analyse them by applying our present expertise to produce novel metabolites that are not obtained by cultivation of the native marine myxobacterium.

## Methods

### Bacterial strains, plasmids, primers and culture conditions

All bacterial strains, vectors, and oligonucleotides (STAR Oligo; Rikaken Co., Ltd., Nagoya) used in this study are listed in Supplementary Tables S4–S6. The marine myxobacterium *Haliangium ochraceum* SMP-2 was cultivated as previously described^11^. CTT medium^40^ was used for the preculture of *Myxococcus xanthus* ATCC25232 (wild type) and all its mutants at 30 °C. Production medium^41^ supplemented with 2–4% (w/v) Sepabeads SP207 resin (Mitsubishi Chemical Co., Tokyo) and a 0.1% (v/v) trace-elements solution (TES)^42^ was used for the heterologous production of haliangicin (**1**) and its related metabolites.

### Construction and screening of a genomic cosmid library of *Haliangium ochraceum* SMP-2

The chromosomal DNA of *H. ochraceum* was prepared as previously described^43^. The chromosomal DNA was digested with *Sau*3AI (3 min at 37 °C) and then separated on 0.5% Certified™ Low Melt Agarose gel (Bio-Rad Laboratories, Berkeley, CA) by gel electrophoresis (20 V, overnight). The agarose gel containing DNA fragments of around 30–45 kb in size was melted at 68 °C for 10 min and subsequently digested with GELase™ (Epicentre, Madison, WI) at 45 °C for 1.5 h. The genomic DNA fragments were ligated into the *Bam*HI-digested *E. coli/Streptomyces* shuttle vector pDW103, and the ligation mixture was packed using the Gigapack III Gold Packaging Extract kit (Stratagene California, San Diego, CA) to obtain about 530 μL of packaged phages, 25 μL of which were transferred into *E. coli* XL 1-Blue. Single colonies were transferred into eighteen 96-well microplates in LB medium.

A PCR amplification was carried out with the degenerate primers KS1UP/KSD1^44^ by using the *H. ochraceum* genomic DNA as a template to obtain two KS fragments HKS1 and HKS2 (Supplementary Fig. S1). The genomic cosmid library containing about 1700 cosmids was then screened for PKS genes by colony hybridization with the above-mentioned KS probes HKS1 and HKS2. Five cosmids, c3-5F, c4-6B, c7-6E, c9-4F, and c10-11C, containing KS domain(s) were obtained (Supplementary Fig. S2). Each cosmid was end-sequenced (Supplementary Table S7) by forward-primer DWF1 and reverse-primer DWR. Of these five cosmids, c7-6E and c10-11C were subjected to shotgun sequencing (Takara Bio Inc., Tokyo). Two discrete contigs of 33,197 bp and 22,829 bp were obtained and the remaining 7,420 bp were further sequenced by the genome-walking approach. One continuous region of 63,446 bp was finally assembled and subjected to sequence analysis.

### Analysis of the haliangicin biosynthetic gene cluster (*hli*)

The obtained continuous contig of 63.4 kbp was annotated by various sequence-analysis tools, including BLAST, CDD,^45^ and antiSMASH analysis^46^. The multiple alignments of amino acid sequences were generated by using the Clustal Omega program (http://www.ebi.ac.uk/Tools/msa/clustalo/) provided by the European Molecular Biology Institute (Hinxton, UK) and neighbour-joining trees of proteins were generated by Geneious Tree Builder. KS domains in *hli* were extracted and analysed by using the NaPDoS Web tool (http://napdos.ucsd.edu/)^47^.

### Heterologous expression of the *hli* gene cluster in *M. xanthus*

Cosmids c7-6E and c10-11C were modified through λ-Red recombineering. All techniques pertaining to the λ-Red recombineering are described in the published protocol^22^. For detailed modification procedures, refer to the Supplementary Methods and Figs S3 and S4. The resulting cosmids c7-6E TA-Kan^R^ and c10-11C Tet^R^ were introduced into the chromosome of *M. xanthus* ATCC25232 in a stepwise manner (Supplementary Fig. S5). Namely, the first cosmid c7-6E TA-Kan^R^ was integrated into the genome of *M. xanthus* through the TA fragment that is a 1.8 kbp internal fragment homologous to *ta-1* responsible for the myxovirescin A biosynthesis^48^. The second cosmid c10-11C Tet^R^ was then introduced through the 14 kbp region shared with the first cosmid. Electrocompetent cells of *M. xanthus* were prepared according to the reported methods^21^,^49^. In the first stage, 1.5 μg of c7-6E TA-Kan^R^ was added to 100 μL of a chilled cell suspension of *M. xanthus* ATCC25232 and electroporated (Gene Pulser^TM^, BIO-RAD) at 25 μF, 1.8 kV, and 200 Ω in 0.2 cm electroporation cuvettes. The kanamycin-resistant colonies (named as *M. xanthus* c7-6E TA-Kan^R^) appeared after seven days and were verified by colony PCR using specific primers to detect the integration of c7-6E TA-Kan^R^ into the chromosome of *M. xanthus* ATCC25232 (Supplementary Fig. S6a). The expected homologous recombination of the TA fragment was also confirmed by PCR (Supplementary Fig. S6b). Subsequently, 1.7 μg of the second modified cosmid c10-11C Tet^R^ was electroporated into *M. xanthus* c7-6E TA-Kan^R^ under the same conditions as those for c7-6E TA-Kan^R^ through the 14 kbp region shared with cosmid c7-6E. Mutants were selected on CTT agar plate (oxytetracycline 12.5 μg/mL) for one week and checked by colony PCR using specific primers (Supplementary Fig. S7). The obtained construct carrying the entire length of *hli* was named *M. xanthus* c10-11C/c7-6E. The confirmation of the heterologous production of haliangicin (**1**) in *M. xanthus* c10-11C/c7-6E is described in the Supplementary Methods.

### Feeding experiments with stable-isotope-labelled precursors

The heterologous host *M. xanthus* c10-11C/c7-6E was cultivated in three or four 100 mL aliquots of production medium supplemented with 2% (w/v) absorbent resin (Sepabeads SP207) and 0.1% TES. Filter-sterilized 0.2 M solutions of the isotope-labelled compounds [2-^13^C]acetate, [1, 2-^13^C_2_]acetate, [1-^13^C]propionate, _L_-[*methyl*-^13^C]methionine, [1-^13^C]glycolic acid, and [U-^13^C_3_]glycerol in water were separately added in two portions to the bacterial culture after 48 hours and 72 hours, respectively, to give a final concentration of 4 mM. After cultivation for six or seven days in total at 30 °C and 180 rpm, labelled haliangicin was extracted, purified, and analysed by ^13^C NMR spectroscopy (Supplementary Fig. S9).

### Optimization of haliangicin production in the *M. xanthus* heterologous host

The heterologous host *M. xanthus* c10-11C/c7-6E was cultured in the production medium. 1 M Aqueous solutions of sodium acetate, sodium propionate, and glycerol were prepared, filter-sterilized, and added to the autoclaved culture medium. l-Methionine was added before autoclaving. For higher concentrations of sodium acetate (100 mM or 200 mM), an autoclaved 5 M stock solution was added in two portions to the medium on the second and third day, respectively, to avoid inhibition of the growth of *M. xanthus* in its early phase. After cultivation at 30 °C and 180 rpm for the scheduled time, the resins and cell pellets were separated from the medium by centrifugation (6000 rpm, 10 min) and extracted twice with acetone. The crude extracts were filtered, evaporated to dryness, and subjected to HPLC analysis [Develosil ODS-UG-5 (ϕ 4.6 × 250 mm), λ = 290 nm; 75% MeOH–H_2_O, flow rate: 1 mL/min]. The results are summarized in Supplementary Fig. 4b.

### Inactivation of haliangicin biosynthetic genes in *M. xanthus* c10-11C/c7-6E

Disruption of the *hli* genes was accomplished by integration of a disruption vector into the genome of the heterologous host *M. xanthus* c10-11C/c7-6E through single crossover recombination. Plasmid pHSG398 (Takara Bio. Co., Ltd, Tokyo) was first linearized by inverse PCR using primers pHSG398 Inverse-F/pHSG398 Inverse-R; by infusion, the original chloramphenicol-resistance gene was replaced with the Tn5 promoter and a nourseothricin-resistance gene (*Nrs*^*R*^) (amplified from pNR1^50^ with primers Kan Nrs-f/Amp Nrs-r), resulting a plasmid named pHSG398 Infu Nrs^R^. The internal fragments of the target genes *hliD*, *hliR*, and *hliU* were amplified from *H. ochraceum* genomic DNA with primers ORF4-MT-f1/ORF4-MT-r1, pHSG18ACAD Infu-f/pHSG18ACAD Infu-r, and pHSG 21Ox Infu F/pHSG 21Ox Infu R, respectively, and inserted into pHSG398 Infu Nrs^R^. Each disruption vector (1.0–1.5 μg) was electroporated into the haliangicin-producing host *M. xanthus* c10-11/7-6E at 25 μF, 1.8 kV, and 200 Ω in 0.2 cm electroporation cuvettes. Transformants were selected on CTT agar plate (nourseothricin: 100 μg/mL) for about 5–7 days and then grown in 1 mL of CTT medium (kanamycin 200 μg/mL, oxytetracycline 12.5 μg/mL, nourseothricin 100 μg/mL) for an additional three days. The cell culture was directly subjected to PCR to detect integration of the corresponding plasmid into the genome (Supplementary Fig. S15, S16, S18). For the isolation of the unnatural analogues, please refer to the Supplementary Methods.

### Cloning and expression of HliR (acyl-CoA dehydrogenase)

HliR was amplified from the *H. ochraceum* genome by PCR using high-fidelity PrimeSTAR Max DNA polymerase (Takara Bio Inc.) and the primers HliR Exp Infu f/HliR Exp Infu r, and subsequently cloned into *BamH*I/*Xho*I double-digested pET32a by using an In-Fusion^®^ HD Cloning Kit (Clontech Laboratories, Inc., Mountain View, CA). The purified plasmid was transformed into Single Step (KRX) Competent Cells (Promega, Madison, WI). For the expression of HliR, when the OD_600_ reached 0.6, a final concentration of 0.1% rhamnose and 0.4 mM IPTG were added to induce protein expression. After induction for additional 20 h at 20 °C, the cells were harvested by centrifugation (6000 rpm, 5 min) and resuspended in 3 mL of extraction buffer (50 mM Tris-HCl, 0.4 M NaCl, pH 7.8). The resulting suspension was lysed by using a sonication homogenizer (50 W, five cycles) in the presence of benzonase nuclease (Novagen, Madison, WI) over ice for 30 min. The lysate was centrifuged (6000 rpm, 5 min) to remove insoluble cell debris. The crude protein extracts were stored at −30 °C for subsequent purification. A MagneHis^TM^ Protein Purification System (Promega, Madison, WI) was used to purify the His_6_-tagged protein. The crude extract and purified HliR were analysed by SDS-PAGE (10% Tris-HCl gel) (Supplementary Fig. S19).

### *In vitro* enzymatic characterization of HliR

A typical HliR assay contained 500 μM flavin adenine dinucleotide (FAD), 500 μM substrate (for chemical synthesis, see Supplementary Methods), and 1 μM recombinant HliR, in a pH 7.5 buffer (8 mM MgSO_4_, 10 mM Tris-HCl, and 1 mM potassium phosphate; total volume: 100 μL). The assay mixture was incubated at 30 °C overnight and then extracted with EtOAc. The extracts were analysed by HPLC under the conditions indicated in Supplementary Fig. S20.

## Additional Information

**How to cite this article**: Sun, Y. *et al.* Heterologous Production of the Marine Myxobacterial Antibiotic Haliangicin and Its Unnatural Analogues Generated by Engineering of the Biochemical Pathway. *Sci. Rep.* **6**, 22091; doi: 10.1038/srep22091 (2016).

## Supplementary Material

Supplementary Information

## Figures and Tables

**Figure 1:**
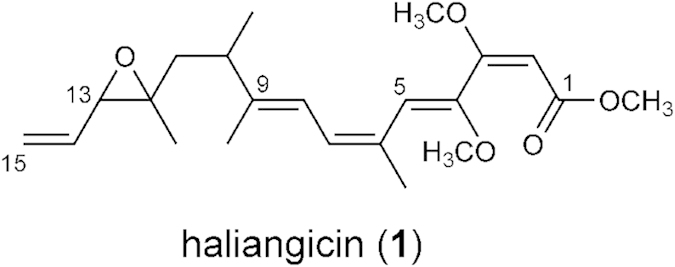
Structure of haliangicin.

**Table 1:** Proposed functions of the proteins involved in haliangicin biosynthesis.

Protein	Size (aa)	Proposed function	Top-hit homologues (Origin)	Similarity/ identity (%)
HliA	608	ABC transporter	ABC transporter protein (*Sorangium cellulosum*), CCE88383	58/34
HliB	609	ABC transporter	ABC transporter protein (*Sorangium cellulosum*), CCE88383	44/24
HliC	259	enoyl-CoA hydratase	enoyl-CoA hydratase (*Nostoc punctiforme* PCC 73102), YP_001866568	74/53
HliD	217	*O*-methyltransferase	JerF (*Sorangium cellulosum*), ABK32292	62/45
HliE	303	metallo-β-lactamase type TE	β-lactamase (*Rhodothermus marinus* SG0.5JP17-172), YP_004824011	67/49
HliF	1372	PKS (KS-AT-*O*-MT-ACP)	MtaF (*Stigmatella aurantiaca* DW4/3-1), YP_003953631	62/45
HliG	1966	PKS (KS-AT-DH-KR-ACP)	MxaD (*Stigmatella aurantiaca*), AAK57188	57/42
HliH	367	FkbH-like protein	HAD superfamily phosphatase (*Sorangium cellulosum*), CCE88385	78/62
HliI	387	acyl-CoA dehydrogenase	acyl-CoA dehydrogenase (*Sorangium cellulosum*), CCE88384	73/57
HliJ	83	ACP	hypothetical protein (*Paenibacillus dendritiformis*), WP_006675272	76/57
HliK	287	3-hydroxyacyl-CoA dehydrogenase	hydroxyacyl-CoA dehydrogenase (*Sorangium cellulosum*), CCE88386	79/66
HliL	950	PKS (KS-AT)	polyketide synthase (*Chondromyces crocatus*), CBD77738	61/48
HliM	407	HMG-CoA synthase	HMG-CoA synthase (*Nostoc punctiforme* PCC 73102), YP_001866566	65/48
HliN	83	ACP	acyl carrier protein (*Nostoc* sp. 'Peltigera membranacea cyanobiont'), AGH69805	81/56
HliO	277	enoyl-CoA hydratase	enoyl-CoA hydratase/isomerase (*Nostoc punctiforme* PCC 73102), YP_001866567	58/42
HliP	2890	PKS (KS-ACP-KS-AT- DH-KR-ACP)	polyketide synthase (*Myxococcus xanthus* DK 1622), YP_632696	55/42
HliQ	861	AT-ACP-*O*-MT	putative methoxymalonyl-CoA synthase (*Sorangium cellulosum*), AAK19884	59/44
HliR	392	acyl-CoA dehydrogenase	acyl-CoA dehydrogenase (*Herbidospora cretacea*), WP_030450088	69/55
HliS	626	AT-ACP	MxaF (*Stigmatella aurantiaca*), AAK57190	57/43
HliT	2225	PKS (KS-AT-DH-ER- KR-ACP)	polyketide synthase (*Sorangium cellulosum*), CCE88378	61/44
HliU	463	epoxidase	FAD-binding monooxygenase (*Geodermatophilus obscurus* DSM 43160), YP_003408356	56/42

**Table 2:** Biological evaluation of haliangicin (1) and its analogues (4 and 7) obtained through gene disruption.

Organisms	1	4	7	PC
*Phytophthora capsici* (μg disk^–1^)	0.1	0.1	3.0	0.1
*Candida rugosa* (MIC, μg mL^–1^)	>32	>32	>32	0.13
*Bacillus subtilis* (MIC, μg mL^–1^)	>32	>32	>32	0.08
*Escherichia coli* (MIC, μg mL^–1^)	>32	>32	>32	8.0
HeLa S_3_ cells (IC_50_, nM)	41	55	17	8.6

Growth inhibition of the oomycete *P. capsici* was evaluated by a disk diffusion test and denoted as minimum doses to form a definite inhibition zone. Metalaxyl was used as a positive control (PC). In MIC test, PCs were amphotericin B for *C. rugosa* and ampicillin for *B. subtilis* and *E. coli*. The cytotoxicity against HeLa cells was determined by MTT test and paclitaxel was used as PC.
