# Supplementary material for: Heterologous Production of the Marine Myxobacterial Antibiotic Haliangicin and Its Unnatural Analogues Generated by Engineering of the Biochemical Pathway
Source: Sci Rep. 2016 Feb 26;6:22091. doi: 10.1038/srep22091 (PMC4768178; doi:10.1038/srep22091)
Supplement: Supplementary Information [file srep22091-s1.pdf]

Supplementary Information

Heterologous Production of the Marine Myxobacterial Antibiotic Haliangicin and Its Unnatural Analogues Generated by Engineering of the Biochemical Pathway

Yuwei Sun[1], Zhiyang Feng[1,†], Tomohiko Tomura[1], Akira Suzuki[1], Seishi Miyano[1], Takashi Tsuge[1], Hitoshi Mori[1], Joo-Won Suh[2], Takashi Iizuka[3], Ryosuke Fudou[4], and Makoto Ojika[1,*]

[1] Graduate School of Bioagricultural Sciences, Nagoya University, Furo-cho, Chikusa-ku, Nagoya 464-8601, Japan
[2] Department of Biological Sciences, Myongji University, Yongin, Gyunggido 449-728, Korea
[3] Institute for Innovation, Ajinomoto Co., Inc., Kawasaki, Kanagawa 210-8681, Japan
[4] R&D Planning Department, Ajinomoto Co., Inc., Chuo-ku, Tokyo 104-8315, Japan

* Corresponding author
Tel.: (+81) 52-789-4116. E-mail: ojika@agr.nagoya-u.ac.jp
[†] Present address: College of Food Science and Technology, Nanjing Agricultural University, Weigang, Nanjing 210095, China

Table of Contents

Supplementary Methods 4

λ-Red recombineering of cosmids c7-6E and c10-11C 4

Heterologous production of haliangicin in *M. xanthus* c10-11C/c7-6E. 4

Chemical synthesis of *S*-Acyl-*N*-acetylcysteamine (SNAC) derivatives. 4

Bioconversion of (*E*)-2-methylpent-2-enoyl SNAC (2a) and (*E*)-2-pentenoyl SNAC (2b). 5

Isolation of unnatural haliangicin analogs from gene-disrupted mutants of *M. xanthus.* 6

Methyl esterification of *M. xanthus* Δ*hliD* extracts. 7

Bioassay methods 7

Supplementary Figures 8

Figure S1. Sequences of HKS1 and HKS2 used for screening cosmid library. 8

Figure S2. Cosmids harboring *hli* genes. 9

Figure S3. Modification of c7-6E and c10-11C *via* λ-Red recombineering. 9

Figure S4. Restriction analysis and PCR examination of two modified cosmids. 10

Figure S5. Integration of c6-7E TA-Kan^R and c10-11C Tet^R into *M. xanthus*. 11

Figure S6. PCR confirmation of the integration of c7-6E TA-Kan^R into the chromosome of *M. xanthus*. 11

Figure S7. PCR confirmation of the integration of c10-11C Tet^R into the chromosome of *M. xanthus*. 12

Figure S8. Heterologous haliangicin production profile in *M. xanthus*. 12

Figure S9. Feeding experiments with labeled precursors. 13

Figure S10. Phylogenetic analysis of KS domains in *hli* genes 15

Figure S11. Proposed starter biosynthesis of haliangicin 16

Figure S12. The putative diketide synthases for the biosynthetic formation of the starters. 17

Figure S13. Proposed *β*-methyl branching biochemistry in haliangicin biosynthesis. 18

Figure S14. Proposed biosynthetic pathway of methoxymalony-ACP in haliangicin biosynthesis. 18

Figure S15. Inactivation of HliR (acyl-CoA dehydrogenase). 19

Figure S16. Inactivation of HliD (*O*-methyltransferase). 20

Figure S17. Methyl esterification of *M. xanthus* Δ*hliD* extracts using TMS diazomethane. 22

Figure S18. Inactivation of HliU (epoxidase). 23

Figure S19. Expression and purification of recombinant HliR. 24
Figure S20. *In vitro* enzymatic reaction of recombinant HliR. 24
Figure S21. Spectra of 14,15-dihydrohaliangicin (4). 27
Figure S22. Spectra of 12,13-deoxylhaliangicin (7). 30
Figure S23. NMR spectra of haliangicin (1). 33
Figure S24. NMR spectra of 2-methylpent-2,4-dienoyl SNAC (3a). 34
Figure S25. NMR spectra of pent-2,4-dienoyl SNAC (3b). 35
Figure S26. Biological activities 36
Supplementary Tables 37
Table S1. PKS in *hli* for the biosynthesis of haliangicin skeleton. 37
Table S2.*β*-methyl branching cassette in *hli* and its homology. 37
Table S3. Methoxymalony-ACP cassette in *hli* and its homology. 37
Table S4. Bacterial strains used in this study. 38
Table S5. Vectors used in this study. 39
Table S6. Primers used in this study. 40
Table S7. Analysis of end sequences of five cosmids that contain KS domains. 41
Table S8. NMR data for unnatural haliangicin analogs in $CDCl_3$. 41
References 42

Supplementary Methods

λ-Red recombineering of cosmids c7-6E and c10-11C

All techniques concerning λ-Red recombineering were described in the published protocol.[1] The gene cassette "TA-Kan^R" was first prepared by PCR with primers blaF-TAf/blaR-kan1 from plasmid pTA-Kan^R, which was constructed in advance by cloning of TA fragment (a 1.8-kbp internal fragment amplified with primers TAf/Tar from myxovirescin A biosynthetic gene *Ta-1* of *M. xanthus*[2]) into pGEM-T Easy vector (Promega, Madison, WI) followed by infusion of kanamycin resistance gene *Kan^R* [amplified with primers PstI-Kan1/SpeI-Kan2 from pCR 2.1 TOPO vector (Life Technologies, U.S.)] into the TA-containing vector. The ampicillin resistance gene (*Amp^R*) in cosmid c7-6E was then replaced with the cassette TA-Kan^R by λ-Red recombineering, resulting in the modified cosmid named c7-6E TA-Kan^R (Supplementary Fig. S3a). Cosmid c10-11C was also modified in a similar way, in which *Amp^R* was replaced by tetracycline resistance gene (*Tet^R*) [amplified with primers blaF-tetU/blaR-tetD from *E. coli* YM297 genome (NBRP, Japan)], generating the modified cosmid c10-11C Tet^R (Supplementary Fig. S3b). The two modified cosmids were confirmed by PCR and restriction analysis (Supplementary Fig. S4).

Heterologous production of haliangicin in *M. xanthus* c10-11C/c7-6E.

Four clones of *M. xanthus* c10-11C/c7-6E were randomly selected and cultivated in 50 mL CTT medium (oxytetracycline 12.5 μg/mL) supplemented with 2% (w/v) SEPABEADS SP207 absorber resin at 30 °C. The cells and resin were harvested from 4 days culture broth by centrifugation (6000 rpm, 5 min) and extracted with MeOH (50 mL × 2) at 30 °C for 30 min. After filtration, the combined filtrate were concentrated *in vacuo* and extracted with EtOAc (50 mL × 3). The EtOAc extracts were combined and washed with H_2O (30 mL) and dried to afford a yellow oil. The cultures of *M. xanthus* ATCC25232 (wild type) and *H. ochraceum* (native haliangicin producer) were extracted in the same way. A sample of 0.5 mL broth eq. from each extract was subjected to HPLC analysis [Develosil ODS-UG-5 column (ϕ 4.6 × 250 mm, Nomura Chemical Ltd.), linear gradient elution of 30% to 100% (40 min) methanol in H_2O, flow rate of 1 mL/min, detected at 290 nm]. HPLC profiles are shown in Fig. 3.

Chemical synthesis of *S*-Acyl-*N*-acetylcysteamine (SNAC) derivatives.

To a solution of 1-ethyl-3-(3-dimethylaminopropyl)carbodiimide hydrochloride (1.5 eq) and 4-dimethylaminopyridine (0.1 eq) in anhydrous dichloromethane was added the appropriate acids (1.2 ~ 1.5 eq) and *N*-acetylcysteamine (1 eq). The reaction mixture was stirred under nitrogen at room temperature overnight, then diluted with dichloromethane and washed with H_2O. The organic layer was dried, concentrated *in vacuo*, and purified by silica gel chromatography to afford the pure thioester products.

(*E*)-2-methyl-2-pentenoyl SNAC (2a): colorless oil, ^{1}H NMR ($CDCl_3$, 400 MHz) δ 6.73 (qt, *J* = 1.0, 7.3 Hz, 1H), 6.07 (brs, 1H), 3.42 (q, *J* = 6.1 Hz, 2H), 3.04 (t, *J* = 6.4 Hz, 2H), 2.23 (m, 2H), 1.95 (s, 3H), 1.85 (d, *J* = 1.0 Hz, 3H), 1.08 (t, *J* = 7.6 Hz, 3H). ESI-MS *m/z* 216.1 $[M+H]^+$, 238.1 $[M+Na]^+$.

(*E*)-2-pentenoyl SNAC (2b): colorless oil, ^{1}H NMR ($CDCl_3$, 400 MHz): δ 6.96 (dt, *J* =15.6, 6.4 Hz, 1H), 6.10 (d, *J* = 15.6 Hz, 1H), 6.08 (br, 1H), 3.42 (q, *J* = 6.0 Hz, 2H), 3.07 (t, *J* = 6.4 Hz, 2H), 2.22 (m, 1H), 1.94 (s, 3H), 1.06 (t, *J* = 7.2 Hz, 3H).

propionyl SNAC: colorless oil, ^{1}H NMR ($CDCl_3$, 400 MHz) δ 5.99 (br, 1H), 3.41 (q, *J* = 6.2 Hz, 2H), 3.01 (t, *J* = 6.5 Hz, 2H), 2.58 (q, *J* =7.5 Hz, 2H), 1.95 (s, 3H), 1.17 (t, *J* = 7.4 Hz, 3H).

acryloyl SNAC: colorless oil, ^{1}H NMR ($CDCl_3$, 400 MHz) δ 6.34 (m, 2H), 6.01 (br, 1H), 5.72 (dd, *J* = 9.6, 1.6 Hz, 1H), 3.46 (q, *J* = 6.2 Hz, 2H), 3.12 (t, *J* = 6.4 Hz, 2H), 1.96 (s, 3H).

butyryl SNAC: colorless oil, ^{1}H NMR ($CDCl_3$, 400 MHz): δ 6.18 (br, 1H), 3.38 (q, *J* = 6.4 Hz, 2H), 2.98 (t, *J* = 6.4 Hz, 2H), 2.51 (t, *J* = 7.6 Hz, 2H), 1.92 (s, 3H), 1.65 (m, 2H), 0.91 (t, *J* =7.6 Hz, 3H).

valeryl SNAC: colorless oil, ^{1}H NMR ($CDCl_3$, 400 MHz): δ 6.02 (br, 1H), 3.40 (q, *J* = 6.0 Hz, 2H), 3.00 (t, *J* = 6.4 Hz, 2H), 2.55 (t, *J* = 7.6 Hz, 2H), 1.94 (s, 3H), 1.62 (m, 2H), 1.33 (m, 2H), 0.89 (t, *J* = 7.2 Hz, 3H).

hexanoyl SNAC: colorless oil, ^{1}H NMR ($CDCl_3$, 400 MHz): δ 6.17 (br, 1H), 3.39 (q, *J* = 6.0 Hz, 2H), 2.98 (t, *J* = 6.4 Hz, 2H), 2.53 (t, *J* = 7.6 Hz, 2H), 1.93 (s, 3H), 1.62 (m, 2H), 1.27 (m, 4H), 0.85 (t, *J* = 6.8 Hz, 3H).

heptanoyl SNAC: colorless oil, ^{1}H NMR ($CDCl_3$, 400 MHz): δ 6.29 (br, 1H), 3.36 (q, *J* = 6.4 Hz, 2H), 2.97 (t, *J* = 6.4 Hz, 2H), 2.51 (t, *J* = 7.2 Hz, 2H), 1.92 (s, 3H), 1.59 (m, 2H), 1.25 (m, 6H), 0.82 (t, *J* = 6.8 Hz, 3H).

2-methylvaleryl SNAC: colorless oil, ^{1}H NMR ($CDCl_3$, 400 MHz): δ 5.90 (br, 1H), 3.42 (q, *J* = 6.0 Hz, 2H), 3.00 (t, *J* = 6.4 Hz, 2H), 2.66 (m, 1H), 1.95 (s, 3H), 1.68 (m, 1H), 1.37 (m, 3H), 1.16 (d, *J* = 6.8 Hz, 3H), 0.89 (t, *J* = 7.2 Hz, 3H).

4-methylvaleryl SNAC: colorless oil, ^{1}H NMR ($CDCl_3$, 400 MHz): δ 6.17 (br, 1H), 3.38 (q, *J* = 6.4 Hz, 2H), 2.98 (t, *J* = 6.4 Hz, 2H), 2.53 (t, *J* = 7.6 Hz, 2H), 1.93 (s, 3H), 1.52 (m, 3H), 0.86 (d, *J* = 6.4 Hz, 6H).

2-hexenoyl SNAC: colorless oil, ^{1}H NMR ($CDCl_3$, 400 MHz): δ 6.89 (dt, *J* =15.6, 7.2 Hz, 1H), 6.16 (br, 1H), 6.10 (d, *J* = 15.2 Hz), 3.43 (q, *J* = 6.0 Hz, 2H), 3.06 (t, *J* = 6.4 Hz, 2H), 2.16 (q, *J* = 7.2, 2H), 1.94 (s, 3H), 1.47 (m, 2H), 0.91 (t, *J* = 7.2 Hz, 3H).

4-methyl-2-pentenoyl SNAC: colorless oil, ^{1}H NMR ($CDCl_3$, 400 MHz): δ 6.87 (dd, *J* =15.6, 6.8 Hz, 1H), 6.16 (br, 1H), 6.04 (d, *J* = 15.6 Hz, 1H), 3.42 (q, *J* = 6.4 Hz, 2H), 3.06 (t, *J* = 6.4 Hz, 2H), 2.44 (m, 1H), 1.94 (s, 3H), 1.05 (d, *J* = 6.8 Hz, 6H).

cyclopent-1-enoyl SNAC: white needles, ^{1}H NMR ($CDCl_3$, 400 MHz): δ 6.81 (m, 1H), 6.24 (br, 1H), 3.40 (q, *J* = 6.4 Hz, 2H), 3.04 (t, *J* = 6.4 Hz, 2H), 2.53 (m, 4H), 1.94 (m, 2H), 1.93 (s, 3H).

cyclohex-1-enoyl SNAC: white needles, ^{1}H NMR ($CDCl_3$, 400 MHz): δ 6.98 (m, 1H), 6.11 (br, 1H), 3.41 (q, *J* = 6.4 Hz, 2H), 3.03 (t, *J* = 6.4 Hz, 2H), 2.23 (m, 4H), 1.93 (s, 3H) , 1.61 (m, 4H).

2-methyl-4-pentenoyl SNAC: Colorless oil, ^{1}H NMR ($CDCl_3$, 400 MHz): δ 5.91 (br, 1H), 5.70 (m, 1H), 5.05 (m, 2H), 3.41 (q, *J* = 6.4 Hz, 2H), 3.01 (t, *J* = 6.4 Hz, 2H), 2.73 (q, *J* = 6.8 Hz, 1H), 2.44 (m, 1H), 2.17 (m, 1H), 1.94 (s, 3H), 1.17 (d, *J* =6.8 Hz, 3H)

4-pentenoyl SNAC: Colorless oil, ^{1}H NMR ($CDCl_3$, 400 MHz): δ 6.09 (br, 1H), 5.76 (m, 1H), 5.02 (m, 2H), 3.39 (q, *J* = 6.0 Hz, 2H), 3.00 (t, *J* = 6.4 Hz, 2H), 2.65 (t, *J* = 7.6 Hz, 2H), 2.38 (dt, *J* =7.2, 6.6 Hz, 2H), 1.94 (s, 3H)

Bioconversion of (*E*)-2-methylpent-2-enoyl SNAC (2a) and (*E*)-2-pentenoyl SNAC (2b).

The HliR-overexpressing host *E. coli* KRX pET32a-HliR was cultured in five 50 mL of LB mediums for 2 days. To each 50 mL culture, (*E*)-2-methylpent-2-enoyl SNAC (**2a**, 2.7 mg in 125 μL EtOH) or (*E*)-2-pentenoyl SNAC (**2b**, 5 mg in 125 μL EtOH) was added twice at 6 h and 16 h after the induction was started. When the second portion was added, the cultures were

transferred to 30 °C and incubated for additional 24 h. The cultures were centrifuged (6000 rpm, 5 min) and cell pellets were extracted with methanol (30 mL × 2) and the supernatants were extracted with EtOAc (50 mL × 3). The methanol extracts and EtOAc layer were combined and concentrated. The resulting residue was further subjected to silica gel column chromatography [5 g of silica gel, eluted with hexane/EtOAc (1/1, v/v)] to yield **3a** (2-methylpent-2,4-dienoyl SNAC, 2.6 mg): colorless oil; UV (MeOH) λ_{max} 257 (ε 17,000), 205 (ε 11,000), 282 (sh, ε 12,000)nm; IR (film) ν_{max} 3289, 2922, 1654, 1542, 1457, 1376, 1288, 983 cm^{-1}; ^1H NMR ($CDCl_3$, 400 MHz) δ 7.15 (d, *J* = 11.2 Hz, 1H), 6.68 (ddd, *J* =10.0, 10.8, 16.4 Hz, 1H), 5.88 (brs, 1H), 5.65 (d, *J* = 16.4 Hz, 1H), 5.55 (d, *J* =10.0 Hz, 1H), 3.47 (q, *J* = 6.4 Hz, 2H), 3.10 (t, *J* = 6.4 Hz, 2H), 2.00 (d, *J* = 1.2 Hz, 3H), 1.97 (s, 3H); ^{13}C NMR ($CDCl_3$, 100 MHz): δ 193.9, 170.3, 137.5, 135.3, 131.8, 125.8, 39.8, 28.5, 23.3, 12.7; HRMS calcd. for $C_{10}H_{15}NO_2NaS$ 236.0716; found *m/z* 236.0720 $[M+Na]^+$. **3b** (pent-2,4-dienoyl SNAC, 4.1 mg): colorless oil; UV (MeOH) λ_{max} 262 (ε 22,000), 204 (ε 9,600) nm; IR (film) ν_{max} 3288, 3081, 2930, 1656, 1591, 1550, 1289, 1254, 1181, 1121, 1032, 811 cm^{-1}; ^1H NMR ($CDCl_3$, 400 MHz): δ 7.21 (dd, *J* = 15.2, 10.8 Hz, 1H), 6.43 (ddd, *J* =16.8, 10.4, 10.4 Hz, 1H), 6.20 (d, *J* = 15.2 Hz, 1H), 5.92 (br, 1H), 5.70 (d, *J* = 17.2 Hz, 1H), 5.60 (d, *J* =9.6 Hz, 1H), 3.47 (dt, *J* = 6.4, 6.0 Hz, 2H), 3.12 (t, *J* = 6.4 Hz, 2H), 1.96 (s, 3H); ^{13}C NMR ($CDCl_3$, 100 MHz): δ 190.4, 170.3, 141.3, 134.4, 128.5, 127.6, 39.8, 28.4, 23.2; HRMS calcd. for $C_9H_{13}NO_2NaS$ 222.0559; found *m/z* 222.0563 $[M+Na]^+$.

Isolation of unnatural haliangicin analogs from gene-disrupted mutants of *M. xanthus*.

A gene-disrupted mutant *M. xanthus* Δ*hliR* or *M. xanthus* Δ*hliU* was cultured in 3 L or 1 L of production medium supplemented with 4% (w/v) SEPABEADS SP207 absorber resin, 0.1% trace elements solution and 200 mM sodium acetate at 30 °C in a similar way to *M. xanthus* c10-11C/c7-6E. An extract (646 mg) obtained from the cultures (3 L in total) of *M. xanthus* Δ*hliR* was separated by flash column chromatography [Hi-flash L size (Yamazen, Kyoyo, Japan), flow rate 12 mL/min, linear gradient of 0% → 20% EtOAc in hexane in 40 min] using a gradient system equipped with a Pump Module C-605 and a Pump Manager C-615 (BÜCHI, Switzerland). The fraction (99.3 mg) eluted at 27~40 min was subjected to preparative HPLC [Develosil ODS-UG-5 (ϕ 20 × 250 mm), Nomura Chemical Ltd.; detected at 340 nm, 75% MeOH in water, 10 mL/min] to yield 14,15-dihydrohaliangicin (**4**, 16.4 mg, t_R 42~46 min). An extract (358 mg) from the cultures (1 L in total) of *M. xanthus* Δ*hliU* was purified by flash column chromatography (Hi-flash M size, flow rate 6 mL/min, linear gradient of 0% → 15% EtOAc in hexane in 30 min). The fraction (22.3 mg) eluted at 18~26 min was subjected to preparative HPLC [Develosil ODS-UG-5 (ϕ 20 × 250 mm).; detected at 340 nm, 85% MeOH, 10 mL/min] to yield 12,13-deoxylhaliangicin (**7**, 6.4 mg, t_R 25.5~ 28 min). **14,15-dihydrohaliangicin (4)**: light yellow oil, $[\alpha]_D^{25}$ +27.4 (c 0.164, MeOH); UV (MeOH) λ_{max} 246 (ε 11,600) nm; IR (film) ν_{max} 1721, 1614, 1266, 1196, 1143, 1055, 977, 924, 897 and 824 cm^{-1}; HRMS calcd. for $C_{22}H_{34}O_5Na$: 401.2299; found *m/z* 401.2298 $[M+Na]^+$. For NMR data, see Supplementary Fig. S21 and Table S8. **12,13-deoxylhaliangicin (7)**: light yellow oil, $[\alpha]_D^{30}$ +43.1 (c 0.13, MeOH); UV (MeOH) λ_{max} 236 (ε 15,500) nm; IR (film) ν_{max} 1721, 1672, 1649, 1614, 1265, 1197, 1143, 1054, 977, 924, 827 and 755 cm^{-1}; HRMS calcd. for $C_{22}H_{32}O_4Na$ 383.2193; found *m/z* 383.2206 $[M+Na]^+$. For NMR spectroscopic data, see Supplementary Fig. S22 and Table S8.

Methyl esterification of *M. xanthus* Δ*hliD* extracts.

An ethyl acetate extract (121 mg) obtained from 500 mL of *M. xanthus* Δ*hliD* cultures was dissolved in Et_2O (5 mL) and 400 μL of trimethylsilyldiazomethane solution (2.0 M in Et_2O) was added. After stirring at room temperature for 30 min, the reaction mixture was dried under nitrogen flash and the residue was analyzed by HPLC (Develosil ODS-UG-5, ϕ 4.6 × 250 mm, 1 mL/min, detected at 290 nm, 20 → 100% methanol in H_2O in 40 min) (Supplementary Fig. S17). The mixture was chromatographed by the same method as that for haliangicin (**1**) and 12,13-deoxylhaliangicin (**7**) to yield haliangicin (**1**, 1.0 mg) and 12,13-deoxylhaliangicin (**7**, 0.7 mg).

Bioassay methods

(1) Anti-oomycete activity (See Supplementary Fig. S26a for results)

The phytopathogenic oomycete *Phytophthora capsici* NBRC 30696, purchased from NITE-Biological Resource Center (NBRC, Chiba, Japan), was cultured on a potato-agar medium [in 1 L, potato broth from 200 g of fresh potato, glucose (20 g), and agar (20 g)] in a 9-cm dish at 25 °C for 7 days in the dark. A piece of the colony was then inoculated on the center of a 5% V8 -agar medium [V8 vegetable juice (5 mL), agar (1.5 g), and water (95 mL)] in a 9-cm dish and incubated at 25 °C for 48 h in the dark until the colony grew to approximately 3–4 cm in diameter. A paper disc (6 mm in diameter) impregnated with a sample was placed 1 cm away from the front of the colony. After incubating for 22–24 h, the distance between the edge of the colony and the paper disc (control: 0 mm) was measured. The activity was defined as a minimum dose that induced a definite distance (0.5 mm or wider) between the colony and the disk.

(2) MIC assay

The microorganisms, *Candida rugosa* AJ 14513, *Bacillus subtilis* AJ 12865, and *Escherichia coli* AJ 3837, were provided by the Institute for Innovation, Ajinomoto Co., Inc. (Kanagawa, Japan). The protocols used for the assay are in the followings. For *B. subtilis* and *E. coli*, see *Methods for Dilution Antimicrobial Susceptibility Tests for Bacteria That Grow Aerobically; Approved Standard*—Ninth Edition. CLSI document M07-A9 [ISBN 1-56238-783-9], Clinical and Laboratory Standards Institute (Wayne, PA, USA), 2012. For *C. rugosa*, see *Reference Method for Broth Dilution Antifungal Susceptibility Testing of Yeasts; Approved Standard*—Second Edition. NCCLS document M27-A2 [ISBN 1-56238-469-4], NCCLS (Wayne, PA, USA), 2002.

(3) Cytotoxicity assay (See Supplementary Fig. S26b for results)

HeLa-S3 (SC) cells were provided by the RIKEN BRC through the National Bio-Resource Project of the MEXT, Japan. The cells were cultured in Eagle's minimal essential medium (EMEM) (Wako Pure Chemical Industries, Osaka, Japan) supplemented with 10% bovine serum, 100 units/mL penicillin, and 100 μg/mL streptomycin (Thermo Fisher Scientific Inc., MA, USA). A total of 10,000 cultured cells were seeded into each well of a 96-well plate containing 99 μL of the same medium. After pre-incubation for 24 h at 37 °C in an atmosphere of 5% CO_2, a compound in 1 μL of dimethyl sulfoxide (DMSO) was added to each well, and the cells were incubated an additional 48 h. A solution (10 μL) of 3-(4,5-dimethylthiazol-2-yl)-2,5-diphenyltetrazolium bromide (MTT) in phosphate buffer saline (PBS) (5 mg/mL) was then added to each well, and the plate was incubated for an additional 3 h. Subsequently, the medium was removed by aspiration, any generated formazan was dissolved in 100 μL of DMSO, and the absorbance was measured at 595 nm using a Multiskan FC microplate reader (Thermo Fisher Scientific).

Supplementary Figures

Figure S1. Sequences of HKS1 and HKS2 used for screening cosmid library.

>HKS1
GCTCGAGGTGGCGTGGGAGGCGCTCGAACACGCGGCCCTGCATCCCGACGAGATCTACGG
CAGCCAGACCGGCGTGTTCGTAGGCGCGTCCACCACCGACTACGCACAGCGGCAGATCGC
GTCCGGCACCGCGCCCTATCTCGACAAGTACTTCAGCACCGGCGCGGCCAACTGCTTCTGC
GCCGGCCGCGTCTCGTACACATTTGGCTTTCAGGGGCCCAGCGTGGTCCTCGACACCGCGT
GCTCGTCCTCGCTCGTCGCCGTTCACCTCGCCAGTCAAAGCCTGCGGCGCGGCGAATGCG
AGATGGCGCTCGCGGGCGGGGTCAACCTCATGTTGTCCCCGCTGCTCACCATCTTCACGTC
CAAACTGCAGACCATGGCGCCCGACGGCGCGTGCAAGACCTTCGACACGCGCGCCAACGG
CTTCGTGCGCGCCGAAGGCTGCGGCGTGGTGGTTCTCGAACGACTGTCCACCGCGCTGGC
CAAGGGCCACACGATCCATGCGCTCATCCGCGGCACGGCGATCAACCAAGATGGCCGCAGC
AACGGCCTCACCGCGCCCAACGTGGTCGCCCAGCAGCGGGTCATCGAGCGCGCGCTCGCC
AATGCCCACGTCAATCCCGCGCGTGTTACCTACGTCGAGACCCA

>HKS2
GTCGGGGGCCGCGACCTGGGCGCGCTCGAGCGCGCGGCCGATGACGTCCTGCTGGGCGA
TGCCGCTGGGGGCGGTGAGGCCGTTGGTGTAGCCGTCGTGGTTGAGCGCGGTGCCGCGG
ATGACGGCGGCCACGGGGTAGCCGGCGGCGATGGCGTCGGACAGCCGGGCGAGGAACAC
CATGCCGCAGCCCTCGGAGCGGACGTAGCCGTTAGCCGAGGCGTCGAAGGTCTTGCAGCG
GCCGTCGGGCGCCATCATACCGGCCTGGTTGAAGGCGATGGTCAGGTCGGGCGCGAGGAT
GGCGTTGACGCCACCGGCCAGGGCCAGGTCGCATTCGCCCTCGAGCAGGCTGCGGCAGG
CGAGGTGCACGGCCGCGAGCGACGACGAGCAGGCGGTGTCCACGGCCATGCTGGGGCCG
CGCAGGCCGAGCAGGTATGAGATGCGATTGGCCGCGATGCTGTGCGCGTTGCCGGTGCCG
GCGTGGCGGTCGAGGCGGGCGGGGTCGCCGAACTGGCGCTGCGCGTAGTCGCTGCTGCT
GATGCCGACGAAGACCCCGGTGCGCGAATCCTTGAGCGCCGCGGGCTGCTGCCCGGCGC
GTTCCAGGGTCTCCCACACGACCTCGAGGAGCATGCGCTG

Figure S2. Cosmids harboring *hli* genes.

The cosmids c3-5F, c4-6B, c7-6E, c9-4E, c10-11C overlap each other and cover a region of more than 80 kbp. Characteristic fragments for confirming these cosmid relationships were (1) PKS fragments (red, purple, and blue), (2) an acyl-CoA dehydrogenase fragment homologous to SorD (green), and (3) an HMG-CoA synthase fragment homologous to MupH (orange).

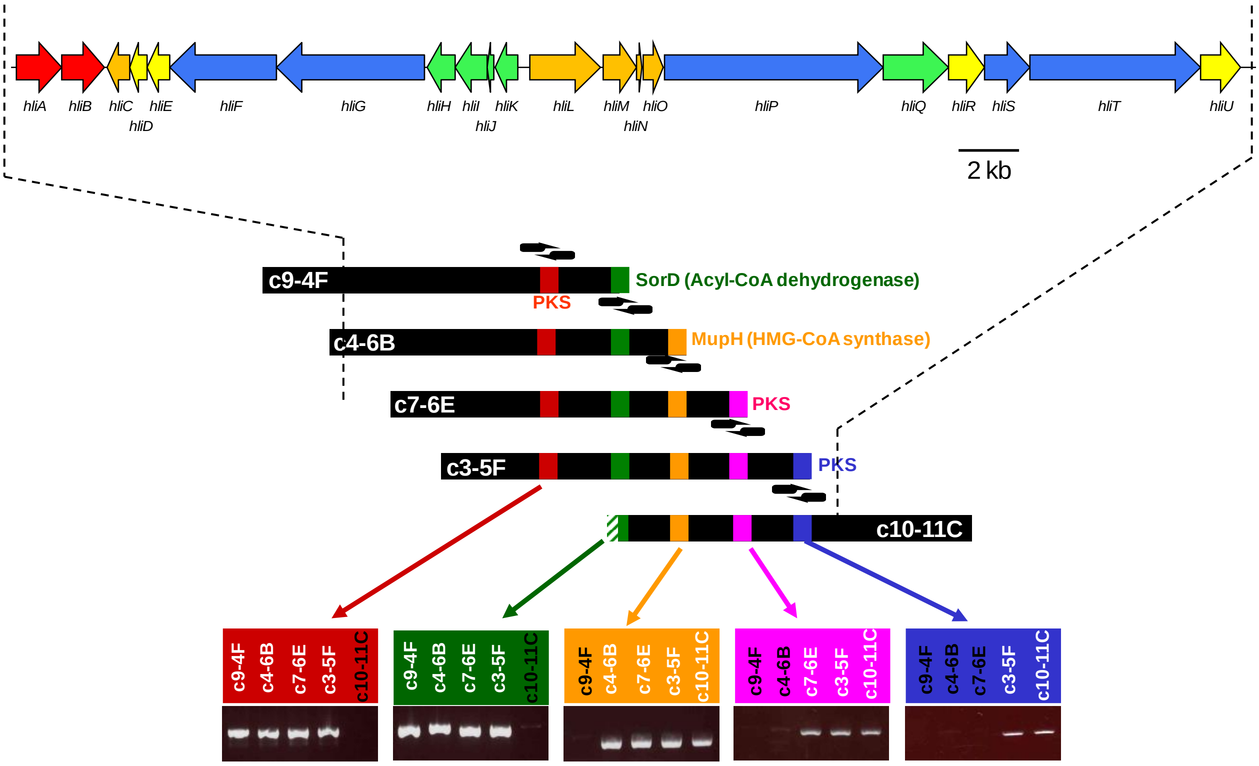

Figure S3. Modification of c7-6E and c10-11C *via* λ-Red recombineering.

a. Construction of c7-6E TA-Kan^R from c7-6E by replacement of Amp^R with TA-Kan^R

b. Construction of c10-11C Tet^R from c10-11C by replacement of Amp^R with Tet^R

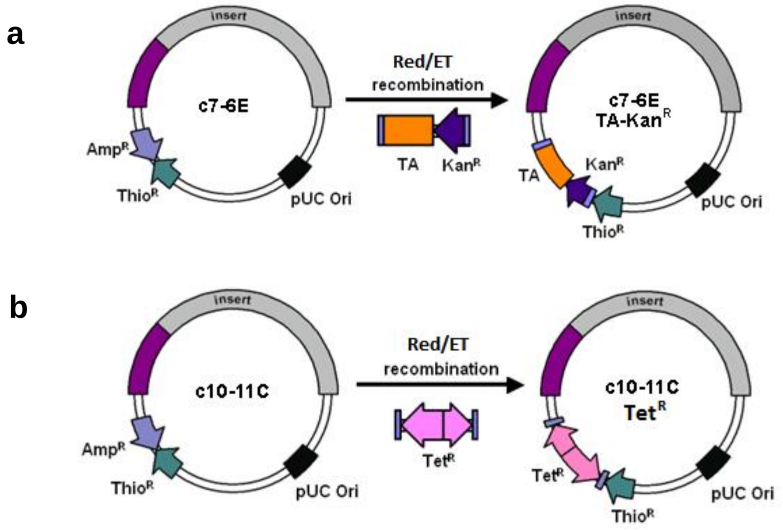

Figure S4. Restriction analysis and PCR examination of two modified cosmids.

a. Restriction analysis of c7-6E TA-Kan^R with *EcoR*I/*Sal*I/*BamH*I. The intended recombination resulted in increased restriction sites of *Sal*I and *BamH*I. The newly generated fragments in *Sal*I and *BamH*I digestion are indicated by the red cycles.

b. PCR confirmation of c7-6E TA-Kan^R using primers TAf/thioF.

c. Restriction analysis of c10-11C Tet^R with *EcoR*I. The intended recombination resulted in one increased restriction site of *EcoR*I. The newly generated fragment is indicated by the red cycle.

d. PCR confirmation of c10-11C Tet^R using primers tetU/thioF.

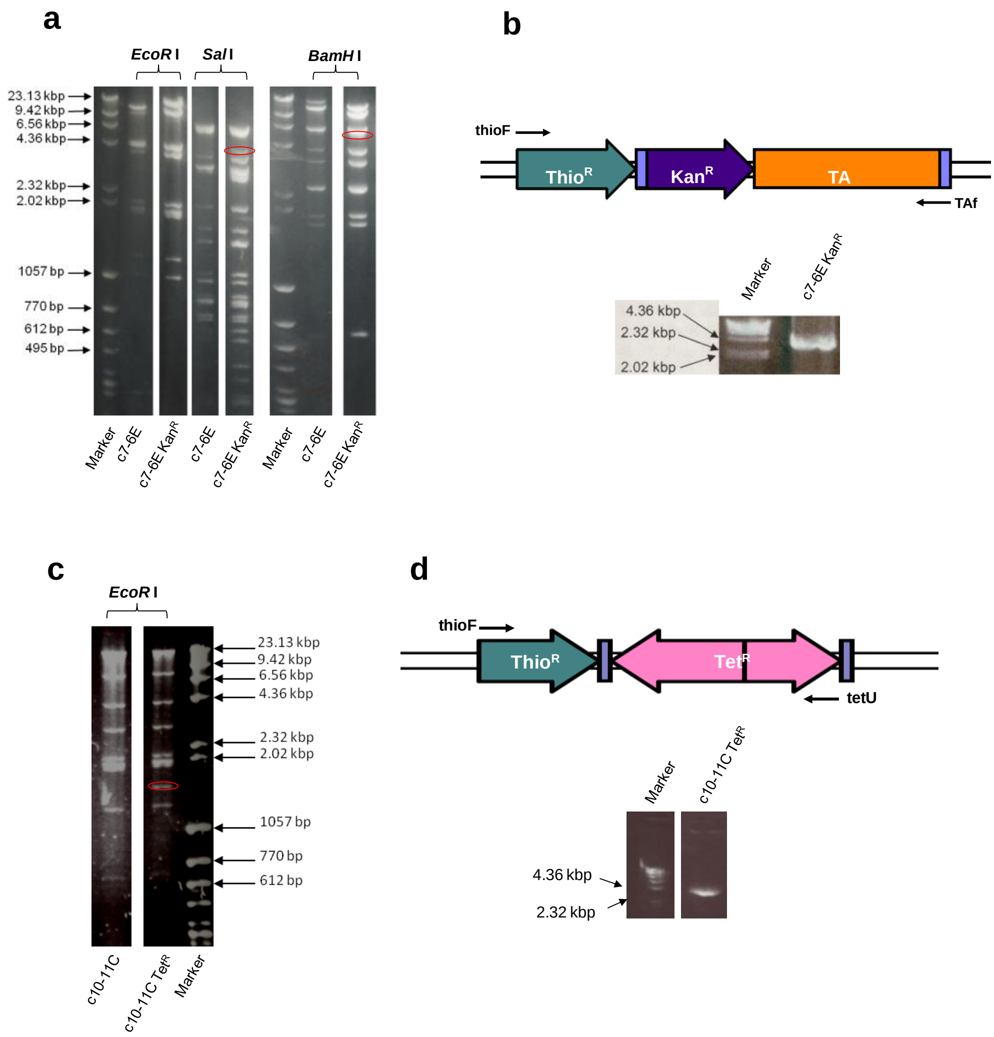

Figure S5. Integration of c6-7E TA-Kan^R and c10-11C Tet^R into *M. xanthus*.

Two modified cosmids that cover the haliangicin biosynthetic gene clusters (*hli*) were integrated into the chromosome of *M. xanthus* to reconstitute *hli* in *M. xanthus*.

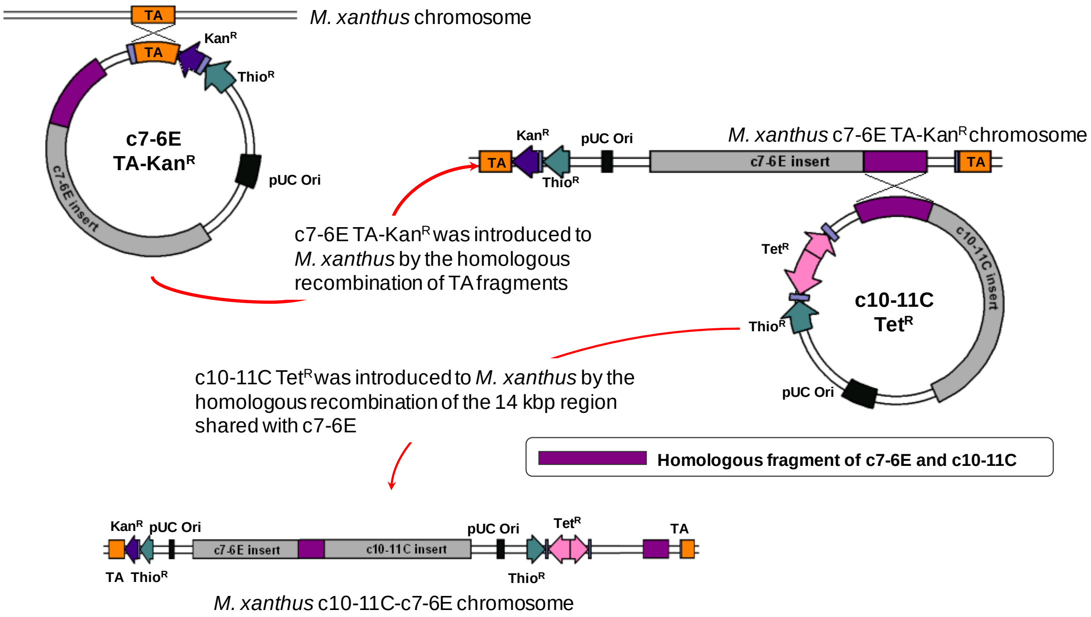

Figure S6. PCR confirmation of the integration of c7-6E TA-Kan^R into the chromosome of *M. xanthus*.

a. PCR confirmation of *M. xanthus* c7-6E TA-Kan^R by primers pair A (HMG-Syn Up/D) and pair B (9-4F-SorD F/R).

b. PCR examination of the homologous recombination of TA fragments by primer pair C (blaF/TAr-R) and pair D (TAf-F/HpaI-kan1).

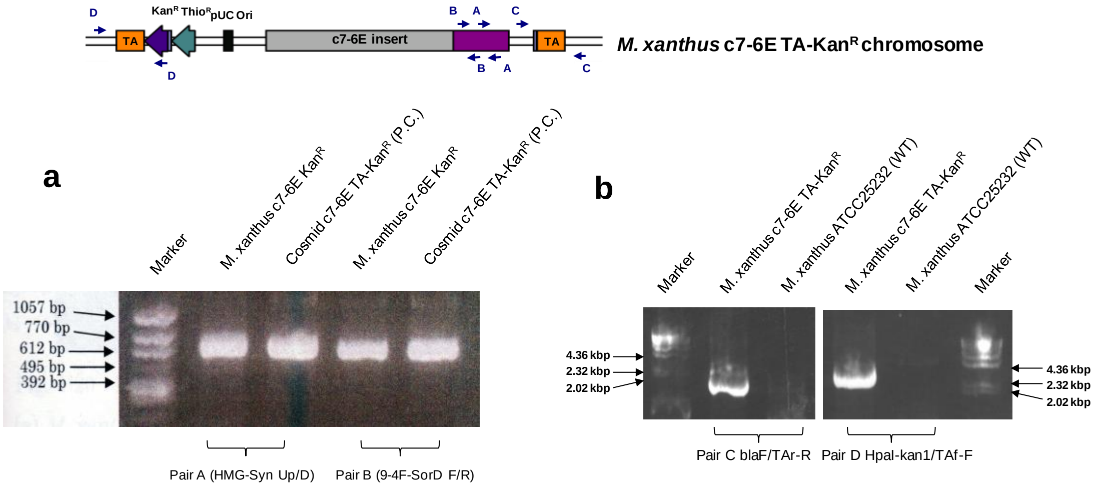

Figure S7. PCR confirmation of the integration of c10-11C Tet^R into the chromosome of *M. xanthus*.

The second integration was confirmed by PCR amplification by primer pair A (HKS4NU/D), pair B (1011R-U/D) and pair C (7-6ENF-F/R)

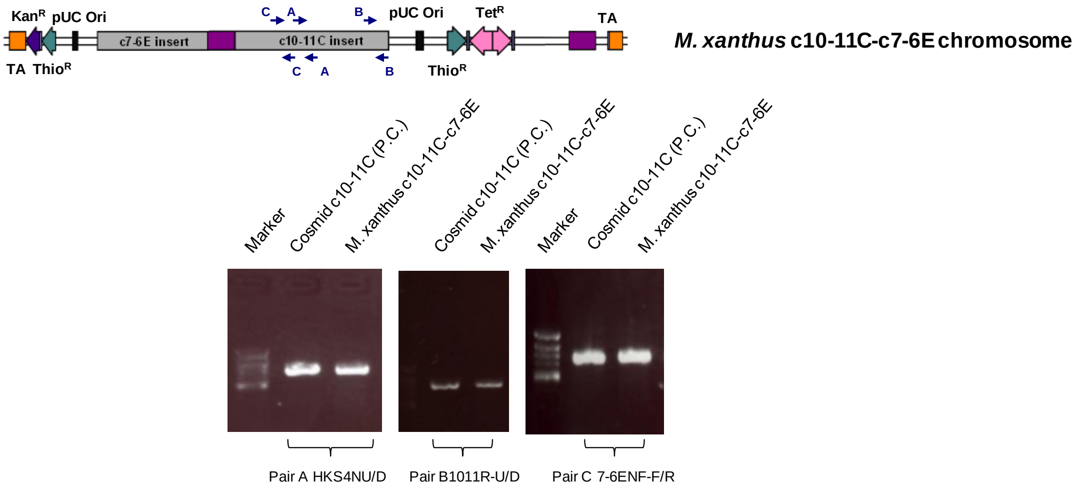

Figure S8. Heterologous haliangicin production profile in *M. xanthus*.

Comparison of haliangicin/*cis*-haliangicin production profile in *M. xanthus* and *H. ochraceum*. The separation was achieved by an isocratic elution [MeOH–H_2O (75:25, v/v), flow rate: 1 mL/min] on a Develosil ODS-UG-5 (ϕ 4.6 × 250 mm) column. UV detection was at 290s nm. Haliangicin (**1**) was accompanied by *cis*-haliangicin (12,13-*cis*-epoxy isomer) in a ratio of 3:2 in the native producer *H. ochraceum*,[1] whereas the heterologous host *M. xanthus* c10-11C-c7-6E strain almost solely produced haliangicin (**1**).

a. Extracts from *H. ochraceum* (haliangicin and cishaliangicin, R_t =25 ~26 min);

b. Extracts from *M. xanthus* c10-11C-c7-6E (haliangicin, R_t =26 min).

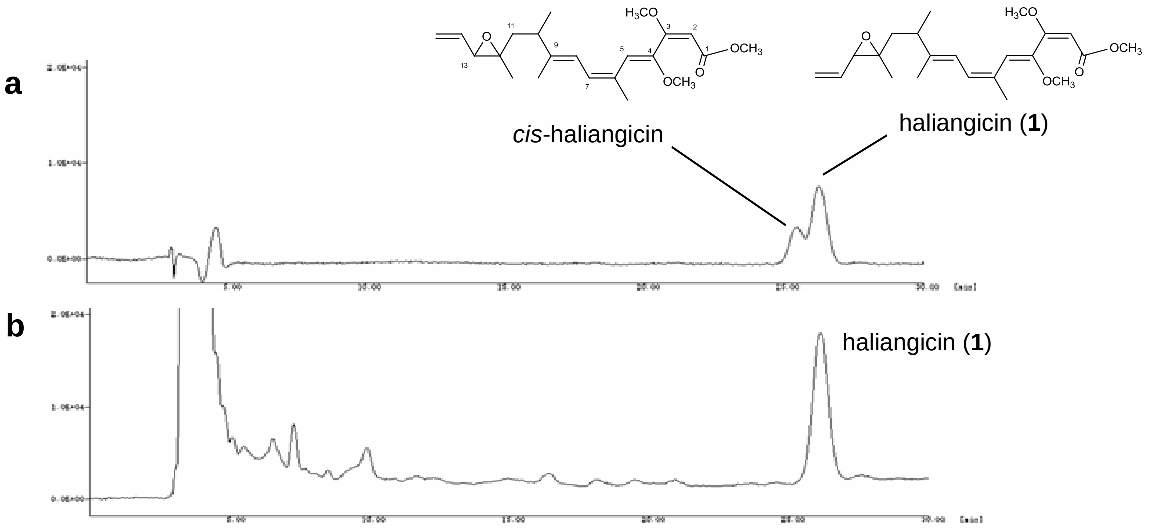

Figure S9. Feeding experiments with labeled precursors.

a. ^{13}C NMR spectrum (150 MHz) of [2-^{13}C]acetate-labeled haliangicin in $CDCl_3$.
b. ^{13}C NMR spectrum (150 MHz) of [1,2-$^{13}C_2$]acetate-labeled haliangicin in $CDCl_3$.
c. ^{13}C NMR spectrum (150 MHz) of [1-^{13}C]propionate-labeled haliangicin in $CDCl_3$.
d. ^{13}C NMR spectrum (150 MHz) of L-[*methyl*-^{13}C]methionine-labeled haliangicin in $CDCl_3$.
e. ^{13}C NMR spectrum (100 MHz) of [U-$^{13}C_3$]glycerol-labeled haliangicin in $CDCl_3$. The two-carbon unit of C-3 and C-4 with vicinal oxygen atoms was derived neither from acetate nor propionate, but from glycerol.[3-7] [U-$^{13}C_3$]glycerol labeled C-3 and C-4 as doublets due to direct ^{13}C-^{13}C coupling. Additional enhancements of acetate-derived carbon signals of C-1~2, C-7~8 and 9-Me might be explained by the metabolism of glycerate via glycolysis that finally reach acetyl-CoA as described in other “glycolate” unit-containing compounds.[4,5]

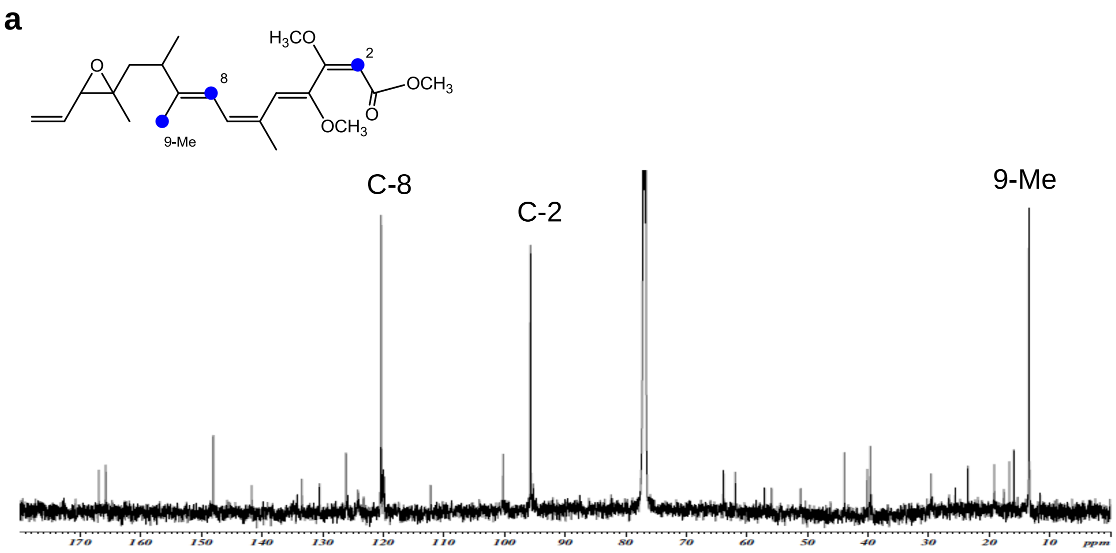

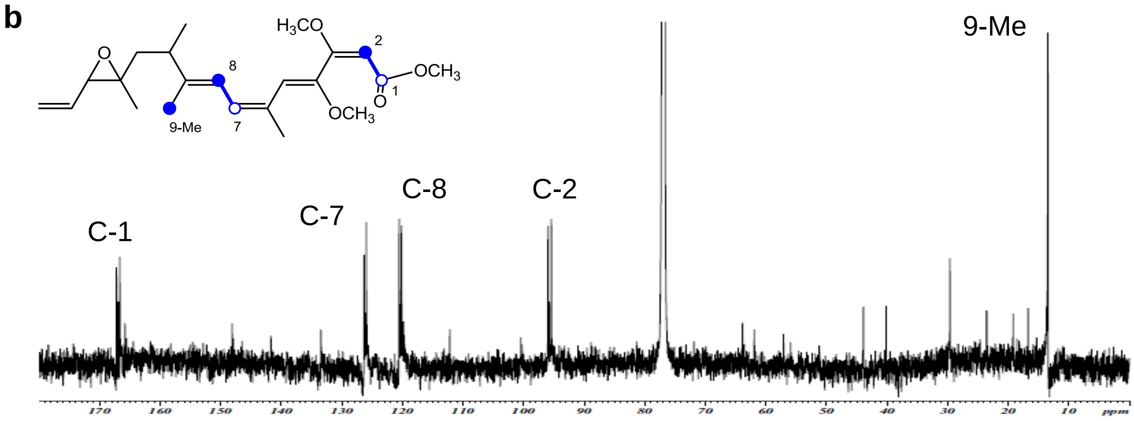

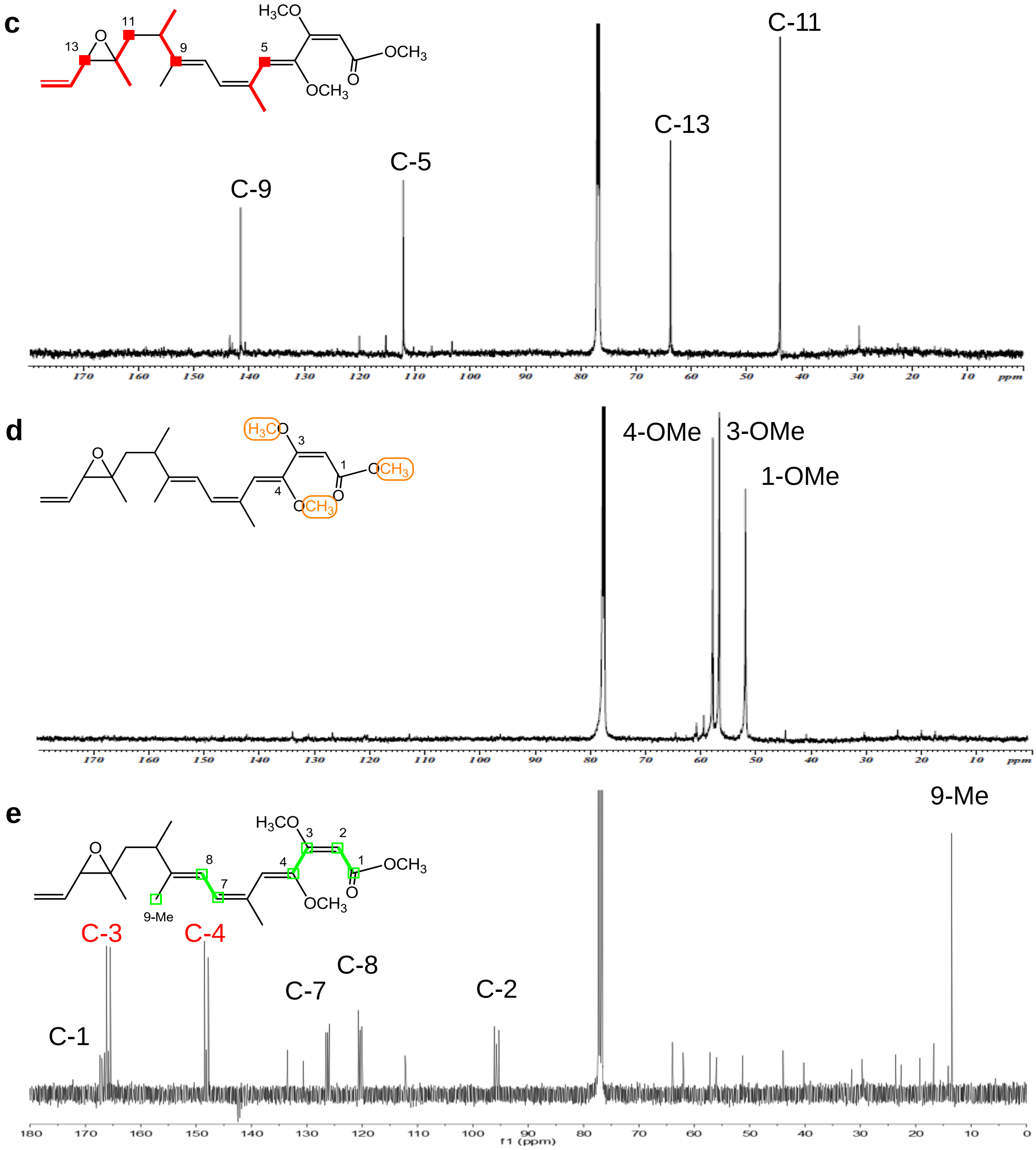
c
11
13
9
5
H_3CO
OCH_3
O
OCH_3
C-9
C-5
C-13
C-11
ppm
d
H_3CO
3
1
OCH_3
O
4
OCH_3
4-OMe
3-OMe
1-OMe
ppm
e
H_3CO
3
2
8
4
1
OCH_3
7
O
OCH_3
9-Me
C-3
C-4
C-7
C-8
C-2
C-1
9-Me
f1 (ppm)

Figure S10. Phylogenetic analysis of KS domains in *hli* genes

Phylogenetic tree generated by NaPDoS web tool. All the KS domains in *hli* are classified into the modular type.

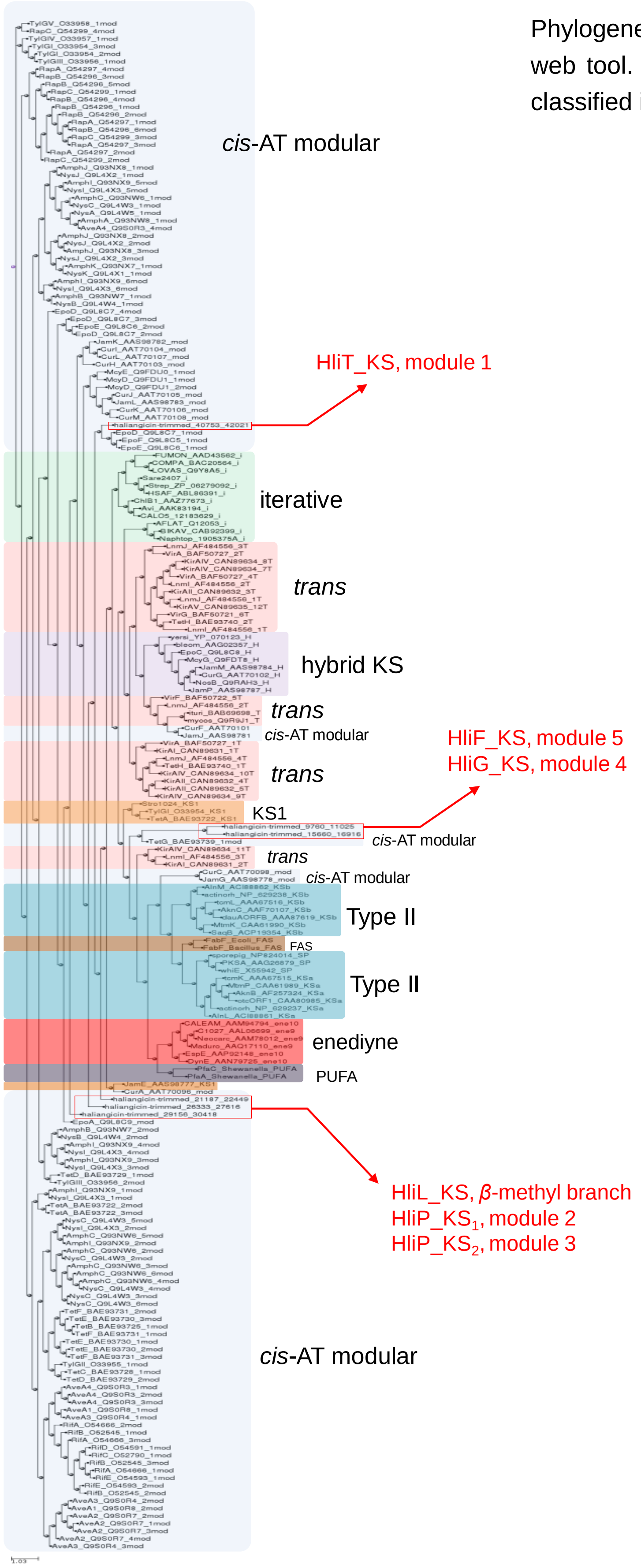

Figure S11. Proposed starter biosynthesis of haliangicin

a. Utilization of propionyl-CoA as starter (hypothesis 1). After starter loading, the haliangicin biosynthesis needs 6 elongation steps by one iterative KS and four typical KSs to generate the heptaketide backbone. However, sequence analysis of KSs indicated no iterative KS in the *hli* gene cluster (Supplementary Fig. S10). Functional characterization of HliR also does not support hypothesis 1 because propionyl-SNAC is not accepted by HliR (Supplementary Fig. S20b).

b. Utilization of dipropionyl-CoA as starter (hypothesis 2). After starter loading, the haliangicin biosynthesis needs 5 elongation steps by five typical KSs to generate the heptaketide backbone. Functional characterization of HliR supports hypothesis 2 because HliR specifically catalyzes the dehydrogenation of the dipropionyl-SNAC (2-methylpent-2-enoyl SNAC) in *in vitro* enzymatic reaction (Fig. 7b).

a hypothesis 1 (monopropionate as starter)

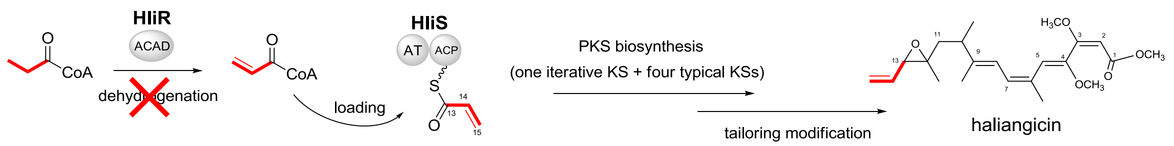

b hypothesis 2 (dipropionate as starter)

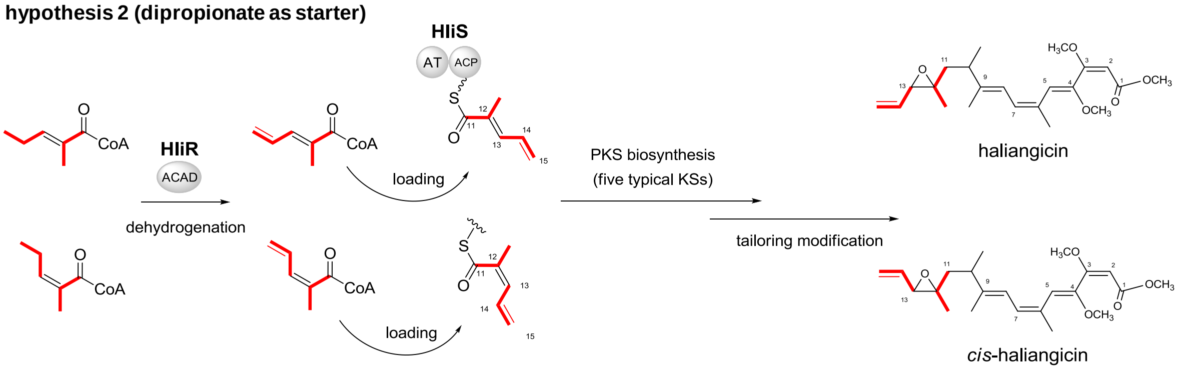

Figure S12. The putative diketide synthases for the biosynthetic formation of the starters.

a. The putative diketide synthase harboring in the genome of *H.ochraceum*. Hoch_0693 (1984 amino acids) encodes four domains: KS(11-466), AT (628-863), KR (1235-1413) and DH (1489-1635), which are just fit for one round of elongation reaction to generate an α,β-unsaturated diketide. The successive Hoch_0692 encodes an acyl-CoA thioesterase which might be involved in the formation of CoA ester of the diketide.

b. The putative diketide synthase harboring in the genome of *M.xanthus*. MXAN_3462 (2216 amino acids) highly resembles Hoch_0693 and consists of five domains: acyl-CoA thioesterase (4-116), AT (213-509), KS (729-985), KR (1640-1868) and DH (1945-2070). The roles of its accompanying enzymes, including MXAN_3456 (radical SAM domain-containing protein), MXAN_3460 (oxidoreductase), MXAN_3461 (short chain dehydrogenase/reductase oxidoreductase), MXAN_3461 (fatty acid desaturase), MXAN_3465 (histidine ammonia-lyase) and MXAN_3466 (aspartate kinase) remain unclear.

a

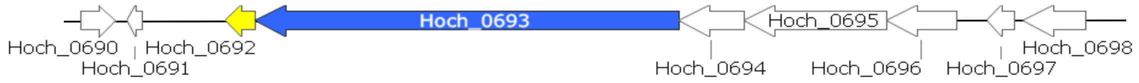

b

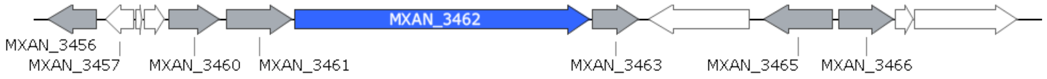

Figure S13. Proposed *β*-methyl branching biochemistry in haliangicin biosynthesis.

Module 2 generates a β-ketoacyl intermediate just fit for one round of *β*-methyl installation biochemistry to install 9-Me at C9. The core biosynthetic machinery for *β*-methyl branching requires at least one HMG-CoA synthase (HCS), one *β*-keto synthase with a Cys-to-Ser mutation (KS^S), one free-standing ACP and two enoyl-CoA hydratases (ECH).[8] *Hli* lacks a *β*-keto synthase (KS^S), which is critical for decarboxylation, leading to a hypothesis that the KS domain in HliL complements this KS^S, although it appears to be a typical KS that retains the conserved triad active residues (CHH). Mueller, *et al.* recently reported a similar bifunctional protein CroD in *Chondromyces crocatus*, in which the KS domain also does not feature the Cys-to-Ser mutation.[9]

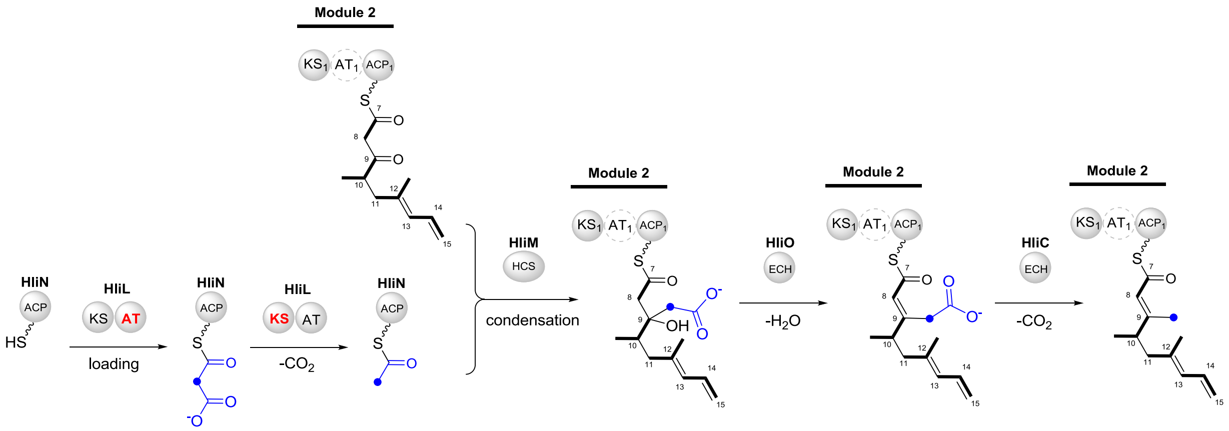

Figure S14. Proposed biosynthetic pathway of methoxymalony-ACP in haliangicin biosynthesis.

The biosynthetic genes responsible for the methoxymalonyl-ACP formation in haliangicin biosynthesis consist of *hli Q*, *H ~ K*, which showed a high similarity to *pelG*, *H ~ K* in the pellasoren biosynthetic gene cluster.[10] Similar to pellasoren, the presence of a multifunctional protein HliQ (AT-ACP-*O*-MT) leads to several functionally redundant proteins. The AT domain of HliQ and HliH are both supposed to recognize 1,3-biphosphoglycerate and load it to an ACP (the ACP of HliQ or the freestanding HliI, which is also presenting in duplicate).

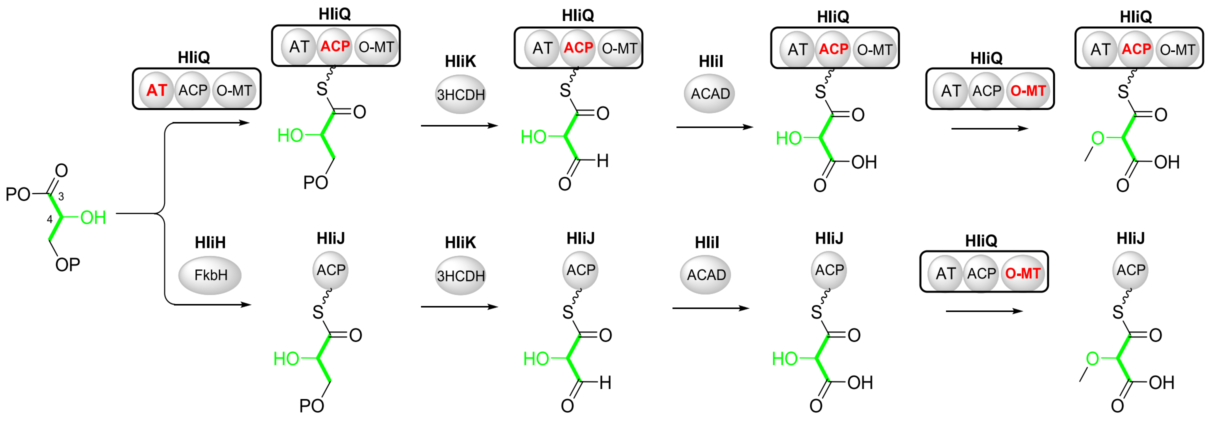

Figure S15. Inactivation of HliR (acyl-CoA dehydrogenase).

a. Disruption of *hliR* via single crossover homologous recombination.

b. Verification of Δ*hliR* mutant by PCR (Pair A: M13f(-47)/ORF18ACAD-Out f; Pair B: M13r(-48)/ ORF18ACAD-Out r; Pair C: ORF18ACAD-Out f/ ORF18ACAD-Out r).

c. Production profile of *M. xanthus* Δ*hliR.* Production of 14,15-dihydrohaliangicin (**4**, t_R = 24.8 min) was observed in HPLC analysis [Develosil ODS-UG-5 (Φ 4.6 × 250 mm), 75% MeOH, 1.0 mL/min, detected at 290 nm].

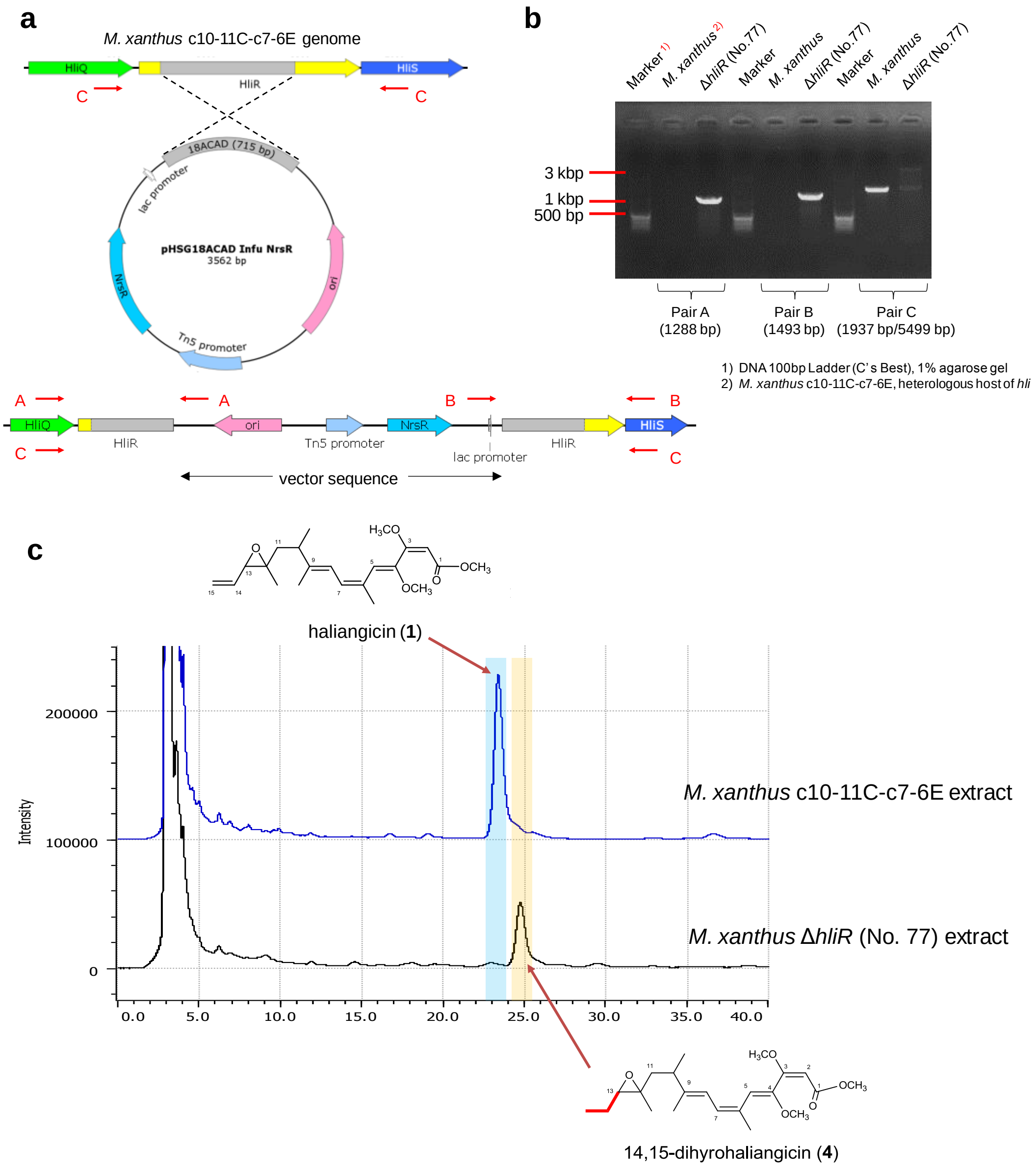

Figure S16. Inactivation of HliD (*O*-methyltransferase).

a. Disruption of *hliD* via single crossover homologous recombination.

b. Verification of Δ*hliD* mutant by PCR. (Pair A: M13r(-48)/ ORF4-MT-Ex-f1; Pair B: M13f(-47)/ ORF4-MT-Ex-r1; Pair C: ORF4-MT-Ex-f1/ ORF4-MT-Ex-r1).

c. Production profile of *M. xanthus* Δ*hliD*. Production of 1-*O*-demethylhaliangicin (**6**, R_t = 36.5 min) and 1-*O*-demethyl-12,13-deoxylhaliangicin (**5**, R_t = 41 min) were observed in HPLC analysis [Develosil ODS-UG-5 (*Φ* 4.6 × 250 mm), linear gradient elution of 20% to 100% (40 min) methanol in H_2O, 1.0 mL/min, detected at 290 nm].

d. LC-MS analysis of *M. xanthus* Δ*hliD* extract. Conditions: Cadenza CD-C18 (2 x 75 mm), 30 to 100% (35 min) MeOH-0.1% HCOOH, 0.1 mL/min (1/20 split ratio), sample 20 uL broth eq.

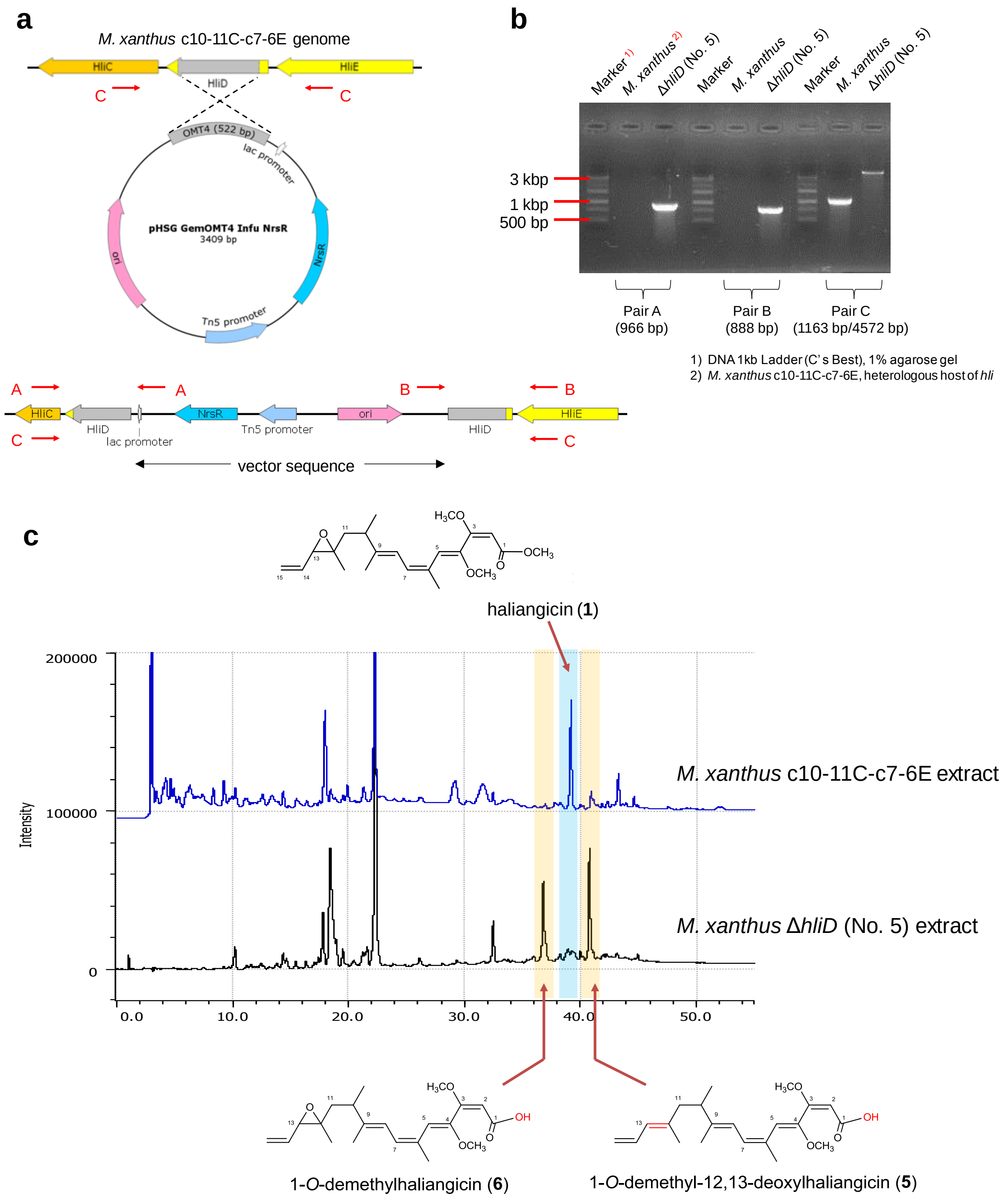

d

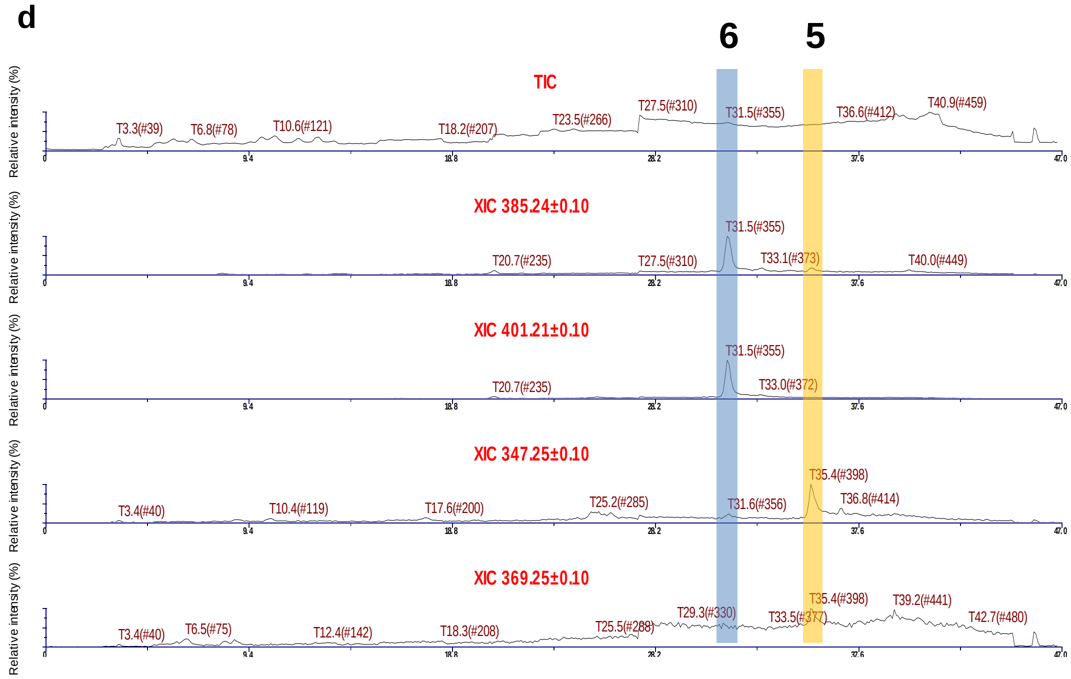
6
5
TIC
XIC 385.24±0.10
XIC 401.21±0.10
XIC 347.25±0.10
XIC 369.25±0.10
Relative intensity (%)

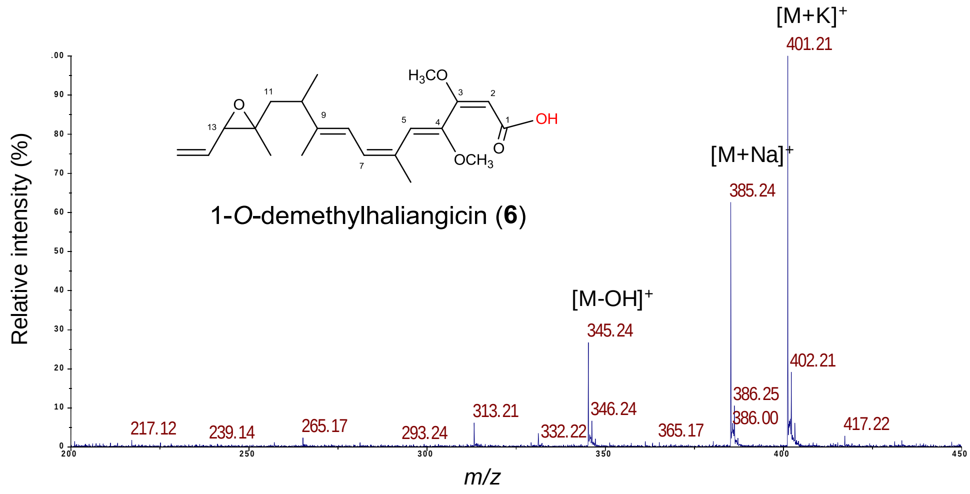
1-O-demethylhaliangicin (6)
[M+K]+
401.21
[M+Na]+
385.24
[M-OH]+
345.24
Relative intensity (%)
m/z

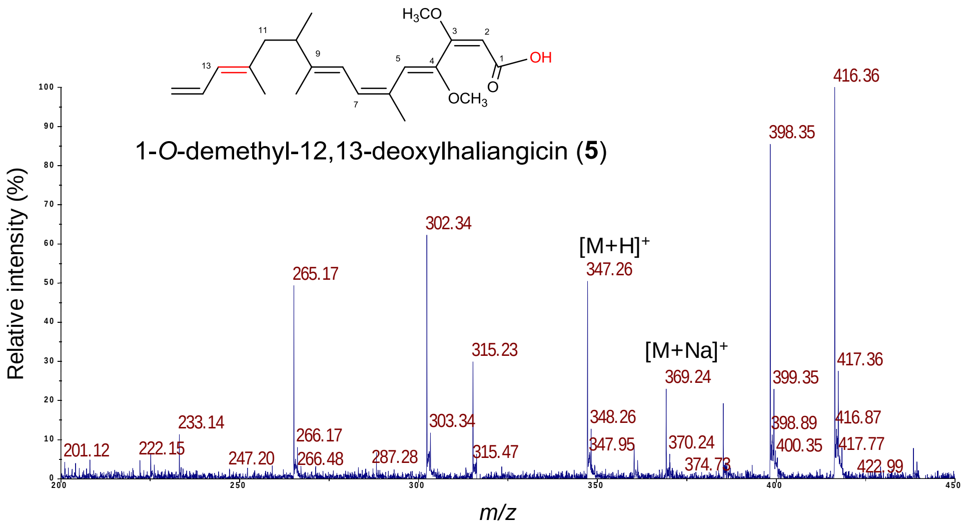
1-O-demethyl-12,13-deoxylhaliangicin (5)
[M+H]+
347.26
[M+Na]+
369.24
416.36
398.35
302.34
265.17
Relative intensity (%)
m/z

Figure S17. Methyl esterification of *M. xanthus* Δ*hliD* extracts using TMS diazomethane.

HPLC analysis of *M. xanthus* Δ*hliD* extracts before (blue) and after (red) methyl esterification by diazomethane, comparing with authentic sample of 12,13-deoxylhaliangicin (**7**, orange) and haliangicin (**1**, green).

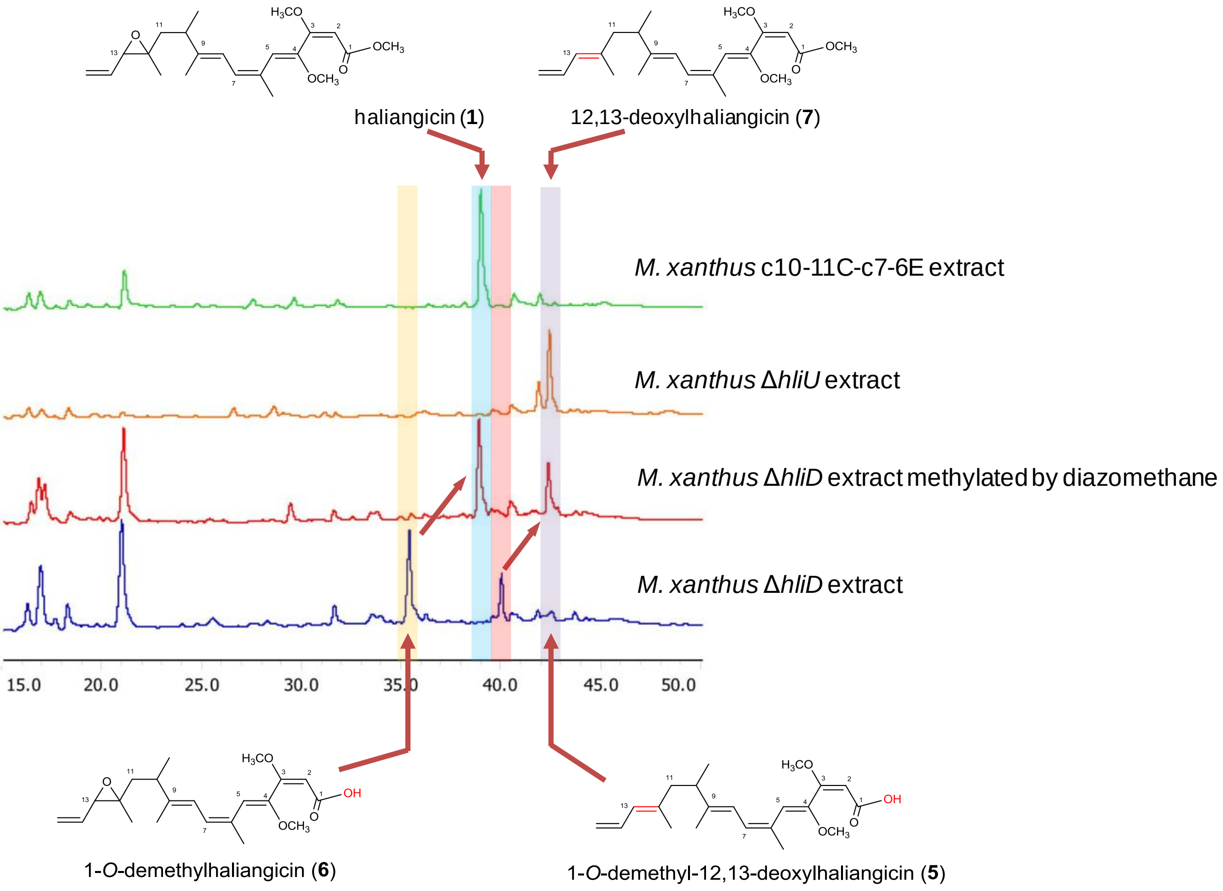

Figure S18. Inactivation of HliU (epoxidase).

a. Disruption of *hliU* via single crossover homologous recombination.

b. Verification of Δ*hliU* mutant by PCR. (Pair A: M13f(-47)/ ORF21Ox-Out F2; Pair B: M13r(-48)/ ORF21Ox-Out R; Pair C: ORF21Ox-Out F2/ ORF21Ox-Out R).

c. Production profile of *M. xanthus* Δ*hliU.* Production of 12,13-deoxylhaliangicin (**7**, t_R = 43 min) was observed in HPLC analysis [Develosil ODS-UG-5 (Φ 4.6 × 250 mm), linear gradient elution of 20% to 100% (40 min) methanol in H_2O, 1.0 mL/min, detected at 290 nm]

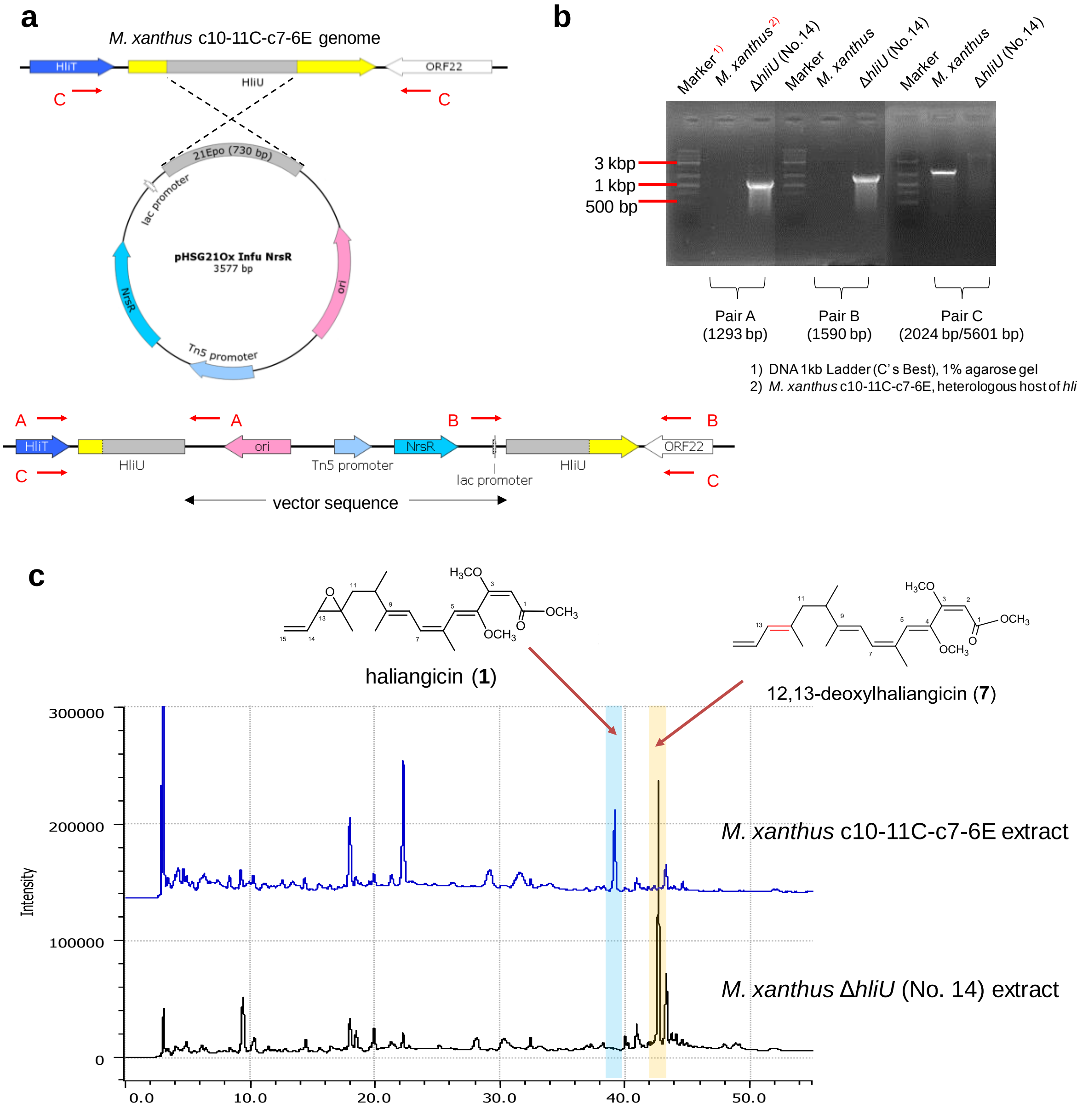

Figure S19. Expression and purification of recombinant HliR.

SDS-PAGE (10% Tris-HCl gel) of recombinant HliR. Marker: Precision Plus Protein™ Dual Color Standards (BioRad)

a. Soluble protein extracted from *E. coli* KRX pET32a HliR with/without induction of 0.1% rhamnose and 0.4 mM IPTG

b. Purified N-His$_6$-HliR

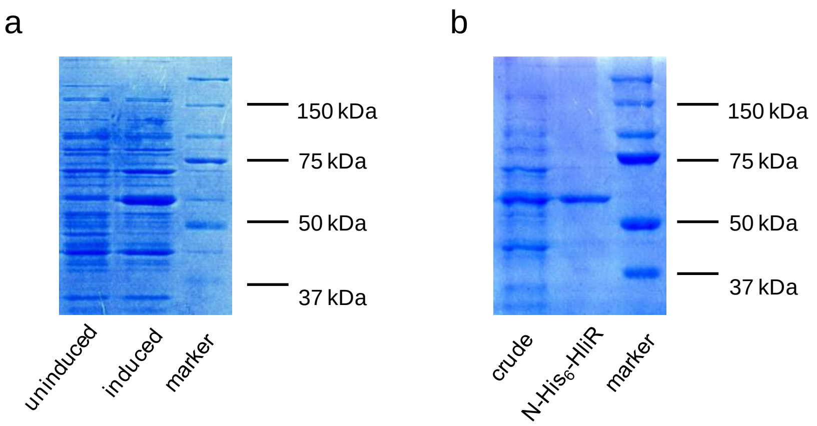

Figure S20. ***In vitro*** **enzymatic reaction of recombinant HliR.**

HPLC analyses of the HliR enzymatic reactions using 14,15-dihydrohaliangicin (**4**) (**a**), propionyl SNAC (**b**), butyryl SNAC (**c**), valeryl SNAC (**d**), hexanoyl SNAC (**e**) heptanoyl SNAC (**f**), 2-methylvaleryl SNAC (**g**), 4-methylvaleryl SNAC (**h**), 4-pentenoyl SNAC (**i**), 2-methyl-4-pentenoyl SNAC (**j**), cyclo-1-pentenoyl SNAC (**k**), cyclo-1-hexenoyl SNAC (**l**)

HPLC condition: Develosil ODS-UG-5 (ϕ 4.6 × 250 mm); 75% methanol, flow rate 1.0 mL/min, and UV detection at 290 nm for **a;** 50% methanol, flow rate 0.6 mL/min, and UV detection at 239 nm for **b**; 60% methanol, flow rate 0.6 mL/min, and UV detection at 254 nm for **c**-**e**, **g**-**l**; 75% methanol, flow rate 0.6 mL/min, and UV detection at 254 nm for **f**;

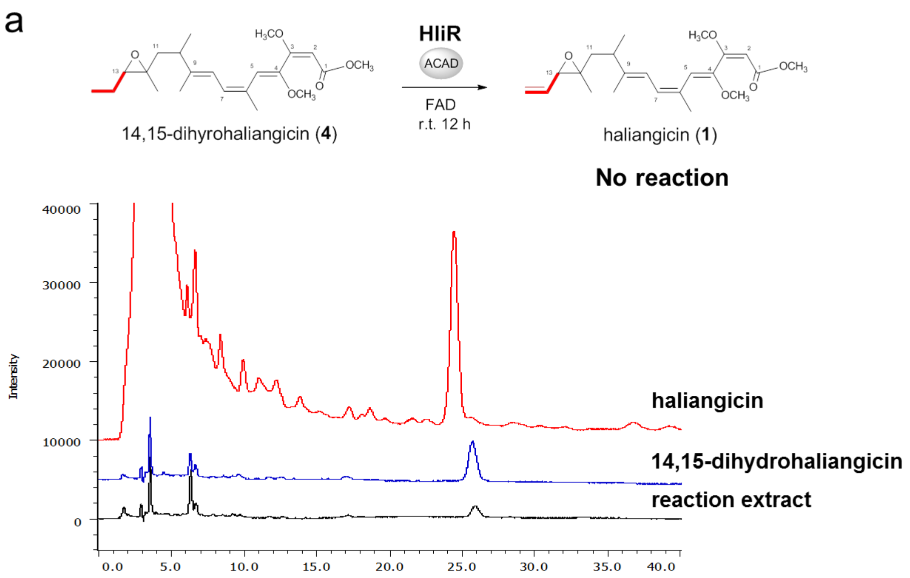

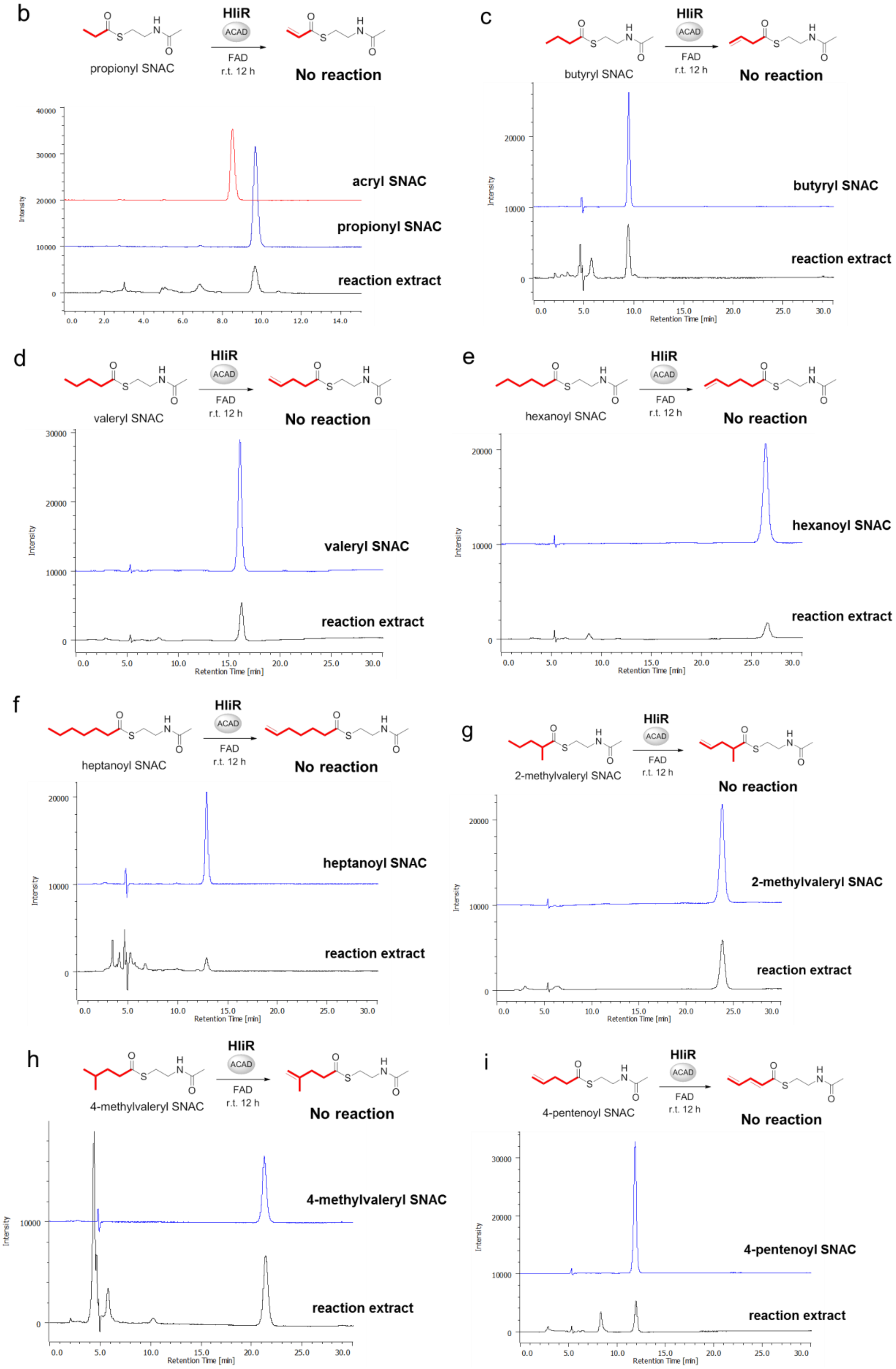
b
HliR
ACAD
FAD
r.t. 12 h
propionyl SNAC
No reaction
acryl SNAC
propionyl SNAC
reaction extract
Intensity
c
HliR
ACAD
FAD
r.t. 12 h
butyryl SNAC
No reaction
butyryl SNAC
reaction extract
Retention Time [min]
d
HliR
ACAD
FAD
r.t. 12 h
valeryl SNAC
No reaction
valeryl SNAC
reaction extract
e
HliR
ACAD
FAD
r.t. 12 h
hexanoyl SNAC
No reaction
hexanoyl SNAC
reaction extract
f
HliR
ACAD
FAD
r.t. 12 h
heptanoyl SNAC
No reaction
heptanoyl SNAC
reaction extract
g
HliR
ACAD
FAD
r.t. 12 h
2-methylvaleryl SNAC
No reaction
2-methylvaleryl SNAC
reaction extract
h
HliR
ACAD
FAD
r.t. 12 h
4-methylvaleryl SNAC
No reaction
4-methylvaleryl SNAC
reaction extract
i
HliR
ACAD
FAD
r.t. 12 h
4-pentenoyl SNAC
No reaction
4-pentenoyl SNAC
reaction extract

j

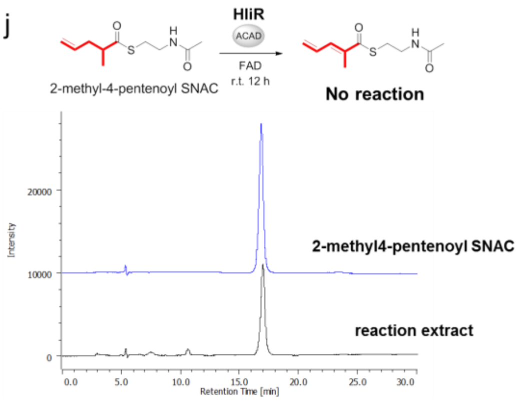
HliR
ACAD
FAD
r.t. 12 h
2-methyl-4-pentenoyl SNAC
No reaction
2-methyl4-pentenoyl SNAC
reaction extract
Intensity
Retention Time [min]

k

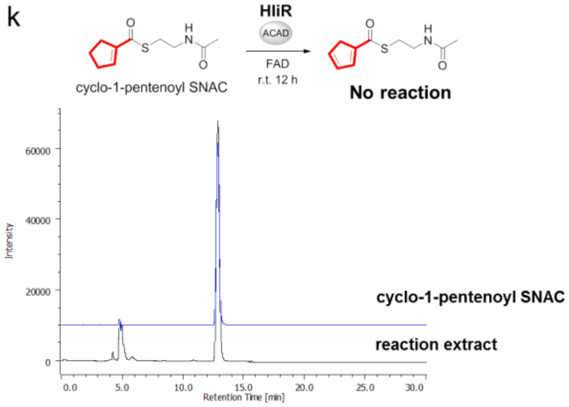
HliR
ACAD
FAD
r.t. 12 h
cyclo-1-pentenoyl SNAC
No reaction
cyclo-1-pentenoyl SNAC
reaction extract
Intensity
Retention Time [min]

l

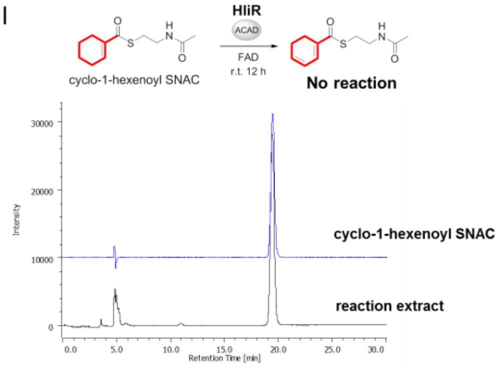
HliR
ACAD
FAD
r.t. 12 h
cyclo-1-hexenoyl SNAC
No reaction
cyclo-1-hexenoyl SNAC
reaction extract
Intensity
Retention Time [min]

Figure S21. Spectra of 14,15-dihydrohaliangicin (4).

a. ^{1}H NMR (400 MHz, $CDCl_3$); **b.** ^{13}C NMR (100 MHz, $CDCl_3$); **c.** HMQC; **d.** NOESY; **e.** UV; **f.** IR; **g.** ESI-MS

a

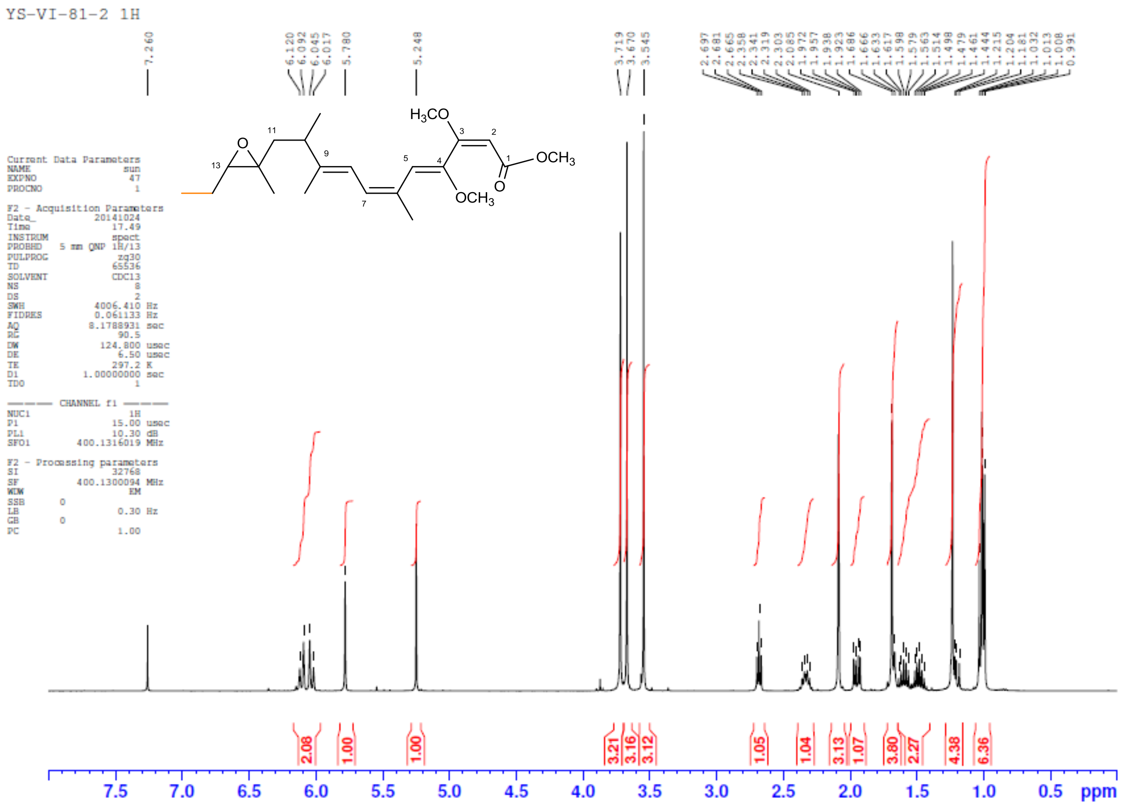

b

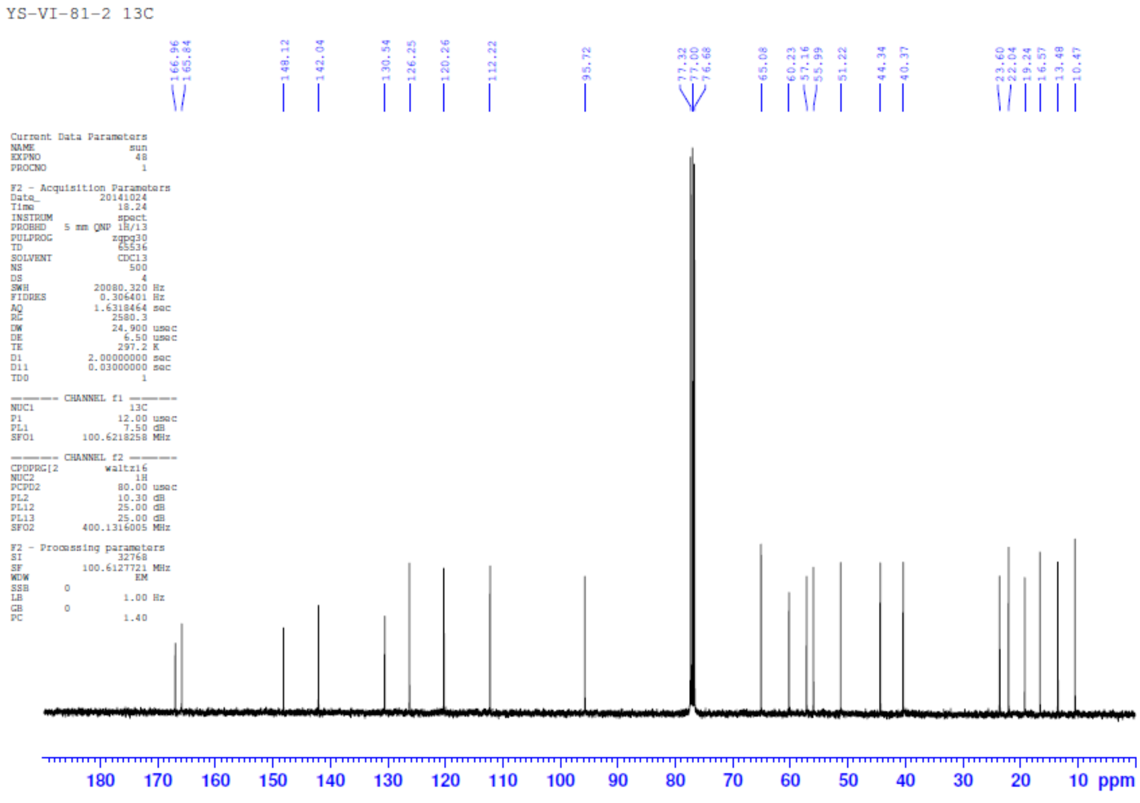

c

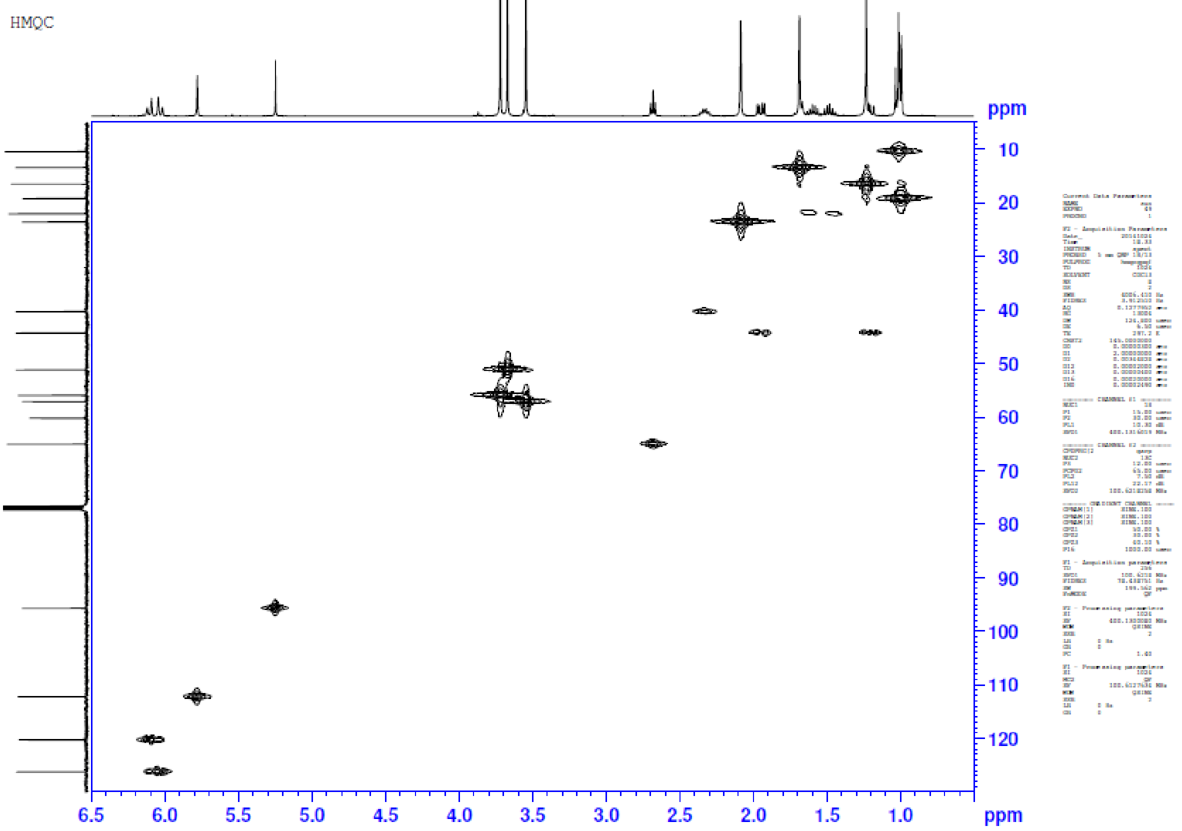

d

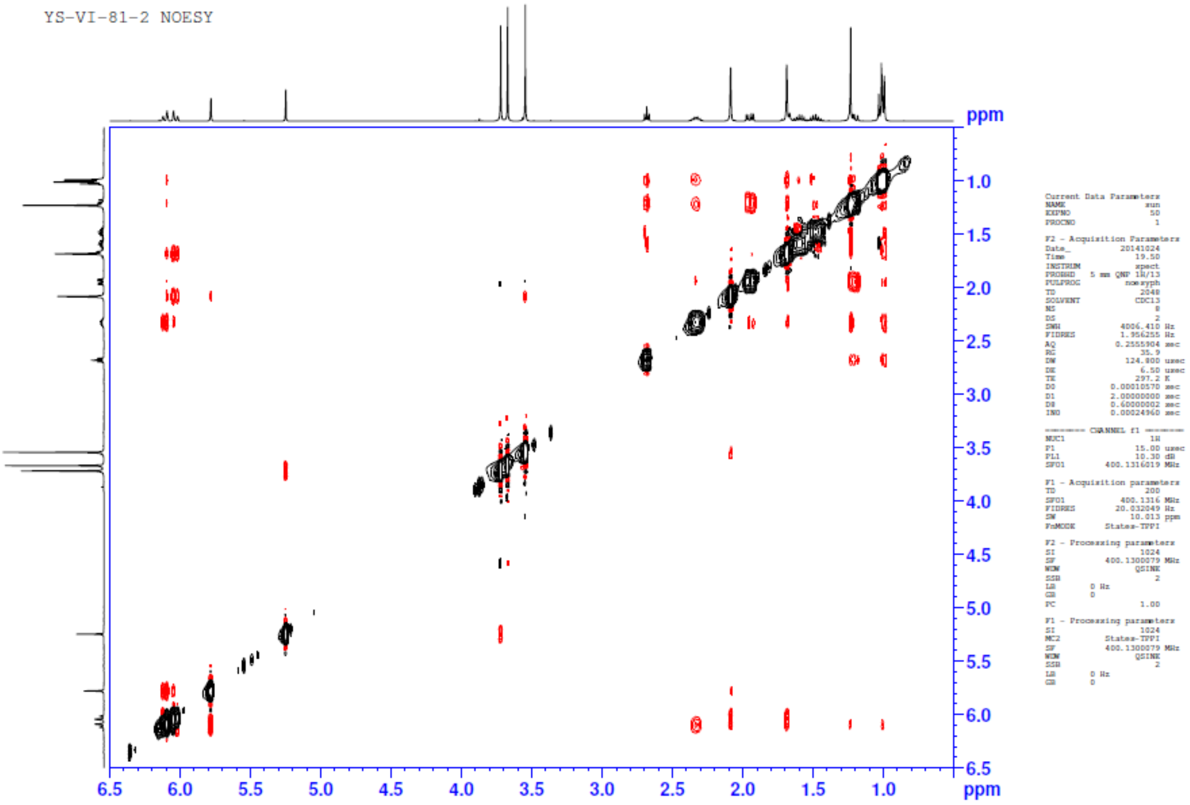

e

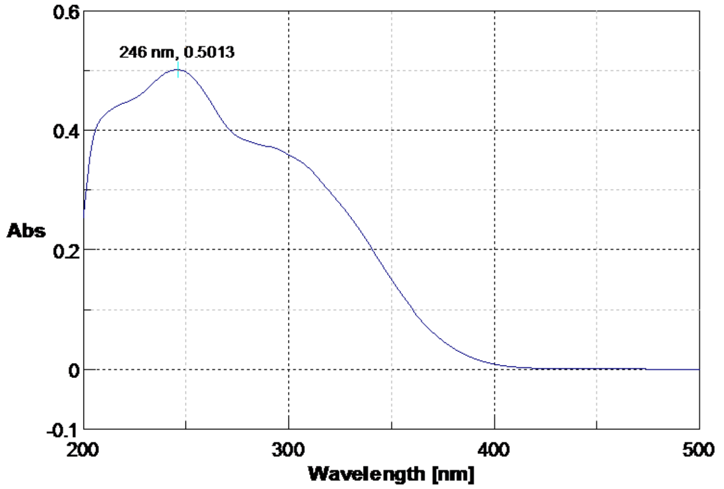

f

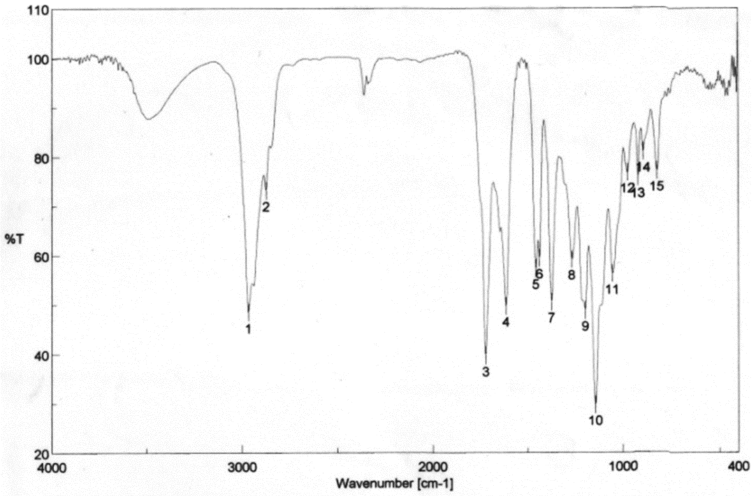

Results of Peak Find

No.	Position	Intensity	No.	Position	Intensity	No.	Position	Intensity	No.	Position	Intensity
1	2967.91	48.4289	2	2876.31	73.1488	3	1721.16	39.6228	4	1614.13	49.7053
5	1456.96	57.284	6	1437.67	59.4077	7	1374.03	50.5085	8	1266.04	59.1661
9	1195.65	48.8217	10	1142.62	29.7048	11	1054.87	56.2141	12	976.769	76.7474
13	923.736	75.6548	14	896.737	80.8557	15	824.42	77.0239			

g

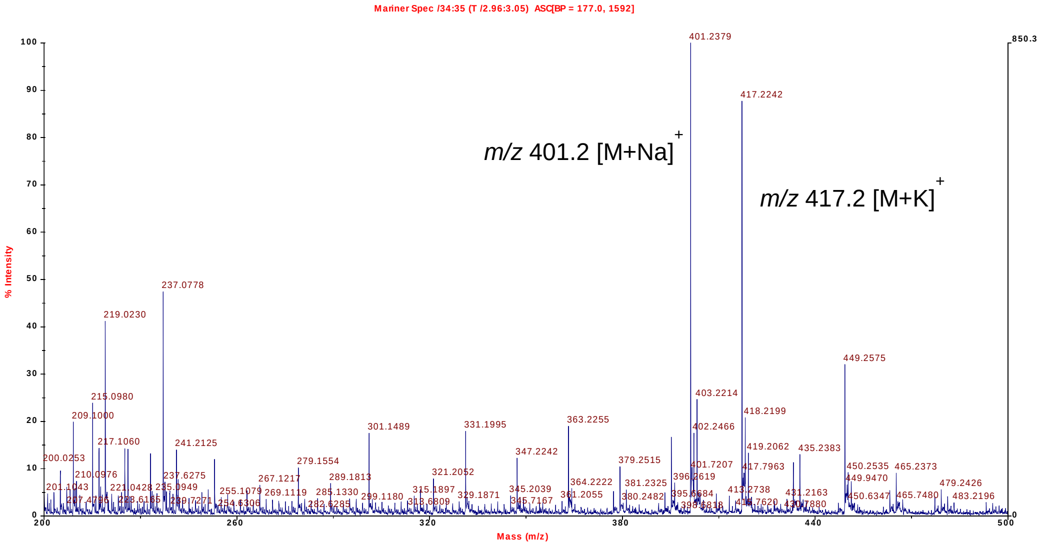

Figure S22. Spectra of 12,13-deoxylhaliangicin (7).

a. ^{1}H NMR (400 MHz, $CDCl_3$); **b.** ^{13}C NMR (100 MHz, $CDCl_3$); **c.** HMQC; **d.** NOESY; **e.** UV; **f.** IR; **g.** ESI-MS.

a

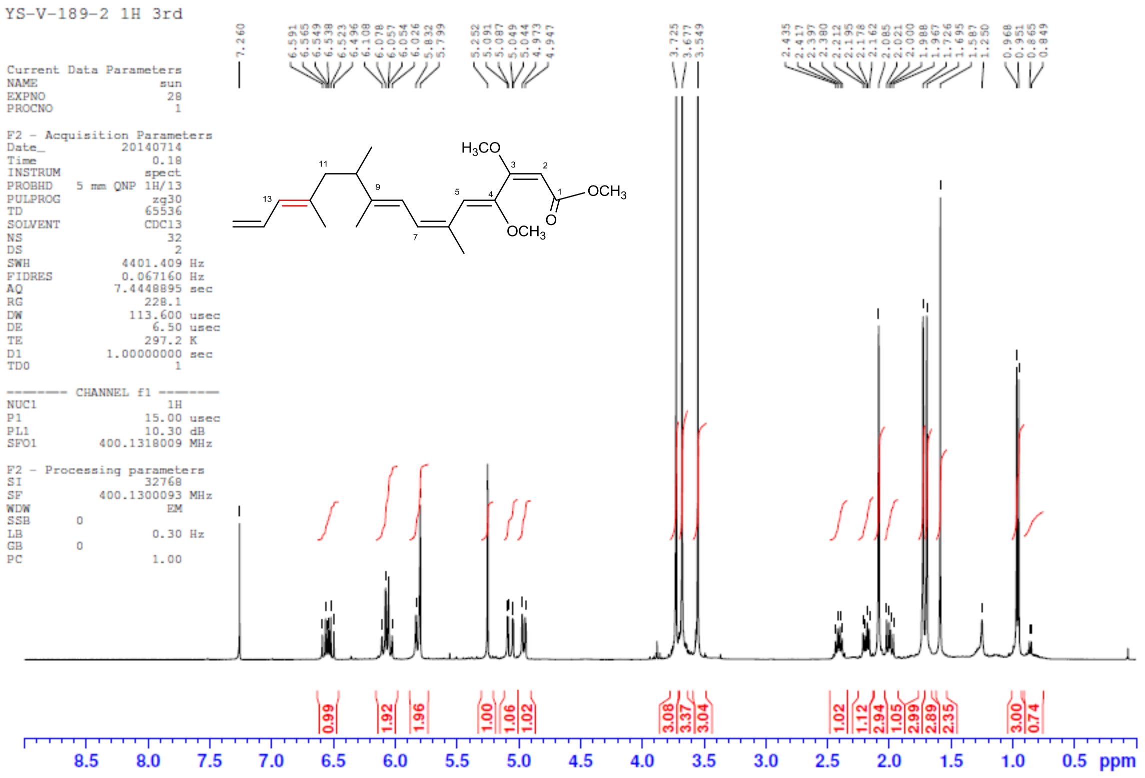

b

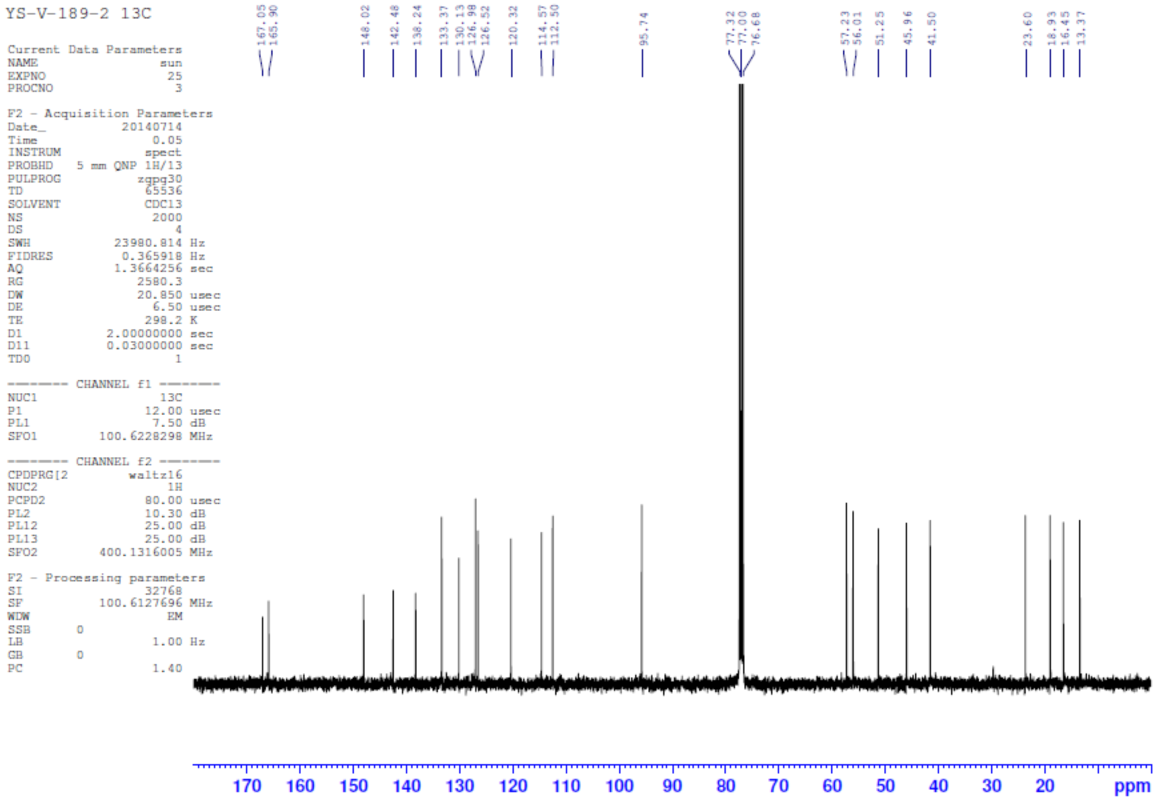

c

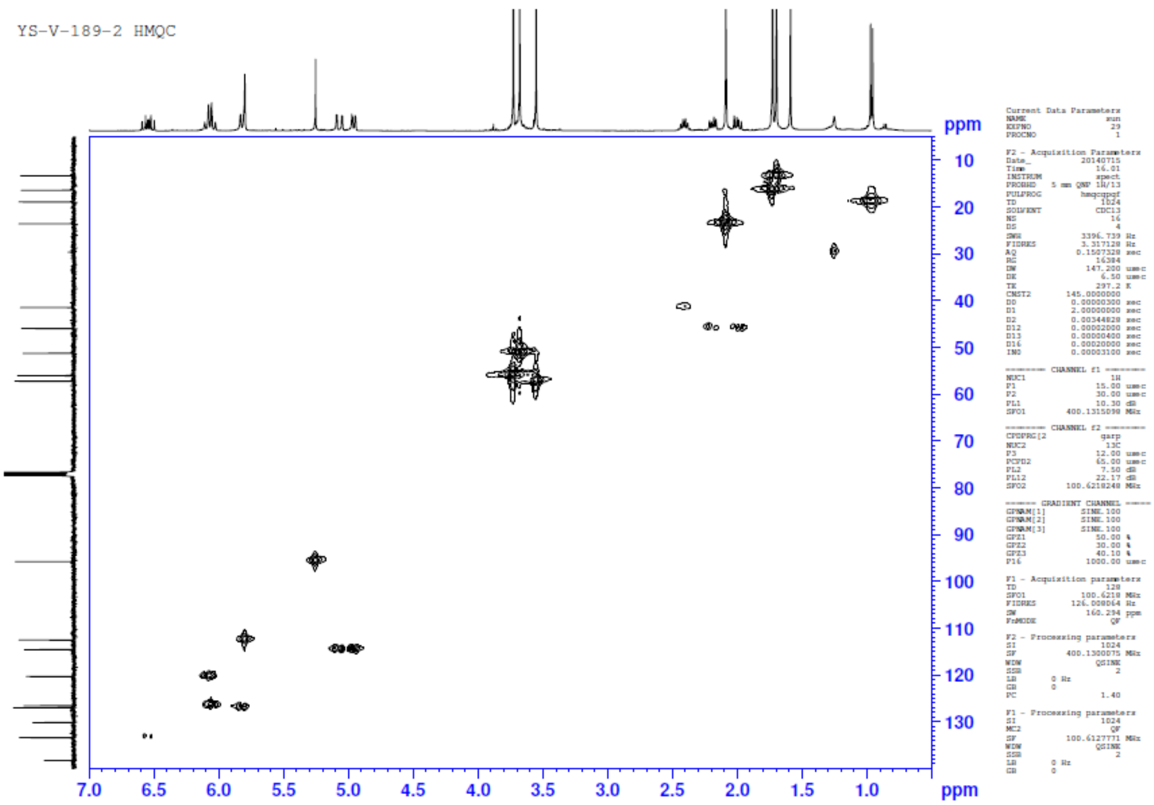
YS-V-189-2 HMQC
ppm
Current Data Parameters

d

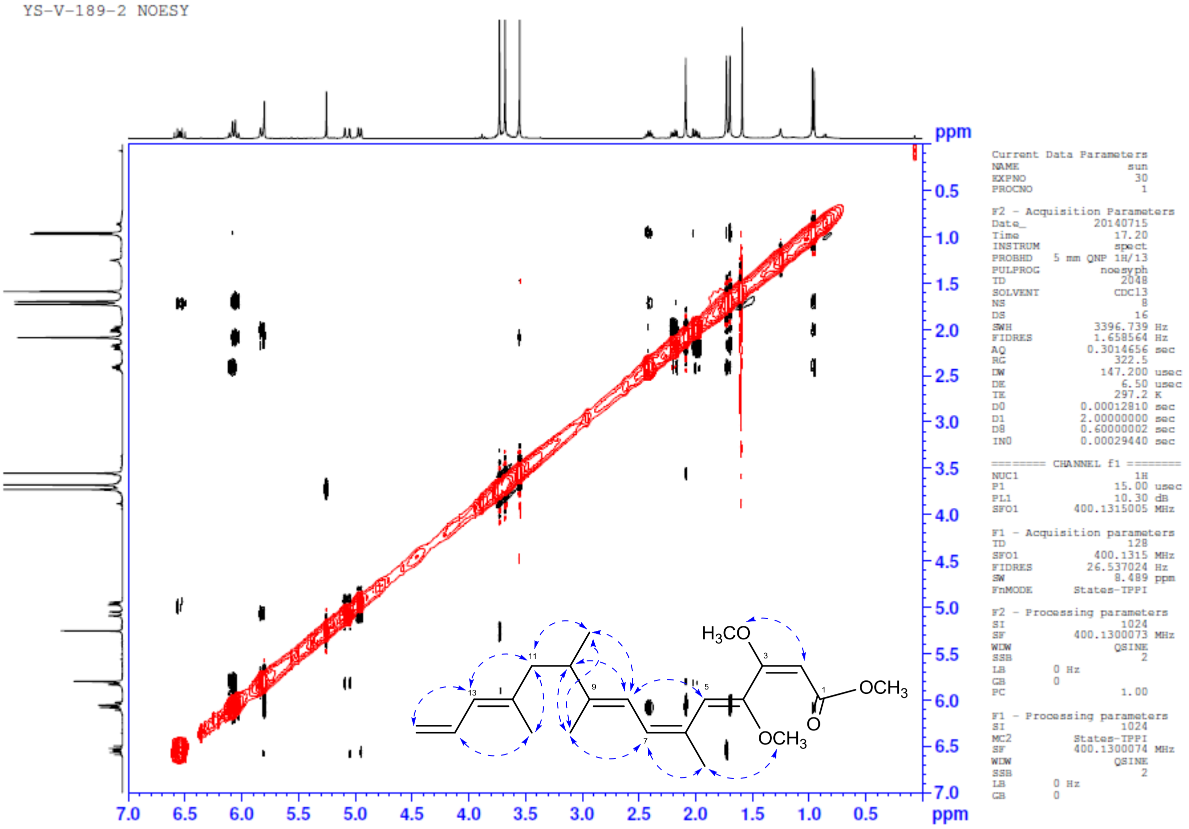
YS-V-189-2 NOESY
ppm
Current Data Parameters
H3CO
OCH3
OCH3
O

e

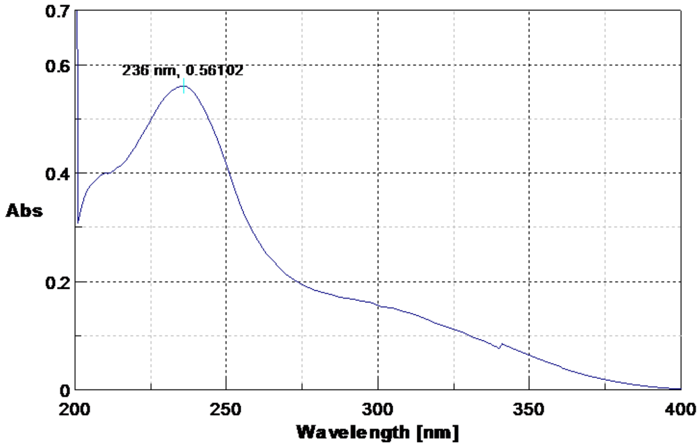

f

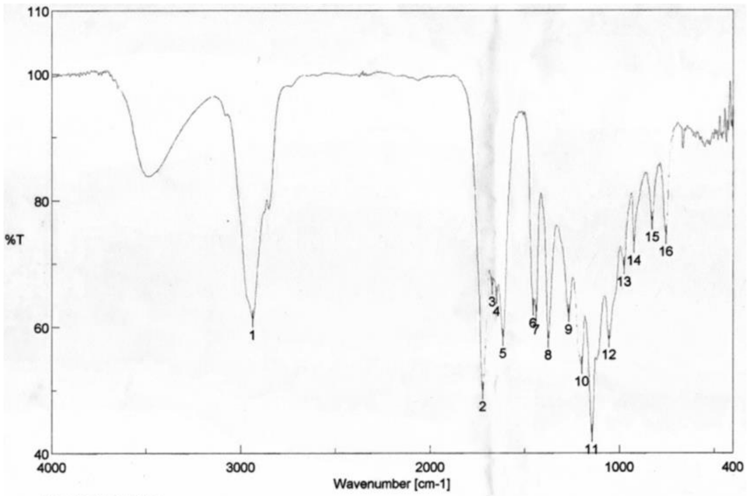

Results of Peak Find

No.	Position	Intensity	No.	Position	Intensity	No.	Position	Intensity	No.	Position	Intensity
1	2937.06	61.2488	2	1721.16	49.9209	3	1671.98	66.4737	4	1648.84	64.9475
5	1614.13	58.2573	6	1454.06	63.1251	7	1437.67	62.0902	8	1373.07	58.0122
9	1265.07	62.0927	10	1196.61	53.818	11	1142.62	43.1098	12	1053.91	58.0824
13	976.769	69.6259	14	923.736	72.9976	15	827.312	76.701	16	754.995	74.3712

g

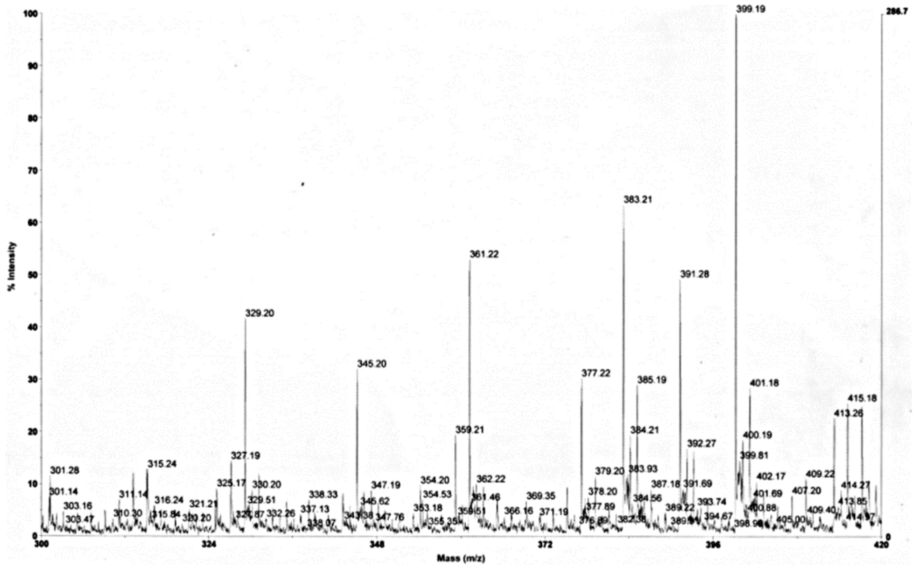

Figure S23. NMR spectra of haliangicin (1).

a. ^{1}H NMR (400 MHz, $CDCl_3$); **b.** ^{13}C NMR (100 MHz, $CDCl_3$).

a

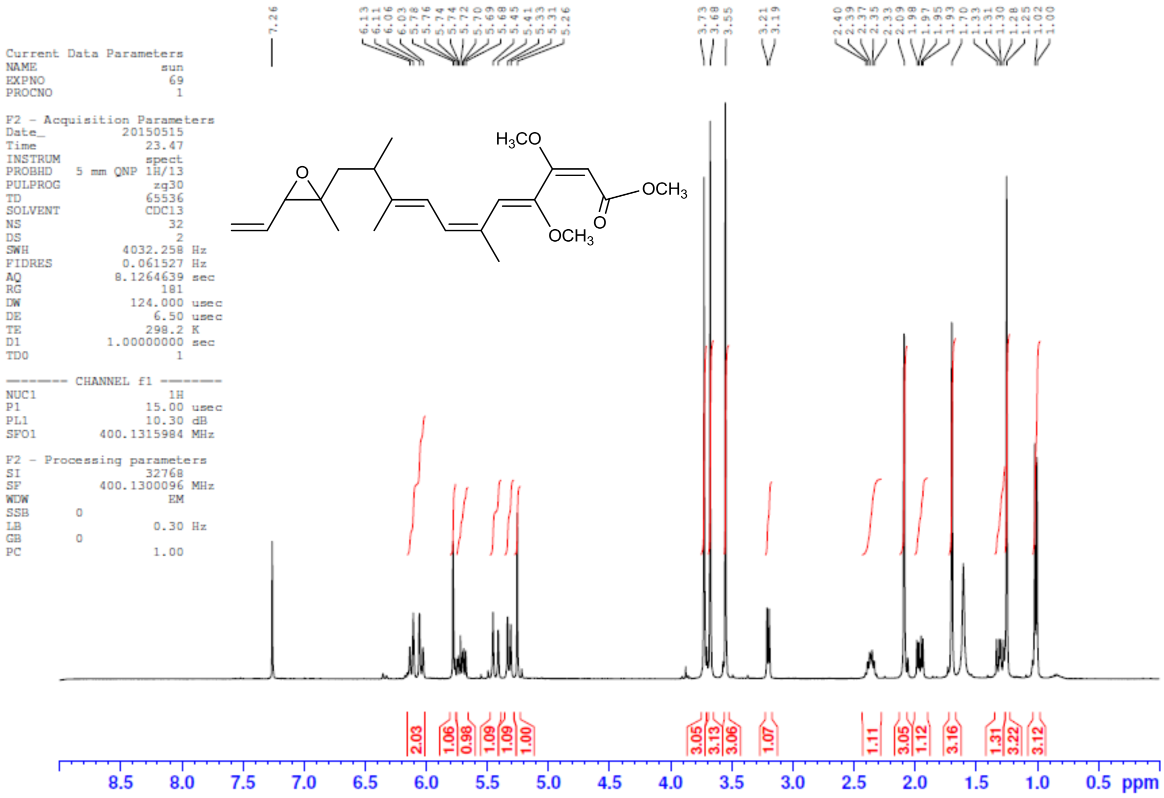

b

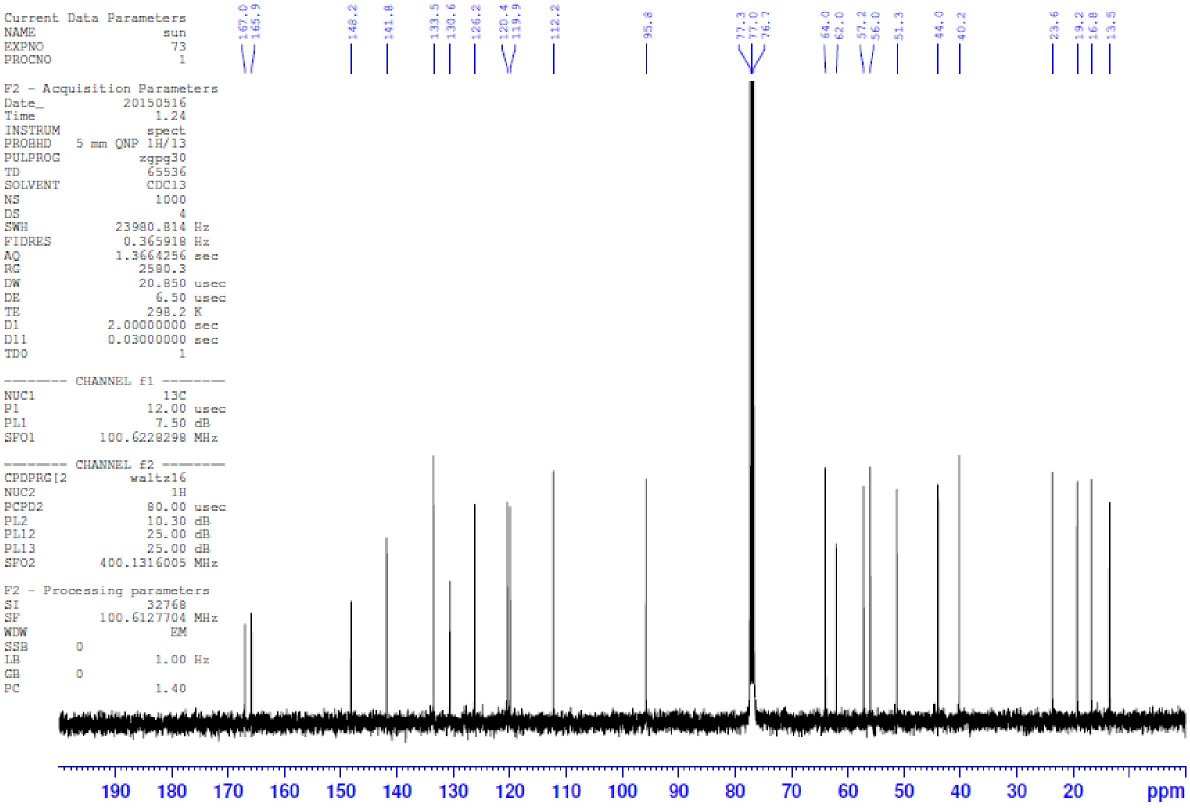

Figure S24. NMR spectra of 2-methylpent-2,4-dienoyl SNAC (3a).

a. ^{1}H NMR (400 MHz, $CDCl_3$); **b.** ^{13}C NMR (100 MHz, $CDCl_3$).

a

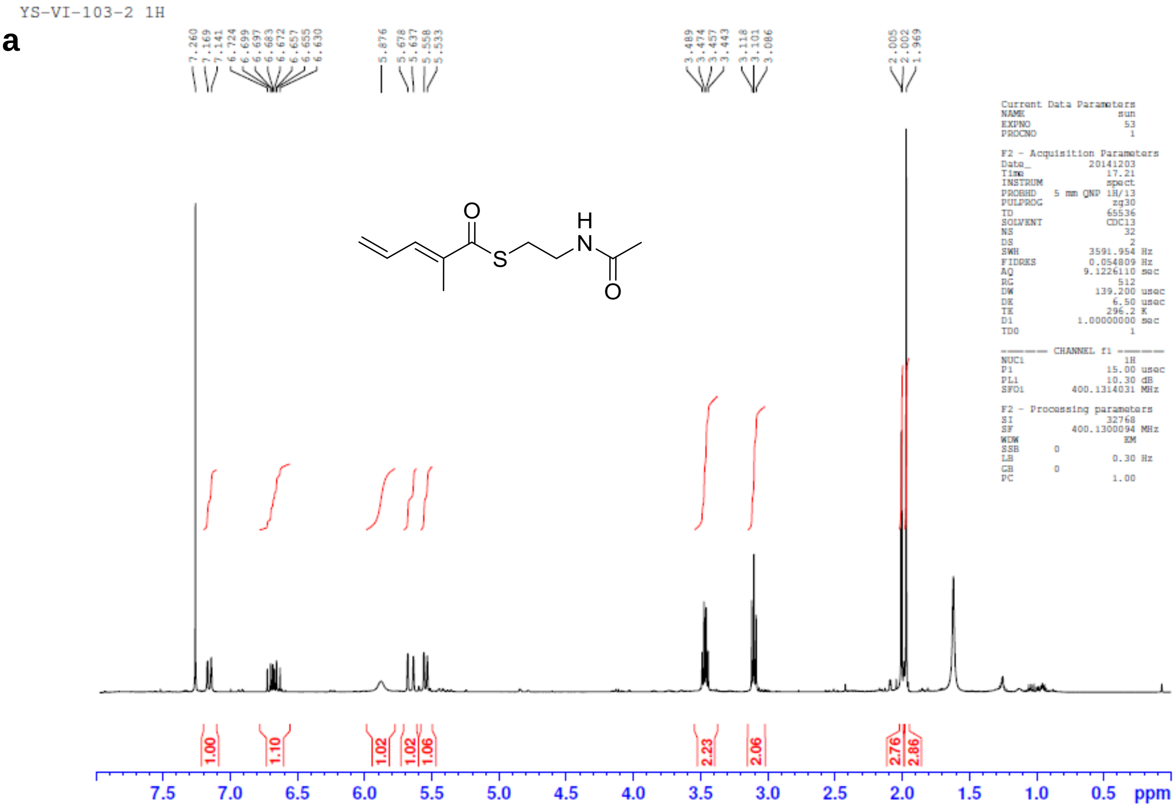

b

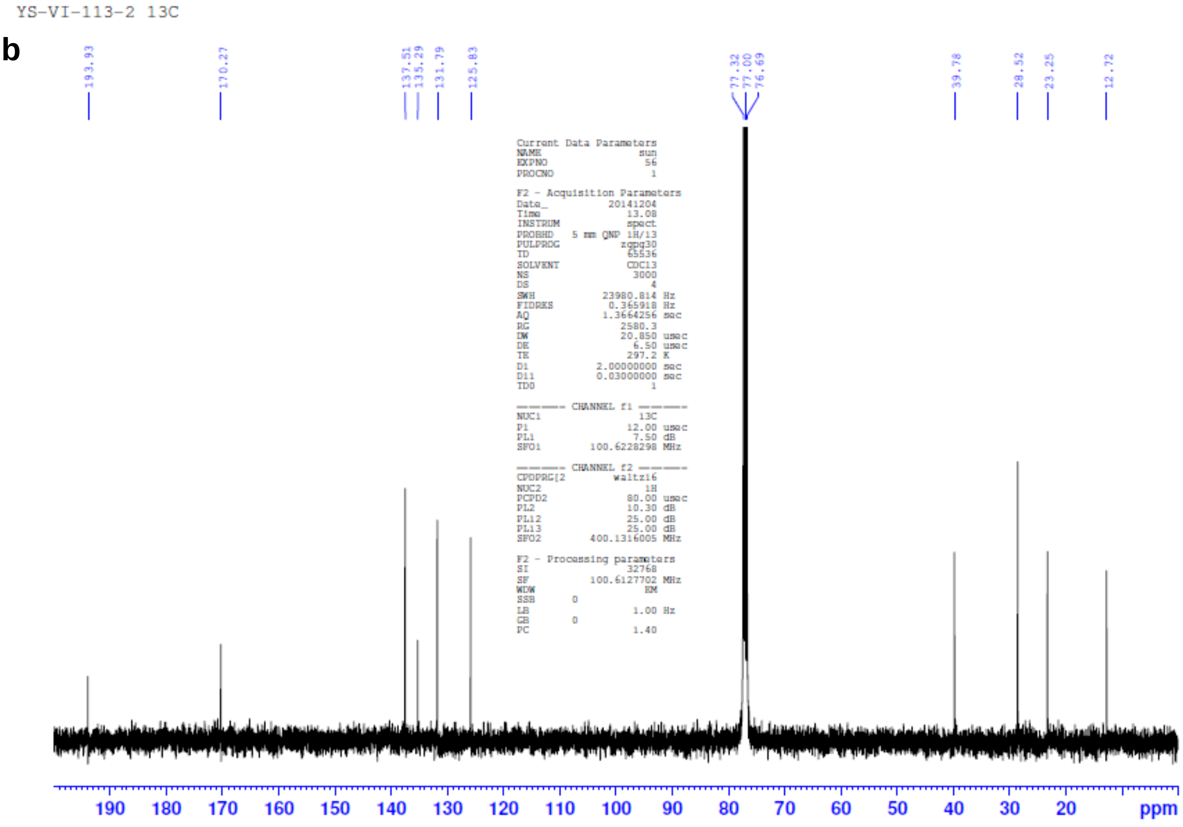

Figure S25. NMR spectra of pent-2,4-dienoyl SNAC (3b).

a. ^{1}H NMR (400 MHz, $CDCl_3$); **b.** ^{13}C NMR (100 MHz, $CDCl_3$).

a

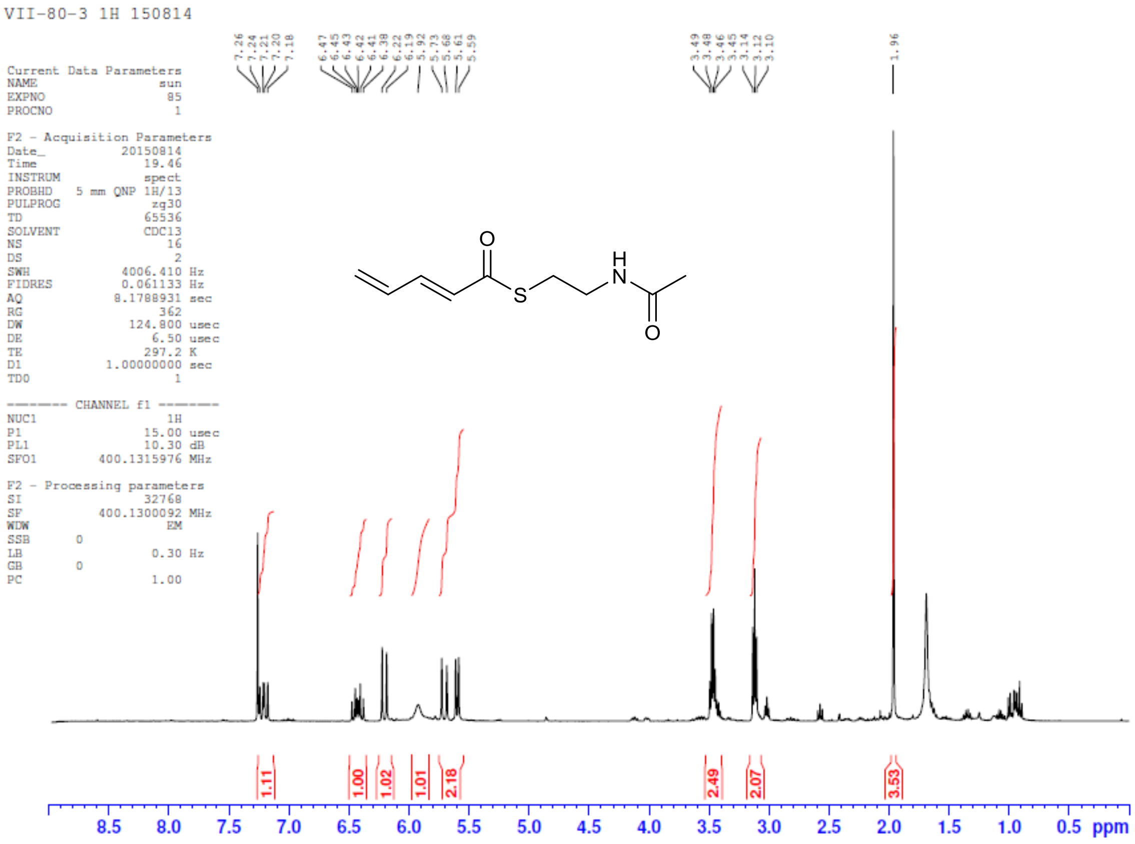

b

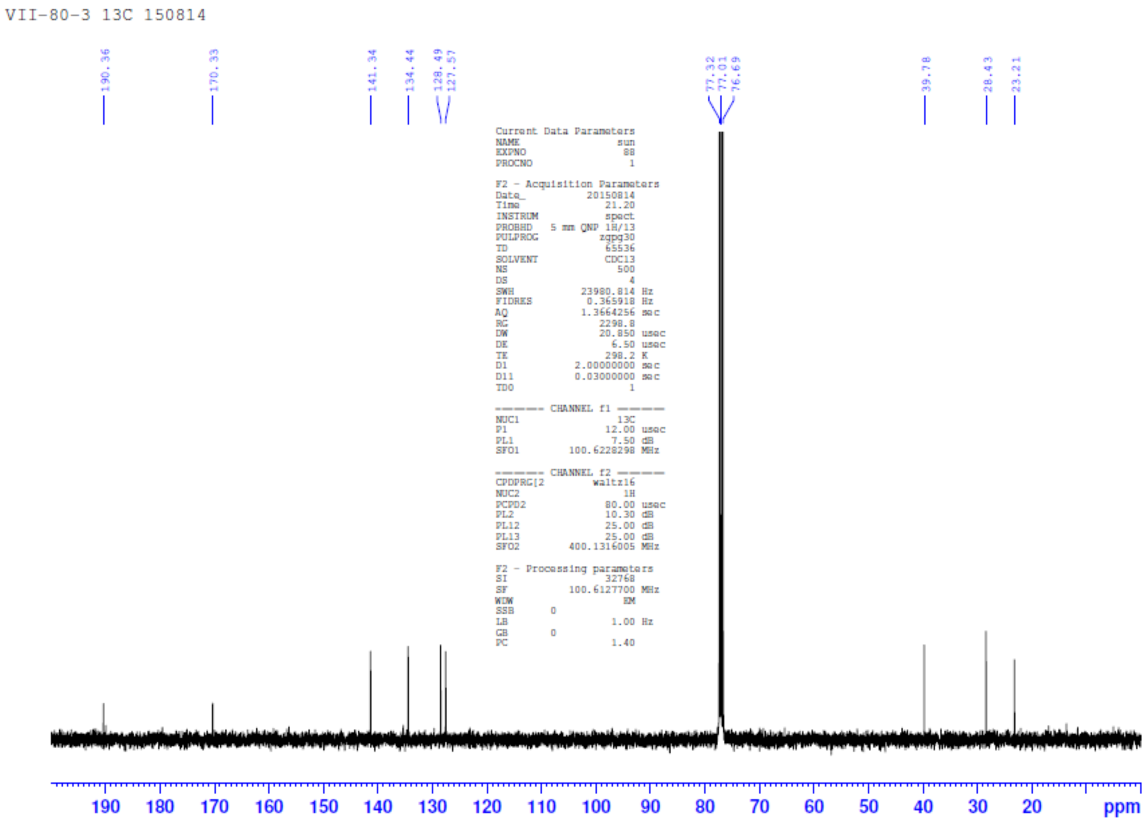

Figure S26. Biological activities

(a) Anti-oomycete (*Phytophthora capsici*)

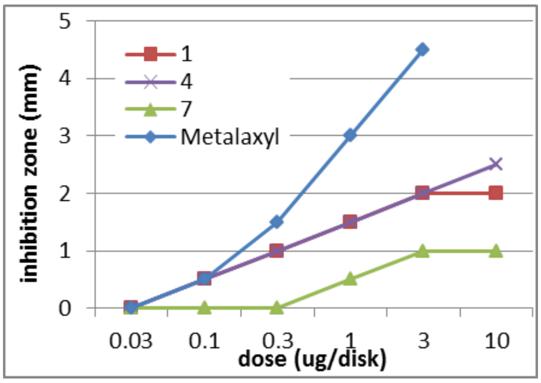

(b) Cytotoxicity (HeLa-S3 cells)

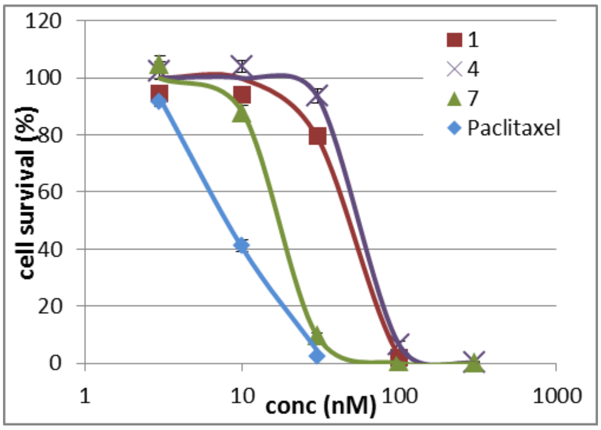

Markers indicate average values with SE, and lines indicate theoretical sigmoid curves.

Supplementary Tables

Table S1. PKS in *hli* for the biosynthesis of haliangicin skeleton.

Gene	Protein	Length (aa)	Type	Domain position
hliF	HliF	1372	Type I PKS	KS (36-458), AT (552-847), *O*-MT (942-1195), ACP (1280-1349)
hliG	HliG	1966	Type I PKS	KS (37-460), AT (594-896), DH (979-1145), KR (1544-1722), ACP (1834-1899)
hliP	HliP	2890	Type I PKS	KS (12-449), ACP (850-923), KS (953-1380), AT (1498-1804), DH (1874-2042), KR (2431-2623), ACP (2733-2803)
hliT	HliT	2225	Type I PKS	KS (36-459), AT (564-853), DH (927-1092), ER (1501-1808), KR (1832-2010), ACP (2111-2181)

Table S2.*β*-methyl branching cassette in *hli* and its homology.

Product	KS	ACP	HCS	ECH	ECH	AT	Tandem ACP	Origin	HCS identity to HliM *4
haliangicin	HliL*1	HliN	HliM	HliO	HliC	HliL	-	*H. ochraceum*	100%
crocacin[11]	CroD*1	CroH	CroE	CroF	CroG	CroD	-	*C. crocatus*	50%
virginiamycin M[12]	VirB	*2	VirC	VirD	VirE	VirI	VirA	*S. virginiae*	37%
myxovirescin[2]	TaK	TaB TaE	TaC TaF	TaX	TaY	TaV	-	*M. xanthus*	34% 35%
corallopyronin A[13]	CorD	CorC	CorE	CorF	CorG	CorA	CorJ/K/L	*C. coralloides*	34%
mupirocin[14]	MupG	Macp15	MupH	MupJ	MupK	MmpIII	MmpI	*P. fluorescens*	34%
batumin[15]	BatB	BatA	BatC	BatD	BatE	BatH	Bat2	*P. fluorescens*	34%

*1 KS domains does not feature the cysteine-to-serine mutation in typical KS[S]

*2 An ACP-encoding orf can be found between *virA* and *virB* but did not present in the original report

Table S3. Methoxymalony-ACP cassette in *hli* and its homology.

	HliQ	HliH	HliI	HliJ	HliK
Proposed function	AT-ACP-*O*-MT	FkbH-like protein	Acyl-CoA dehydrogenase	ACP	3-hydroxyacyl-CoA dehydrogenase
Identity to *pel* biosynthesis[10]	PelG (48%)	PeK (64%)	PelJ (57%)	PelI (54%)	PelH (66%)
Identity to *sor* biosynthesis[6]	SorC (44%)	-	SorE (55%)	-	SorD (59%)
Identity to *fkb* biosynthesis[7]	FkbG (29%)	FkbH (47%)	FkbI (35%)	FkbJ (52%)	FkbK (38%)

Accession: PelG (CCE88382.1), PelK (CCE88385.1), PelJ (CCE88384.1), PelI (CCE88387.1), PelH (CCE88386.1), SorC (AAK19884.1), SorE (AAK19892.1), SorD (AAK19885.1), FkbG (AAF86386.1), FkbH (AAF86387.1), FkbI (AAF86388.1), FkbJ (AAF86389.1), FkbK (AAF86390.1).

Table S4. Bacterial strains used in this study.

Bacterial strains	Description	Source or reference
E. coli		
TOPO 10	TOPO TA Cloning host	Invitrogen
Mach 1	General cloning host	Invitrogen
XL1-Blue	General cloning host	Stratagene
Fusion-Blue	Cloning host for In-fusion cloning	Clontech
SW105	λ-Red recombineering host	NCI, [ref 16]
YM297	Cloning template of tetracycline resistance gene (tet^R)	NBRP, [ref 17]
KRX	Expression host for recombinant proteins	Promega
M. xanthus		
ATCC25232	Wild type, heterologous host of haliangicin biosynthetic gene cluster	Ajinomoto
c7-6E TA-Kan^R	Mutant harboring the sequence of c7-6E TA-Kan^R(5' end of haliangicin biosynthetic gene cluster, from hliA to hliP) , Kan^R, $Thio^R$	This study
c10-11C-c7-6E	Mutant harboring the entire haliangicin biosynthetic gene cluster, Kan^R, $Oxytet^R$, $Thio^R$, haliangicin (**1**) producing	This study
Δ*hliD*	*hliD* disruptant, Kan^R, $Oxytet^R$, $Thio^R$, Nrs^R, 1-*O*-demethylhaliangicin (**6**), 1-*O*-demethyl-12,13-deoxylhaliangicin (**5**) producing	This study
Δ*hliR*	*hliR* disruptant, Kan^R, $Oxytet^R$, $Thio^R$, Nrs^R, 14,15-dihydrohaliangicin (**4**) producing	This study
Δ*hliU*	*hliU* disruptant, Kan^R, $Oxytet^R$, $Thio^R$, Nrs^R, 12,13-deoxylhaliangicin (**7**) producing	This study
H. ochraceum		
SMP-2	original haliangicin, haliangicin stereoisomer producer	Ajinomoto

Table S5. Vectors used in this study.

Plasmids	Description	Source or reference
General vectors		
pGEM-T easy vector	General TA cloning vector, Amp^R	Promega
pGEM-T vector	General TA cloning vector, Amp^R	Promega
pCR 2.1-TOPO	General TA cloning vector, Amp^R, Kan^R	Invitrogen
pHSG398	General cloning vector, Cm^R	Takara
pET-32a	Expression vector for thioredoxin and His_6-fusion recombinant protein, Amp^R	Novagen
pNR1	Cloning template of nourseothricin resistance gene, Nrs^R	ref 18
pHSG398 Infu Nrs^R	General cloning vector for construction of disruption vector, pHSG398-derived, Nrs^R under Tn5 promoter	This study
Disruption vectors	Description	Transformant
pHSG GemOMT4 Infu Nrs^R	pHSG398 Infu Nrs^R-derived, carrying internal fragment of *hliD* (522 bp), Nrs^R	*M. xanthus* Δ*hliD*
pHSG 18ACAD Infu Nrs^R	pHSG398 Infu Nrs^R-derived, carrying internal fragment of *hliR* (715 bp), Nrs^R	*M. xanthus* Δ*hliR*
pHSG 21Ox Infu Nrs^R	pHSG398 Infu Nrs^R-derived, carrying internal fragment of *hliU* (730 bp), Nrs^R	*M. xanthus* Δ*hliU*
Expression vectors	Description	Expressed protein
pET32a HliR	pET-32a-derived, expression vector for N-His_6-fusion HliR, Amp^R	HliR
Other vectors	Description	Source
pTA	pGEM-T easy vector-derived, carrying TA fragment (1.8 kb), Amp^R	This study
pTA-Kan^R	pTA-derived, carrying TA-Kan^R fragment (2.7 kb), Kan^R, Amp^R	This study
pGem walking	pGEM-T vector-derived, carrying 7.4 kb fragment for gene walking, Amp^R	This study
Cosmids		
pDW103	*E. coli*/*Streptomyces* shuttle cosmid vector, Amp^R, $Thio^R$	ref 19
c7-6E	pDW103-derived, harboring 5' side of haliangicin biosynthetic gene fragment, Amp^R, $Thio^R$	This study
c7-6E TA-Kan^R	Modified c7-6E, harboring TA fragment, Kan^R, $Thio^R$	This study
c10-11C	pDW103-derived, harboring 3' side of haliangicin biosynthetic gene fragment, Amp^R, $Thio^R$	This study
c10-11C Tet^R	Modified c10-11C, $Oxytet^R$, $Thio^R$	This study

Table S6. Primers used in this study.

Primers	Sequence (5' → 3')
KS1UP	MG[I]GARGC[I]HW[I]SM[I]ATGGAYCC[I]CARCA[I]MG
KSD1	GGRTC[I]CC[I]AR[I]SW[I]GT[I]CC[I]GT[I]CCRTG
DWF1	GGCCGGGTCCCTCCGCCGCA CGGAG
DWR	GACAGTCATAAGTGCGGCGACGATAG
blaF-TAf	ATGGTTTCTTAGACGTCAGGTGGCACTTTTCGGGGAAATGTGCGCGGAACAGT GAGAGGTTCGGGTGGT
blaR-kan1	GCTTAATCAGTGAGGCACCTATCTCAGCGATCTGTCTATTTCGTTCATCCTGCA GGGCGCAAGGGCTGCTAA
TAf	AACAGTGAGAGGTTCGGGTGGT
TAr	CTATCTGCTCGACCTTCACGGG
PstI-Kan1	TCCCATATGGTCGACCTGCAGGGCGCAAGGGCTGCTA AAGG
SpeI-Kan2	AGATAGAATCACTAGTGCACCCAACTGATCTTCAGCA
blaF-tetU	ATGGTTTCTTAGACGTCAGGTGGCACTTTTCGGGGAAATGTGCGCGGAACGTT GGAGCCGCATTATTTTCGC
blaR-tetD	GCTTAATCAGTGAGGCACCTATCTCAGCGATCTGTCTATTTCGTTCATCCAGGG TCATTATATTTCGCGGAATAAC
ORF4-MT-f1	ACACCTTCGAGATCACTGCG
ORF4-MT-r1	GACCTACGACCTGGTTCTGC
pHSG18ACAD Infu-f	CCGGGGATCCTCTAGTGGACATCTACGAGGCGTTTC
pHSG18ACAD Infu-r	GGCCAGTGCCAAGCTGCGCCGAACACGTGATTGTT
pHSG 21Ox Infu F	CCGGGGATCCTCTAGTAGGGCGGAGCTGGAGATG
pHSG 21Ox Infu R	GGCCAGTGCCAAGCTGAAGTGCCGACGATGATTGC
HliR Exp Infu f	GGCTGATATCGGATCCATGAGCGCAGATACAACCAAG
HliR Exp Infu r	GGTGGTGGTGCTCGATTCCATGTCGCTCGGTGTC
thioF	TTTGCCAGAGAGCGACGACTTCC
tetU	ACGTTGGATCCGCATTATTTTC GC
TAf-F	GGTAGCGCCCCGACATGCCGA
HpaI-kan1	GATATGTTAACTGCAGGGCGCAAGGGCTGCTAA
blaF	ATGGTTTCTTAGACGTCAGG
TAr-R	GTGAAGTGGGGCGGCTTCAT
9-4F-SorDF	TACTCGGAATGCATTTCATG
9-4F-SorDR	GTCCGGAAAGTTGTCGAGAT
HMG-Syn Up	TCGCCGAGCAAGCTCAGCAGCGACA
HMG-syn D	ATGCTGACGGCGGCG TTGCGCGAGGA
HKS4NU	GTCGACCGCTTCGATCCCTTTCCGTTTCGC
HKS4ND	GGCCATTCTGATTG ACGGACGAACCGCGCA
1011R-U	CCGACGTTGCCGGCCAGGACCTG
1011R-D	TCATCGACGGCCTGTTGCGCGTG
7-6ENF-F	CTTGGTCT CATCACTCGCGACTGC
7-6ENF-R	GCGCTCGCGTTGCGTCCTGATG
pHSG398 Inverse-F	CCCTTAAACGCCTGGTGCTA
pHSG398 Inverse-R	GGAACCTCTTACGTGCCGAT
Kan Nrs-f	GCTATGACTGGGCACCCCTTTCTCTCCTGGAAGACT
Amp Nrs-r	GATACGGG AGGGCTTACATCCACGGGACTTGAGAC
M13f(-47)	CGCCAGGGTTTTCCCAGTCACGAC
M13r(-48)	AGCGGATAACAATTTCACACAGGA
ORF18ACAD-Out f	ATCTTCCGCAATCAGCAGGA
ORF18ACAD-Out r	GACGTTTGTCCGCTTTCGAT
ORF4-MT-Ex-f1	ATGTCTGGCAAGAGCAGGTC
ORF4-MT-Ex-r1	TCATGCGACTGGACGAACAA
ORF21Ox-Out F2	GAGCATCTCGGGACTTACGC
ORF21Ox-Out R	GCTGGGGGTCATAGAGAAGG

Table S7. Analysis of end sequences of five cosmids that contain KS domains.

cosmids	primers	Top-hit homology of the translated end sequence	Similarity/ Identity
c3-5F	forward	ubiquinone biosynthesis protein [uncultured archaeon GZfos27G5]	46%/31%
	reverse	β-ketoacyl synthase, PpsD [*Mycobacterium ulcerans* Agy99]	74%/62%
c4-6B	forward	protein kinase [*Sorangium cellulosum* So ce 56]	53%/40%
	reverse	3-hydroxy-3-methylglutaryl-CoA synthase [*Nostoc pinctiforme* PCC 73102]	68%/50%
c7-6E	forward	StiF protein [*Stigmatella aurantiaca*]	46%/32%
	reverse	similar to hamlet CG31753-PA [*Apis mellifera*]	39%/32%
c9-4F	forward	polysaccharide deacetylase [uncultured archaeon GZfos1D1]	53%/35%
	reverse	3-hydroxybutyryl-CoA dehydrogenase [*Herpetosiphon aurantiacus* ATCC 23779]	40%/33%
c10-11C	forward	3-hydroxybutyryl-CoA dehydrogenase [*Herpetosiphon aurantiacus* ATCC 23779]	85%/71%

Table S8. NMR data for unnatural haliangicin analogs in $CDCl_3$.

	14,15-dihydrohaliangicin (**4**)		12,13-deoxylhaliangicin (**7**)	
Position	δ_H (mult, *J* in Hz)	δ_C	δ_H (mult, *J* in Hz)	δ_C
1	-	167.0	-	167.1
2	5.25 (s)	95.7	5.25 (s)	95.7
3	-	165.8	-	165.9
4	-	148.1	-	148.0
5	5.78 (s)	112.2	5.80 (s)	112.5
6	-	130.5	-	130.1
7	6.03 (d, 11.2)	126.3	6.04 (d, 11.2)	126.5
8	6.11 (d, 11.2)	120.3	6.09 (d, 12.0)	120.3
9	-	142.0	-	142.5
10	2.33 (m)	40.4	2.41 (m)	41.5
11	1.21 (dd, 9.2, 13.6)	44.3	1.99 (dd, 8.4, 13.2)	46.0
	1.95 (dd, 6.0, 13.6)		2.18 (dd, 6.8, 13.6)	
12	-	60.2	-	138.2
13	2.68 (t, 6.4)	65.1	5.81 (d, 10.4)	127.0
14a	1.48 (m)	22.0	6.54 (td, 10.4, 16.8)	133.4
14b	1.60 (m)			
15	1.01 (t, 7.6)	10.5	4.96 (dd, 1.6, 10.4)	114.6
			5.07 (dd, 1.6, 16.8)	
1-OMe	3.67 (s)	51.2	3.68 (s)	51.3
3-OMe	3.72 (s)	56.0	3.73 (s)	56.0
4-OMe	3.55 (s)	57.2	3.55 (s)	57.2
6-Me	2.09 (s)	23.6	2.09 (s)	23.6
9-Me	1.69 (s)	13.5	1.70 (s)	13.4
10-Me	1.00 (d, 6.8)	19.2	0.96 (d, 6.8)	18.9
12-Me	1.23 (s)	16.6	1.73 (s)	16.5

References

1. Sharan, S.K., Thomason, L.C., Kuznetsov, S.G. & Court, D.L. *Nat. Protoc.* **4**, 206-223 (2009).
2. Simunovic, V. et al. *Chembiochem* **7**, 1206-1220 (2006).
3. Yu, T.W. et al. *Proc. Natl. Acad. Sci. U. S. A.* **99**, 7968-7973 (2002).
4. Wenzel, S.C. et al. *J. Am. Chem. Soc.* **128**, 14325-14336 (2006).
5. Hill, A.M., Harris, J.P. & Siskos, A.P. *Chemical Communications*, 2361-2362 (1998).
6. Ligon, J. et al. *Gene* **285**, 257-267 (2002).
7. Wu, K., Chung, L., Revill, W.P., Katz, L. & Reeves, C.D. *Gene* **251**, 81-90 (2000).
8. Calderone, C.T. *Nat. Prod. Rep.* **25**, 845-853 (2008).
9. Muller, S. et al. *Chem. Biol.* **21**, 855-65 (2014).
10. Jahns, C. et al. *Angew. Chem.-Int. Edit.* **51**, 5239-5243 (2012).
11. Muller, S. et al. *Chem. Biol.* **21**, 855-865 (2014).
12. Pulsawat, N., Kitani, S. & Nihira, T. *Gene* **393**, 31-42 (2007).
13. Erol, O. et al. *Chembiochem* **11**, 1253-1265 (2010).
14. Gurney, R. & Thomas, C.M. *Appl. Microbiol. Biotechnol.* **90**, 11-21 (2011).
15. Mattheus, W. et al. *Chem. Biol.* **17**, 149-159 (2010).
16. Warming, S., Costantino, N., Court, D.L., Jenkins, N.A. & Copeland, N.G. *Nucleic Acids Res.* **33**, e36 (2005).
17. Yamazoe, M. et al. *Embo J.* **18**, 5873-5884 (1999).
18. Malonek, S., Rojas, M.C., Hedden, P., Hopkins, P. & Tudzynski, B. *Appl. Environ. Microbiol.* **71**, 6014-6025 (2005).
19. Hyun, C.G. et al. *FEMS Microbiol. Lett.* **183**, 183-189 (2000).
